# Receptor deorphanization in an echinoderm reveals kisspeptin evolution and relationship with SALMFamide neuropeptides

**DOI:** 10.1186/s12915-022-01387-z

**Published:** 2022-08-24

**Authors:** Nayeli Escudero Castelán, Dean C. Semmens, Luis Alfonso Yañez Guerra, Meet Zandawala, Mario dos Reis, Susan E. Slade, James H. Scrivens, Cleidiane G. Zampronio, Alexandra M. Jones, Olivier Mirabeau, Maurice R. Elphick

**Affiliations:** 1 Queen Mary University of London, School of Biological & Behavioural Sciences, London, E1 4NS UK; 2 Present address: Institute of Medical and Biomedical Education, St George’s University of London, Cranmer Terrace, London, SW17 0RE UK; 3 Present Address: Living Systems Institute, University of Exeter, Exeter, EX4 4QD UK; 4 Present Address: Neurobiology and Genetics, Theodor-Boveri-Institute, Biocenter, University of Würzburg, 97074 Würzburg, Germany; 5 School of Life Sciences, University of Warwick, Coventry, CV4 7AL UK; 6 Present address: Waters Corporation, Stamford Avenue, Altrincham Road, Wilmslow, SK9 4AX UK; 7 Present address: School of Science, Engineering & Design, Stephenson Street, Teesside University, Middlesbrough, TS1 3BX, TS1 3BA Tees Valley UK; 8 Institut Pasteur, Université Paris Cité, Bioinformatics and Biostatistics Hub, 75015 Paris, France

**Keywords:** Neuropeptide, Evolution, Kisspeptin, SALMFamide, Starfish

## Abstract

**Background:**

Kisspeptins are neuropeptides that regulate reproductive maturation in mammals via G-protein-coupled receptor-mediated stimulation of gonadotropin-releasing hormone secretion from the hypothalamus. Phylogenetic analysis of kisspeptin-type receptors indicates that this neuropeptide signaling system originated in a common ancestor of the Bilateria, but little is known about kisspeptin signaling in invertebrates.

**Results:**

Contrasting with the occurrence of a single kisspeptin receptor in mammalian species, here, we report the discovery of an expanded family of eleven kisspeptin-type receptors in a deuterostome invertebrate — the starfish *Asterias rubens* (phylum Echinodermata). Furthermore, neuropeptides derived from four precursor proteins were identified as ligands for six of these receptors. One or more kisspeptin-like neuropeptides derived from two precursor proteins (ArKPP1, ArKPP2) act as ligands for four *A. rubens* kisspeptin-type receptors (ArKPR1,3,8,9). Furthermore, a family of neuropeptides that act as muscle relaxants in echinoderms (SALMFamides) are ligands for two *A. rubens* kisspeptin-type receptors (ArKPR6,7). The SALMFamide neuropeptide S1 (or ArS1.4) and a ‘cocktail’ of the seven neuropeptides derived from the S1 precursor protein (ArS1.1-ArS1.7) act as ligands for ArKPR7. The SALMFamide neuropeptide S2 (or ArS2.3) and a ‘cocktail’ of the eight neuropeptides derived from the S2 precursor protein (ArS2.1-ArS2.8) act as ligands for ArKPR6.

**Conclusions:**

Our findings reveal a remarkable diversity of neuropeptides that act as ligands for kisspeptin-type receptors in starfish and provide important new insights into the evolution of kisspeptin signaling. Furthermore, the discovery of the hitherto unknown relationship of kisspeptins with SALMFamides, neuropeptides that were discovered in starfish prior to the identification of kisspeptins in mammals, presents a radical change in perspective for research on kisspeptin signaling.

**Supplementary Information:**

The online version contains supplementary material available at 10.1186/s12915-022-01387-z.

## Background

Kisspeptins are a family of neuropeptides that were first discovered in mammals and found to act as ligands for the orphan G-protein-coupled receptor GPR54 (KissR1) [1]. Kisspeptins are derived from the precursor protein Kiss1 and range in length from fifty-four to ten residues (KP54, KP14, KP13, KP10) [1–3], with a common C-terminal region being required for receptor activation [1, 2, 4].

A key insight into the physiological roles of kisspeptin signaling in mammals was the discovery that mutations in the kisspeptin receptor KissR1 cause impaired pubertal maturation and reproductive function in humans [5, 6]. Accordingly, knockout of either the Kiss1 or the Kiss1R genes in mice causes delayed pubertal onset and subsequent infertility [6–8]. Thus, kisspeptin signaling is now recognized as an important regulator of reproductive development, function, and behavior in mammals [9–12]. Subsequently, other physiological roles of kisspeptins have been discovered, including roles in the regulation of energy balance, metabolism, and glucose homeostasis [13–15].

Analysis of the phylogenetic distribution of kisspeptin signaling has identified genes encoding kisspeptin precursors and receptors in non-mammalian vertebrates but with variation in the number of genes in different species [16–20]. Furthermore, phylogenomic analysis indicates that the common ancestor of vertebrates had a single kisspeptin precursor and a single kisspeptin receptor and two rounds of genome duplication during early vertebrate evolution gave rise to four kisspeptin precursor genes and four kisspeptin receptor genes. Then, differential loss of one or more of these genes in vertebrate lineages gave rise to the variety in the number of kisspeptin precursors and kisspeptin receptors in extant species [18, 19, 21], with complete loss in birds [22].

Genes encoding kisspeptin-type receptors have been identified outside vertebrates in non-vertebrate deuterostomes (cephalochordates, echinoderms) and in protostomes (mollusks, annelids) [17, 23–25]. Therefore, the evolutionary origin of kisspeptin-type neuropeptide signaling can be traced back to the common ancestor of the Bilateria. However, as in some vertebrate lineages, there has been a loss of kisspeptin-type signaling in several invertebrate taxa, including arthropods and tardigrades [26–30]. Conversely, multiple gene duplications have given rise to expanded kisspeptin-type signaling systems in some taxa. For example, sixteen kisspeptin-type receptors and four precursors of kisspeptin-type neuropeptides were identified in the invertebrate chordate *Branchiostoma floridae* (phylum Cephalochordata) [16, 17]. Subsequently, an ortholog of one of the *B. floridae* kisspeptin-type receptors has been characterized in *Branchiostoma japonicum* (BjGPR54L-1) and the neuropeptide BjKissL-2 has been identified as a ligand for BjGPR54L-1 [20]. Furthermore, investigation of the physiological roles of kisspeptin signaling in *B. japonicum* has provided evidence of an evolutionarily conserved role in regulation of reproductive function in chordates [20].

Currently, little is known about the neuropeptides that act as ligands for kisspeptin-type receptors outside chordates. However, a precursor of two kisspeptin-like peptides has been identified in the starfish *Asterias rubens* [31], and orthologs of this precursor have been identified in other echinoderms, including brittle stars, sea urchins, and sea cucumbers [32]. Furthermore, kisspeptin-like peptides act as ligands for two kisspeptin-type receptors in the sea cucumber *Apostichopus japonicus* [33].

Here, we report the discovery of an expanded family of eleven kisspeptin-type receptors in the starfish *A. rubens* and the identification of neuropeptides that act as ligands for six of these receptors. These include kisspeptin-like neuropeptides derived from two precursor proteins — ArKPP1 and ArKPP2. Furthermore, and importantly, we have discovered that two kisspeptin-type receptors in *A. rubens* (ArKPR6 and ArKPR7) are activated by SALMFamides, a family of neuropeptides that act as muscle relaxants in echinoderms and which were first identified in starfish prior to the discovery of kisspeptins in mammals [34, 35]. These findings provide important new insights into the evolution and diversity of kisspeptin signaling.

## Results

### Identification and phylogenetic analysis of echinoderm kisspeptin-type receptors

To investigate the occurrence and pharmacological properties of kisspeptin-type receptors in echinoderms, we selected the common European starfish *A. rubens* as our experimental model. The primary reasons for this selection were as follows. Firstly, we have obtained neural transcriptome sequence data for this species, which we have used to identify transcripts encoding over forty neuropeptide precursor proteins, including a kisspeptin-type precursor, and several G-protein-coupled neuropeptide receptors [31, 36–43]. Secondly, a chromosomal assembly of the genome sequence of *A. rubens* has been obtained recently [44], providing a valuable resource for the comprehensive investigation of the occurrence of genes encoding kisspeptin-type receptors and kisspeptin-type precursor proteins in this species. Thirdly, we have performed a mass spectrometric analysis of radial nerve cord extracts from this species, which has enabled the determination of the mature structure of neuropeptides, as reported previously for many other neuropeptide types [36–40, 42, 43, 45–47] and as reported here for kisspeptin-related neuropeptides. Lastly, we have established *A. rubens* as an experimental model for functional characterization of neuropeptide signaling systems [36, 38, 40, 41, 43, 45–49], which provides a strong basis for future investigations of the physiological roles of kisspeptin-type neuropeptides in this species.

**Fig. 1:**
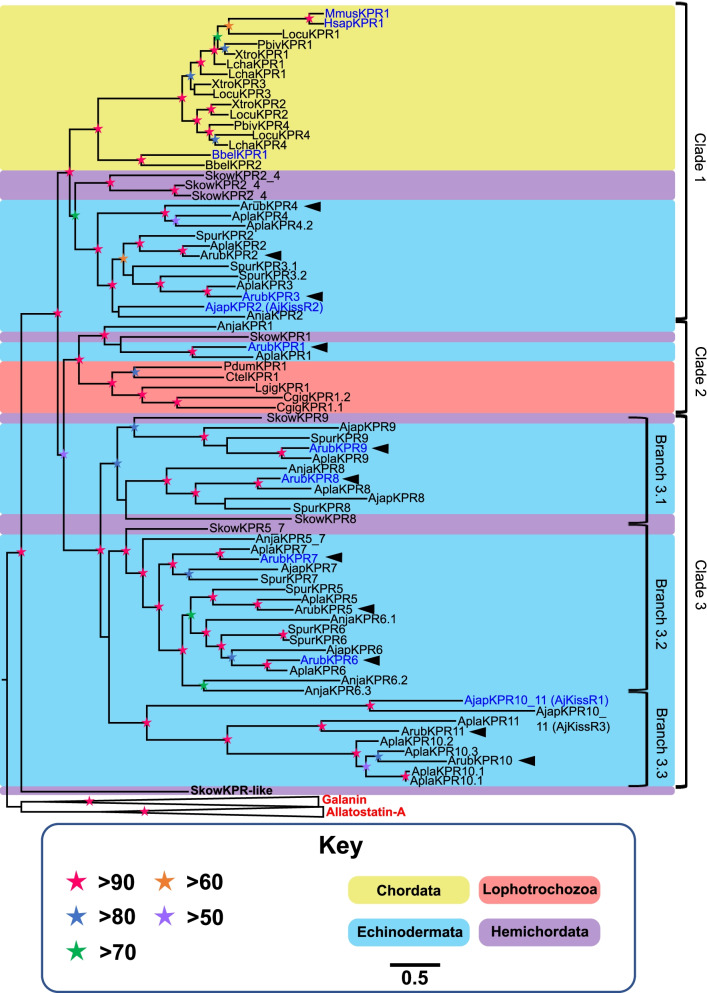
Phylogenetic analysis of bilaterian kisspeptin-type receptors, including *A. rubens* receptors ArKPR1-11. The phylogenetic tree, which was constructed using the maximum-likelihood method and rooted with galanin/allatostatin-A-type receptors as an outgroup, comprises three distinct clades, with bootstrap support > 90. Clade 1 includes ArKPR2-4 (ArubKPR2-4) and chordate kisspeptin-type receptors. Clade 2 includes ArKPR1 and protostome (annelid, mollusk) kisspeptin-type receptors. Clade 3 comprises three sub-clades: clade 3.1 includes ArKPR8-9 (ArubKPR8-9) and related receptors in other echinoderms and the hemichordate *S. kowalevskii*, clade 3.2 includes ArKPR5-7 (ArubKPR5-7) and related receptors in other echinoderms and the hemichordate *S. kowalevskii*, and clade 3.3 includes ArKPR10-11 (ArubKPR10-11) and related receptors in other echinoderms. The stars represent branch support (bootstrap 1000 replicates) and the pastel-colored backgrounds represent taxonomic groups (see key). The arrowheads label the eleven *A. rubens* kisspeptin-type receptors ArKPR1-11. The scale bar indicates amino acid substitutions per site. Receptors for which ligands have been identified experimentally in this study or other published studies are colored in blue. Species names are as follows: Apla, *Acanthaster planci*; Ajap, *Apostichopus japonicus*; Arub, *Asterias rubens*, Anjap *Annessia japonica*; Bbel, *Branchiostoma belcheri*; Ctel, *Capitella teleta*; Cgig, *Crassostrea gigas*; Hsap, *Homo sapiens*; Lcha, *Latimeria chalumnae*; Locu, *Lepisosteus oculatus*; Lgig, *Lottia gigantea*; Mmus, *Mus musculus*; Pdum, *Platynereis dumerilii*; Pbiv, *Python bivittatus*; Skow, *Saccoglossus kowalevskii*; Spur, *Strongylocentrotus purpuratus*; Xtro, *Xenopus tropicalis*. Accession numbers for the sequences of the receptors included in this tree are listed in Additional file 3

Analysis of transcriptome/genome sequence data revealed the presence of eleven genes/transcripts encoding putative kisspeptin-type receptors in the starfish *A. rubens* (ArKPR1-11; Additional file 1). Having identified putative kisspeptin-type receptors in *A. rubens* (class Asteroidea), we then investigated the occurrence of kisspeptin-type receptors in other echinoderms. Thirteen were identified in the crown-of-thorns starfish *Acanthaster planci* (class Asteroidea), eight were identified in the sea urchin *Strongylocentrotus purpuratus* (class Echinoidea), seven were identified in the sea cucumber *A. japonicus* (class Holothuroidea), and seven were identified in the feather star *Anneissia japonica* (class Crinoidea) (Additional files 2 and 3). Furthermore, we also investigated the occurrence of kisspeptin-type receptors in species belonging to other bilaterian phyla (Additional files 2 and 3). To investigate the relationships of the putative echinoderm kisspeptin-type receptors with receptors in other bilaterians, a phylogenetic tree was generated (Fig. 1) using the maximum-likelihood method [50, 51]. In addition to kisspeptin-type receptors, also included in this analysis were galanin-type and allatostatin-A-type receptors from a variety of taxa because previous studies have revealed that these receptors are closely related to kisspeptin-type receptors [17, 52]. Thus, the objective was to determine if the putative echinoderm kisspeptin-type receptors are indeed kisspeptin-type receptors or are more closely related to galanin/allatostatin-A-type receptors. Having rooted the tree with galanin-type and allatostatin-A-type receptors, robust bootstrap support (> 90%) was obtained for a branch of the tree comprising kisspeptin-type receptors that also contains all eleven *A. rubens* receptors (ArKPR1 to ArKPR11) (Fig. 1), indicating that ArKPR1-11 are indeed kisspeptin-type receptors and not galanin-type or allatostatin-A-type receptors.

A more detailed analysis of the phylogenetic tree reveals that the kisspeptin-type receptors sub-divide into three clades (clades 1–3), each with bootstrap support > 90%. However, bootstrap support (< 60%) was not high enough to determine with confidence relationships between these three clades. Clade 1 includes the *A. rubens* receptors ArKPR2-4, closely related receptors in other echinoderms and the hemichordate *Saccoglossus kowalevskii*, vertebrate kisspeptin-type receptors, and two kisspeptin-type receptors in the cephalochordate *B. belcheri*. An expanded family of kisspeptin-type receptors has been identified in the cephalochordate *B. floridae* [16, 17], but these are not included in Fig. 1 to ensure readability. However, a tree including these receptors is presented in Additional file 4 and this shows that all of the *B. floridae* kisspeptin-type receptors are positioned in a clade corresponding with clade 1 of Fig. 1. Clade 2 includes ArKPR1, closely related receptors in other echinoderms and the hemichordate *S. kowalevskii*, and kisspeptin-type receptors in mollusks and annelids. Clade 3 comprises three branches: Branch 3.1 (bootstrap support > 80%) includes ArKPR8-9 and closely related receptors in other echinoderms and the hemichordate *S. kowalevskii*. Branch 3.2 (bootstrap support > 90%) includes ArKPR5-7 and closely related receptors in other echinoderms and the hemichordate *S. kowalevskii*. Branch 3.3 (bootstrap support > 90%) includes ArKPR10-11 and the *A. japonicus receptors* AjapKPR10_11 (AjKissR1) and AjapKPR10_11 (AjKissR3). However, because the branch lengths for the receptors in branch 3.3 are quite long, we performed an additional phylogenetic analysis using both maximum likelihood and Bayesian inference methods (Additional files 5 and 6), which revealed instability in the phylogenetic positions of AjapKPR10_11 (AjKissR1) and AjapKPR10_11 (AjKissR3). Furthermore, these analyses also revealed method-dependent variability in the phylogenetic positions of two of the *B. floridae* kisspeptin-type receptors, which are positioned either in clade 1 (Additional file 6) or in clade 2 (Additional file 5). Collectively, our phylogenetic analysis shows that there is a large and diverse family of kisspeptin receptor-related proteins in starfish and other echinoderms, which comprise three clades. Interestingly, clades 1–3 also contain receptors from a hemichordate species (*S. kowalevskii*), indicating that the gene duplication events that gave rise to these three clades occurred prior to the existence of a common ancestor of the Ambulacraria, a superphylum that comprises echinoderms and hemichordates. In contrast, chordates only have kisspeptin-type receptors positioned in clade 1 or 2 and lophotrochozoan phyla (annelids and mollusks) only have kisspeptin-type receptors in clade 2 (Fig. 1).

To complement the phylogenetic analysis, we also performed an analysis of receptor relationships using the CLuster ANalysis of Sequences (CLANS) method. First, CLANS was used to analyze relationships between receptors identified by the Basic Local Alignment Serach Tool (BLAST) analysis of bilaterian genomic sequence data (Additional files 2 and 7), which facilitated the determination of the occurrence of putative kisspeptin-type receptors in bilaterian taxa. Then, CLANS was used for a more specific analysis of relationships between the receptors included in Fig. 1 (Additional file 8). Consistent with the phylogenetic tree (Fig. 1), CLANS revealed that ArKPR1-9 are positioned in a cluster containing kisspeptin-type receptors that are distinct from a cluster containing galanin/allatostatin-A-type receptors. Furthermore, it is noteworthy that ArKPR10 and ArKPR11 are positioned distal to the main cluster of kisspeptin-type receptors, indicative of sequence divergence and consistent with the long branches to these receptors in Fig. 1. Accordingly, the *A. japonicus* receptors AjapKPR10_11 (AjKissR1) and AjapKPR10_11 (AjKissR3) are also are positioned distal to the main cluster of kisspeptin-type receptors, consistent with instability in the position of these receptors in phylogenetic analyses (Fig. 1; Additional files 4, 5, and 6).

### Structure and chromosomal location of genes encoding kisspeptin-type receptors in *A. rubens*

**Fig. 2:**
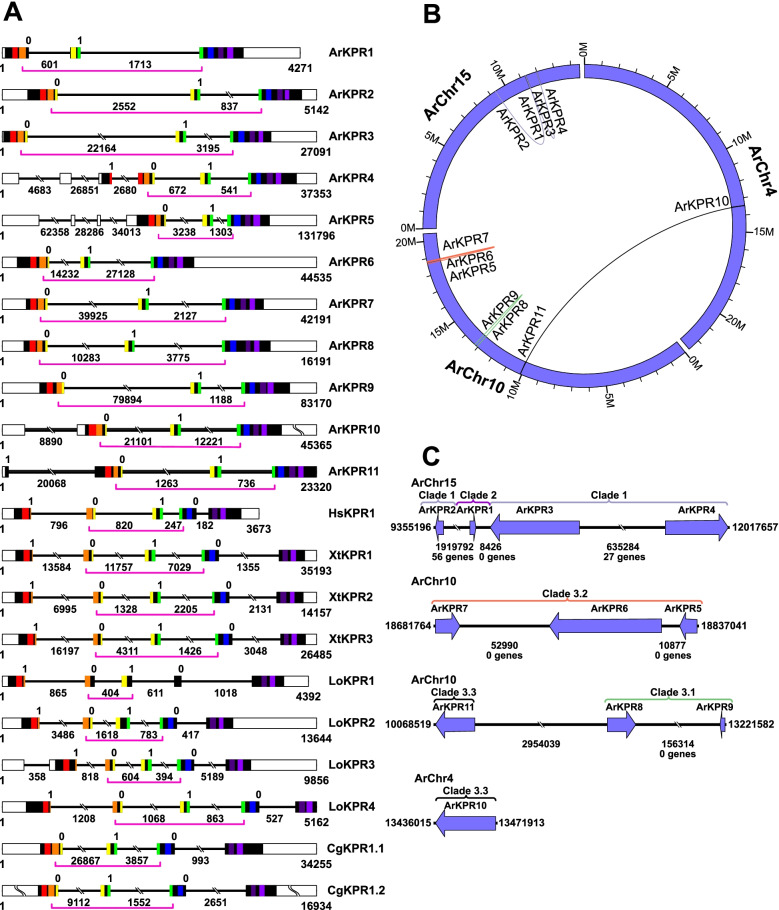
Comparative analysis of the structure and chromosomal location of genes encoding kisspeptin-type receptors. **A** Comparison of the exon/intron structure of genes encoding kisspeptin-type receptors in *A. rubens *(*Ar*), *Homo sapiens *(*Hs*), *Xenopus tropicalis* (Xt), *Lepisosteus oculatus* (Lo), and *Crassostrea gigas *(Cg). Exons are shown as rectangles, with non-coding regions white and protein-coding regions black or colored (regions encoding predicted transmembrane domains 1–7 are shown in red, orange, yellow, green, blue, dark purple, and light purple, respectively). Introns are shown as lines, with length underneath. The two introns highlighted with a pink bracket are a conserved feature (see also Additional file 9). Accession numbers for the transcripts/genes represented in this figure are in Additional file 2. **B** Diagram showing the chromosomal locations of genes encoding ArKPR1-11 in *A. rubens*. Genes encoding (i) ArKPR1-4, (ii) ArKPR5-9; ArKPR11, and (iii) ArKPR10 are located on chromosomes 15, 10, and 4, respectively. Curved lines linking genes correspond to clades in Fig. 1: light purple is clade 1, green is clade 3.1, orange is clade 3.2, and black is clade 3.3. **C** Diagram showing the relative locations and orientations of genes encoding (i) ArKPR1-4 (clade 1 and 2 of Fig. 1) in a region of chromosome 15, (ii) ArKPR5-7 (clade 3.2 of Fig. 1) in a region of chromosome 10, (iii) ArKPR8-9 (clade 3.1 and 3.3 of Fig. 1) in a region of chromosome 10, and (iv) ArKPR10 (clade 3.3 of Fig. 1). The length (including exons and introns) and orientation of the genes is indicated by the purple arrows and the distance between genes (number of bases) is stated underneath the intervening black lines. The absence (0 genes) or presence (number of genes) of other genes in between those encoding the kisspeptin-type receptors is also stated (see Additional file 11 for data)

Previous studies have revealed evolutionary conservation of the occurrence and positions of introns in genes encoding orthologous neuropeptide receptors in the Bilateria [17]. Therefore, we analyzed the exon/intron structure of genes encoding kisspeptin-type receptor proteins in *A. rubens* and in other taxa, including three vertebrate species (*H. sapiens*, *X. tropicalis*, and *L. oculatus*) and a protostome, the mollusk *Crassostrea gigas* (Fig. 2A; Additional file 9). This revealed that a conserved feature of all kisspeptin-type receptor genes in *A. rubens* and in other taxa are two introns that interrupt the coding sequences corresponding to the third and fourth predicted transmembrane domains of the encoded proteins, which are shown in yellow and green, respectively, in Fig. 2A. Furthermore, and importantly, the phase of the introns (0 and 1, respectively) is also conserved. However, these conserved features of the kisspeptin-type receptor genes are not seen in genes encoding the closely related galanin-type and allatostatin-A-type receptors in bilaterians (Additional file 10). Therefore, these findings provide further evidence that ArKPR1-11 are members of a bilaterian family of kisspeptin-type receptors that evolved from a common ancestral gene.

Analysis of the chromosomal locations of the genes encoding ArKPR1-11 in *A. rubens* provided important additional insights into evolutionary relationships, complementing the phylogenetic analysis of their protein sequences shown in Fig. 1. Thus, the four genes encoding ArKPR1-4 are all located on chromosome 15 and the six genes encoding ArKPR5-9 and ArKPR11 are all located on chromosome 10 and the gene encoding ArKPR10 is located on chromosome 4 (Fig. 2B, C). Furthermore, some genes that are closely related based on phylogenetic sequence analysis (as shown in Fig. 1) are located proximally on chromosomes. For example, ArKPR5-7 are all positioned within clade 3.2 of the phylogenetic tree in Fig. 1, and in accordance with this close evolutionary relationship, the three genes encoding these proteins are located in tandem (i.e., without other intervening genes; see Additional file 11) within a region of chromosome 10 spanning 155,277 bases (Fig. 2B, C). Likewise, ArKPR8-9, which are positioned in clade 3.1 of the phylogenetic tree in Fig. 1, are encoded by genes located in tandem in another region of chromosome 10 separated by 175,704 bases (Fig. 2B, C). These findings indicate how gene duplication has given rise to chromosomal clusters of paralogous genes encoding kisspeptin-type receptors in *A. rubens*.

### Identification of precursors of neuropeptides in echinoderms that share sequence similarity with kisspeptins

**Fig. 3:**
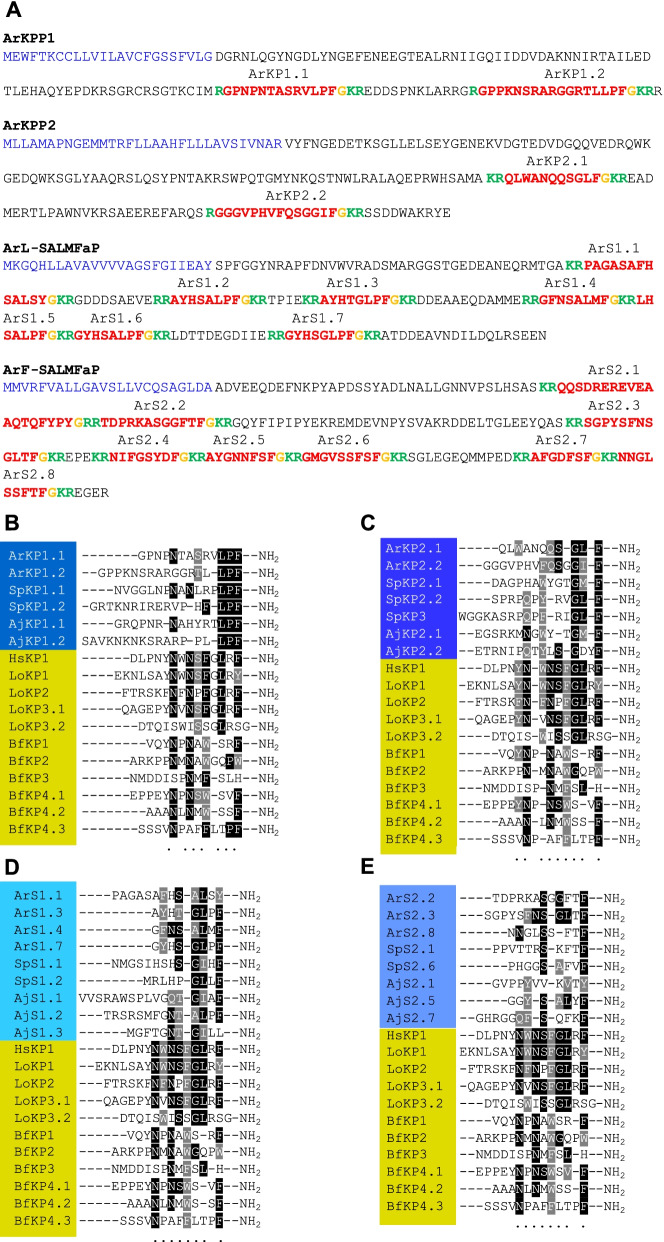
Neuropeptides identified as candidate ligands for kisspeptin-type receptors in echinoderms aligned with chordate kisspeptins. **A** Sequences of the *A. rubens* kisspeptin-type precursors ArKPP1 and ArKPP2 and SALMFamide-type precursors ArL-SALMFaP and ArF-SALMFaP. N-terminal signal peptide is shown in blue, neuropeptides predicted or shown to be derived from these proteins are shown in red (with C-terminal glycine that is a substrate for amidation shown in orange) and monobasic or dibasic cleavage sites are shown in green. Neuropeptides (red) are named in accordance with the precursor they are derived from and their relative position in the precursor, which explains the nomenclature used in **B**–**D**. Likewise, annotated precursor sequences for other neuropeptides included in **B **and** C** are shown in Additional file 13. **B** Alignment of ArKP1.1, ArKP1.2, and KP1-type neuropeptides from other echinoderms (blue) with chordate kisspeptin-type neuropeptides (yellow). **C** Alignment ArKP2.1, ArKP2.2, and KP2-type neuropeptides from other echinoderms (blue) with chordate kisspeptin-type neuropeptides (yellow). **D** Alignment of L-type SALMFamide precursor-derived peptides from *A. rubens* (ArS1.1, ArS1.3, ArS1.4, and S1.7) and other echinoderms (blue) with chordate kisspeptin-type neuropeptides (yellow). **E** Alignment of F-type SALMFamide precursor-derived peptides from *A. rubens* (ArS2.2, ArS2.3, and S2.8) and other echinoderms (blue) with chordate kisspeptin-type neuropeptides (yellow). Conserved residues are highlighted in black or gray. Abbreviations: Ar, *Asterias rubens*; Sp, *Strongylocentrotus purpuratus*; Aj, *Apostichopus japonicus*, Hs, *Homo sapiens*, Lo, *Lepisosteus oculatus*, Bf, *Branchiostoma floridae*. The accession numbers for the precursor sequences used for this figure are in Additional file 13

Having identified a family of kisspeptin-type receptors in *A. rubens* and other echinoderms, we sought to identify the neuropeptides that act as ligands for these receptors. We have reported previously the identification of a precursor protein in *A. rubens* (ArKPP or ArKPP1) that comprises two kisspeptin-like peptides [31], which we refer to here as ArKP1.1 and ArKP1.2 (Fig. 3A; Additional file 12). Furthermore, orthologs of ArKPP1 have also been identified in other echinoderms [32, 53, 54] (Additional file 13). In Fig. 3B, the sequences of ArKP1.1 and ArKP1.2 and related peptides from other echinoderms are aligned with the sequences of chordate kisspeptins, revealing that a C-terminal Leu-*X*-Phe-NH_2_ motif and one or two asparagine (N) residues in the core region are shared characteristics in at least one of the peptides in each species. However, our identification of eleven kisspeptin-type receptors in *A. rubens* (ArKPR1-11; see above) suggested that there may be other neuropeptides that act as ligands for one or more of these receptors. Furthermore, preliminary experiments revealed that ArKP1.1 and ArKP1.2 do not act as ligands for all eleven of the *A. rubens* kisspeptin-type receptors (Additional file 14). Therefore, we examined the sequences of known neuropeptide precursors in *A. rubens* and other echinoderms to identify other proteins comprising neuropeptides that share sequence similarity with vertebrate kisspeptins.

We identified in *A. rubens* the precursor of two neuropeptides that were previously reported as tachykinin-like neuropeptides [31]. Homologs of this precursor have been identified in other echinoderms and these include proteins that have been annotated as precursors of cholecystokinin-like neuropeptides [32, 55], highlighting uncertainty regarding their relationship with neuropeptides in other phyla. There are two tachykinin-type receptors in *A. rubens* [37] and, importantly, here we show that a putative tachykinin-like neuropeptide in *A. rubens* [31] does not act as a ligand for these receptors (Additional file 15). Thus, neuropeptides that act as ligands for tachykinin-type receptors remain to be discovered in echinoderms. However, analysis of the sequences of the neuropeptides previously identified as tachykinin-like or cholecystokinin-like peptides in echinoderms revealed that they also share sequence similarity with chordate kisspeptins (Fig. 3A, C). Accordingly, we now refer to these peptides in *A. rubens* as ArKP2.1 and ArKP2.2 and their precursor protein is now referred to as ArKPP2 (Fig. 3A, C). Equivalent nomenclature is used for orthologous neuropeptides/precursors in other echinoderms (Fig. 3C; Additional file 13). As shown in Fig. 3C, ArKP2.1, ArKP2.2, and related peptides in other echinoderms have a C-terminal Gly-X-Phe-NH_2_ or Gly-X-X-Phe-NH_2_ motif in common with vertebrate kisspeptins. Furthermore, the presence of an aromatic amino acid (Phe, Tyr, or Trp) in the core of the peptide sequences is a characteristic of most of the echinoderm peptides and vertebrate kisspeptins. On this basis, we identified ArKP2.1 and ArKP2.2 as candidate ligands for kisspeptin-type receptors in *A. rubens*.

We also identified precursors of a family of echinoderm neuropeptides known as SALMFamides [31, 34, 35] as candidate ligands for kisspeptin-type receptors. The prototypes for this neuropeptide family were first discovered in *A. rubens* and in the closely related species *Asterias forbesi* in 1991 and were named SALMFamide-1 (S1) and SALMFamide-2 (S2) [34, 56]. S1 is an octapeptide (Gly-Phe-Asn-Ser-Ala-Leu-Met-Phe-NH_2_ or GFNSALMFamide) and S2 is a dodecapeptide (Ser-Gly-Pro-Tyr-Ser-Phe-Asn-Ser-Gly-Leu-Thr-Phe-NH_2_ or SGPYSFNSGLTFamide). Furthermore, the sequences of SALMFamide precursors have been determined in *A. rubens* (Fig. 3A) and other echinoderms (Additional file 13), revealing the existence of two precursor types [57].

Firstly, there are SALMFamide precursors that largely or exclusively comprise neuropeptides with a C-terminal Leu-*X*-Phe-NH_2_ motif (where *X* is a variable), which are referred to as L-type SALMFamide precursors. For example, the L-type SALMFamide precursor in *A. rubens* (ArL-SALMFaP) comprises S1 and six related neuropeptides and here we refer to these peptides as ArS1.1-ArS1.7 in accordance with their position in the precursor (Fig. 3A). Therefore, because S1 is the fourth neuropeptide in the precursor, we also refer to S1 as ArS1.4.

Secondly, there are SALMFamide precursors that largely or exclusively comprise neuropeptides with a C-terminal Phe-*X*-Phe-NH_2_ motif (where *X* is variable), which are referred to as F-type SALMFamide precursors [31, 32, 53, 55, 57–59]. For example, the F-type SALMFamide precursor in *A. rubens* (ArF-SALMFaP) comprises seven peptides with an Phe-*X*-Phe-NH_2_ motif as well as S2, which is atypical because it has a Leu-*X*-Phe-NH_2_ motif. Here, we refer to the neuropeptides derived from this precursor as ArS2.1-ArS2.8 in accordance with their position in the precursor (Fig. 3A). Therefore, because S2 is the third neuropeptide in the precursor, we also refer to S2 as ArS2.3.

Alignment of neuropeptides derived from echinoderm L-type SALMFamide and F-type SALMFamide precursors with chordate kisspeptins revealed sequence similarity (Fig. 3D, E, respectively) comparable to that observed with neuropeptides derived from the echinoderm KPP1 and KPP2 precursors (Fig. 3B, C). Thus, a C-terminal Gly-Leu-*X*-Phe-NH_2_ motif is a characteristic that several echinoderm SALMFamides (e.g., ArS1.3, ArS1.7, ArS2.3) have in common with vertebrate kisspeptins. Furthermore, an Asn-Ser or Asn-Thr motif in the core of the sequences is also a characteristic shared among some of the peptides (Fig. 3D, E). Based on these observations and the fact that SALMFamide receptors have hitherto not been identified in echinoderms, we hypothesized that SALMFamides may be ligands for kisspeptin-type receptors in echinoderms.

### Structure and chromosomal location of genes encoding precursors of candidate neuropeptide ligands for kisspeptin-type receptors in *A. rubens*

**Fig. 4:**
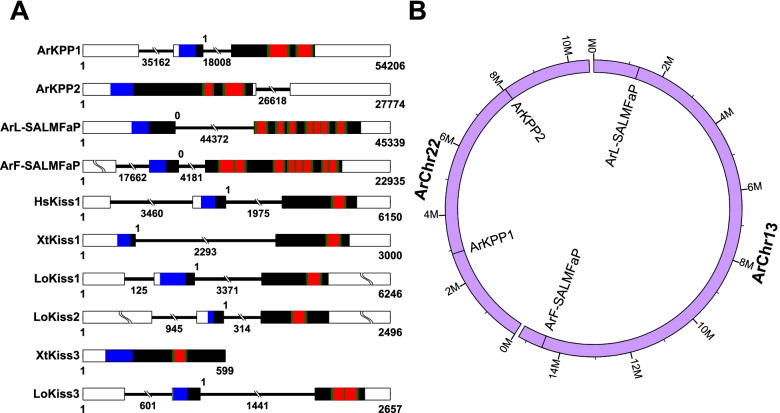
Structure and chromosomal location of genes encoding candidate ligands for *A. rubens* kisspeptin-type receptors. **A** Comparison of the exon/intron structure of genes encoding precursors of candidate ligands for kisspeptin-type receptors in *A. rubens* (Ar) and genes encoding kisspeptin-type precursors in *Homo sapiens* (Hs), *Xenopus tropicalis* (Xt), and *Lepisosteus oculatus* (Lo). Exons are shown as rectangles, with non-coding regions white and protein-coding regions black or colored (regions encoding the N-terminal signal peptide, neuropeptides and predicted monobasic/dibasic cleavage sites are shown in blue, red, and green, respectively). Introns are shown as lines, with length underneath. The presence of a phase 1 intron interrupting the coding sequence between exons encoding the N-terminal signal peptide and one or more neuropeptides is a feature that is conserved between the *A. rubens* KPP1 gene and vertebrate kisspeptin precursor genes, providing evidence of orthology, whereas ArKPP2 is encoded by a single exon. The presence of an intron that interrupts the coding sequence between an exon encoding the N-terminal signal peptide and an exon encoding multiple neuropeptides is also a feature of the two *A. rubens* SALMFamide precursor genes, but this is a phase 0 intron. The presence and position of introns shown here in *A. rubens* neuropeptide precursor genes are conserved in orthologs in other echinoderm species (see Additional file 17). The accession numbers for the sequences of the precursors shown in this figure are listed in Additional file 17. **B** Diagram showing the chromosomal locations of four genes encoding precursors of candidate ligands for kisspeptin-type receptors in *A. rubens*: ArKPP1, ArKPP2, ArL-SALMFaP, and ArF-SALMFaP. Genes encoding ArKPP1 and ArKPP2 are located on chromosome 22 and genes encoding ArL-SALMFaP and ArF-SALMFaP are located on chromosome 13, indicating that these are two pairs of paralogous genes that originated by gene duplication and intrachromosomal translocation

To further investigate relationships between the candidate ligands for kisspeptin-type receptors in echinoderms and chordate kisspeptins, we compared the structure of genes encoding precursors of these neuropeptides (Fig. 4A; Additional file 16; Additional file 17). Furthermore, we also compared the chromosomal locations of genes encoding precursors of candidate neuropeptide ligands for kisspeptin-type receptors in *A. rubens* (Fig. 4B). The gene encoding the *A. rubens* precursor of the kisspeptin-like peptides ArKP1.1 and ArKP1.2 (ArKPP1) comprises two protein-coding exons. The first exon encodes the N-terminal signal peptide, and the second exon encodes the neuropeptides ArKP1.1 and ArKP1.2, with a phase 1 intron separating the two exons. Notably, KPP1 genes in other echinoderms (Additional file 16) and kisspeptin precursor genes in humans and other vertebrates have the same gene structure (Fig. 4A), and therefore, this provides additional evidence that ArKPP1 is an ortholog of vertebrate kisspeptin precursors. Interestingly, and by way of comparison, the gene encoding the *A. rubens* KPP2-type precursor ArKPP2 comprises a single protein-coding exon. However, it is noteworthy that the genes encoding ArKPP1 and ArKPP2 are both located on chromosome 22, which may be reflective of an evolutionary origin from the duplication of a common ancestral gene (i.e., they are paralogs) (Fig. 4B). However, the two genes are separated by over 5 Mb of genomic sequence, and therefore, if they are paralogs that evolved by tandem gene duplication, then subsequent intrachromosomal gene translocation must have occurred.

Similar to ArKPP1 and vertebrate kisspeptin precursors, the L-type SALMFamide and F-type SALMFamide precursors in *A. rubens* are also encoded by genes comprising two exons, with the first exon encoding the N-terminal signal peptide and the second exon encoding several SALMFamide neuropeptides; however, these exons are separated by a phase 0 intron (Fig. 4A; Additional file 16). This difference in the intron phase suggests that SALMFamides are more distantly related to vertebrate kisspeptins than the *A. rubens* kisspeptin-like peptides ArKP1.1 and ArKP1.2. Furthermore, it is noteworthy that the genes encoding the *A. rubens* L-type SALMFamide and F-type SALMFamide precursors are both located on chromosome 13 (Fig. 4B), which may be reflective of an evolutionary origin from the duplication of a common ancestral gene (i.e., they are paralogs). However, the two genes are separated by over 13 Mb of the genomic sequence, and therefore, if they are paralogs that evolved by tandem gene duplication, then subsequent intrachromosomal gene translocation must have occurred.

### Identification of neuropeptides that act as ligands for *A. rubens* kisspeptin-type receptors

Having identified precursors of neuropeptides that are candidate ligands for the *A. rubens* kisspeptin-type receptors ArKPR1-11, we used mass spectrometry to determine the mature structures of these peptides. The structures of fifteen of the nineteen neuropeptides derived from four precursor proteins were determined: (i) ArKP1.1 derived from ArKPP1; (ii) ArKP2.1 and ArKP2.2 derived from the precursor ArKPP2; (iii) ArS1.1, ArS1.2, ArS1.3, ArS1.4, ArS1.6, and ArS1.7 derived from the L-type SALMFamide precursor ArL-SALMFaP; and (iv) ArS2.1, ArS2.2, ArS2.3, ArS2.4, ArS2.6, and ArS2.8 derived from the F-type SALMFamide precursor ArF-SALMFaP (Additional file 18). This revealed that all of these peptides are C-terminally amidated, as expected based on the presence of a glycine residue at the C-terminus of each neuropeptide sequence in the precursor protein. In addition, post-translational conversion of an N-terminal glutamine residue in the precursor protein to a pyroglutamate in the mature neuropeptide was observed for ArKP2.1 and ArS2.1.

Informed by these findings, all nineteen of the peptides predicted or shown to be derived from the four precursors were synthesized to be tested as ligands for ArKPR1-11. ArKP1.1, ArKP1.2, and ArKP2.2 were tested individually as candidate ligands for ArKPR1-11, but it was not possible to test ArKP2.1 because it was found to be insoluble in aqueous media. Peptides derived from the L-type and F-type SALMFamide precursors were tested as precursor-specific ‘cocktails’: (i) ArS1.1-ArS1.7 and (ii) ArS2.1-ArS2.8, respectively, which provided an efficient method for an initial screen of many neuropeptides (15 in total) as candidate ligands for a large number of receptors (11 in total).

**Fig. 5:**
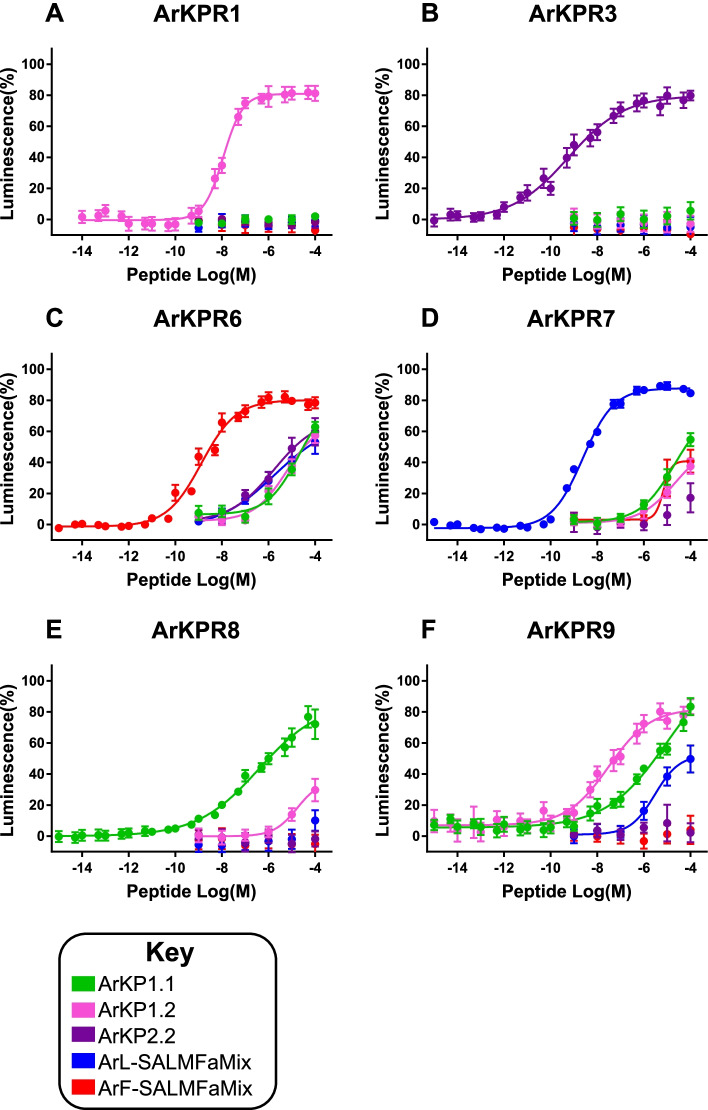
Identification of neuropeptides that act as ligands for *A. rubens* kisspeptin-type receptors. **A** ArKPR1 is only activated by ArKP1.2 (EC_50_ = 1.16 × 10^−8^ M). **B** ArKPR3 is only activated by ArKP2.2 (EC_50_ = 5.80 × 10^−10^ M). **C** ArKPR6 is activated by multiple neuropeptides at high concentrations (> 1 µM) but is only activated by a ‘cocktail’ of neuropeptides derived from the F-type SALMFamide precursor at lower concentrations (EC_50_ = 1.33 × 10^−9^ M). **D** ArKPR7 is activated by multiple neuropeptides at high concentrations (> 1 µM) but is only activated by a ‘cocktail’ of neuropeptides derived from the L-type SALMFamide precursor at lower concentrations (EC_50_ = 2.43 × 10^−9^ M). **E** ArKPR8 is activated by both ArKP1.1 and ArKP1.2 at high concentrations (> 1 µM) but is only activated by ArKP1.1 at lower concentrations (EC_50_ = 3.88 × 10^−7^ M). **F** ArKPR9 is activated by ArKP1.1, ArKP1.2, and a ‘cocktail’ of neuropeptides derived from the L-type SALMFamide precursor, but ArKP1.2 is the most potent ligand for this receptor (EC_50_ = 3.07 × 10^−^.^8^ M). Key: green = ArKP1.1; pink = ArKP1.2; purple = ArKP2.2; blue = ‘cocktail’ of neuropeptides derived from the L-type SALMFamide precursor (ArS1.1–7); red = ‘cocktail’ of neuropeptides derived from the F-type SALMFamide precursor (ArS2.1–8). Each point represents mean values (± S.E.M.) from at least four independent experiments, with each experiment performed in triplicate. Luminescence is expressed as a percentage of the maximal response observed in each experiment. The source data for these experiments is provided in Additional file 15

CHO-cells stably expressing apoaequorin were co-transfected with the promiscuous G-protein G_α_16 and one of the eleven *A. rubens* kisspeptin-type receptors. For initial tests, the cells were exposed to the neuropeptides or neuropeptide ‘cocktails’ at a concentration of 10^−5^ M. For the SALMFamide ‘cocktails’ ArS1.1-ArS1.7 and ArS2.1-ArS2.8, the total peptide concentration in each ‘cocktail’ was 10^−5^ M and this was achieved with each constituent peptide being prepared at concentrations of 0.143 × 10^−5^ M and 0.125 × 10^−5^ M, respectively. No responses to peptides tested at 10^−5^ M (Additional file 14) or at a range of concentrations (10^−9^–10^−4^ M; Additional file 19) were observed in cells expressing ArKPR2, ArKPR4, ArKPR5, ArKPR10, and ArKPR11 but the viability of cells in these tests was confirmed by detection of Triton-X100-induced luminescence. However, peptide-induced luminescence was observed in cells expressing ArKPR1, ArKPR3, ArKPR6, ArKPR7, ArKPR8, and ArKPR9 with one or more of the candidate ligands tested at 10^−5^ M (Additional file 14). Therefore, these receptors were characterized in more detail by testing peptides at concentrations ranging from 10^−15^ to 10^−4^ M (Fig. 5; Additional file 15).

ArKPR1 and ArKPR3 were selectively activated by ArKP1.2 (EC_50_ = 1.16 × 10^−8^ M) and ArKP2.2 (EC_50_ = 5.80 × 10^−10^ M), respectively (Fig. 5A,B).

The F-type SALMFamide precursor-derived ‘cocktail’ was found to be the most potent ligand for ArKPR6 (EC_50_ = 1.33 × 10^−9^ M), with the L-type SALMFamide precursor-derived ‘cocktail’ and other peptides having much higher EC_50_ values (2.07 × 10^−5^ M to 6.14 × 10^−6^ M) (Fig. 5C). Furthermore, as one of the eight constituent neuropeptides derived from the F-type SALMFamide precursor, we also tested S2 (ArS2.3) individually as a ligand for ArKPR6. S2 (ArS2.3) acted as a ligand for ArKPR6, with an EC_50_ of 2.93 × 10^−8^ M. Thus, S2 (ArS2.3) was less potent as a ligand for ArKPR6 than the F-type SALMFamide precursor ‘cocktail’ but S2 (ArS2.3) was still 2–3 orders of magnitude more potent than other peptides tested, including the L-type SALMFamide precursor ‘cocktail’. Moreover, and interestingly, the efficacy of S2 (ArS2.3) as a ligand for ArKPR6 was higher than the F-type SALMFamide precursor ‘cocktail’. Thus, the net activity of the F-type SALMFamide precursor ‘cocktail’ as a ligand for ArKPR6 probably reflects a summation of the varying potencies and efficacies of its constituent peptides (Additional files 15 and 20).

As a converse of findings with ArKPR6, the L-type SALMFamide ‘cocktail’ was found to be the most potent ligand for ArKPR7 (EC_50_ = 2.43 × 10^−9^ M), with the F-type SALMFamide ‘cocktail’ and other peptides having much higher EC_50_ values (1.09 × 10^−5^ to 6.97 × 10^−6^ M) (Fig. 5D). Furthermore, as one of the eight constituent neuropeptides derived from the L-type SALMFamide precursor, we also tested S1 (ArS1.4) individually as a ligand for ArKPR7 and found that it acted as a ligand for ArKPR7 with an EC_50_ of 4.79 × 10^−9^ M, which is similar to the EC_50_ of the L-type SALMFamide precursor ‘cocktail’. Thus, the activity of S1 (ArS1.4) as a ligand for ArKPR7 appears to be representative of the activity of the L-type SALMFamide precursor ‘cocktail’ as a whole (Additional files 15 and 20).

ArKP1.1 was the most potent ligand for ArKPR8 (EC_50_ = 3.88 × 10^−7^ M), with other peptides only evoking responses at 10^−5^ M or 10^−4^ M (Fig. 5E). ArKP1.2 was the most potent ligand for ArKPR9 (EC_50_ = 3.07 × 10^−8^ M), but cells expressing ArKPR9 were also responsive to ArKP1.1 (EC_50_ = 1.00 × 10^−5^ M) and the L-type SALMFamide ‘cocktail’ (EC_50_ = 2.95 × 10^−6^ M) (Fig. 5F).

Collectively, these findings indicate that (i) ArKPR1, ArKPR8, and ArKPR9 are receptors for the ArKPP1-derived neuropeptides ArKP1.1 and/or ArKP1.2; (ii) ArKPR3 is a receptor for the ArKPP2-derived neuropeptide ArKP2.2; (iii) ArKPR6 is a receptor for F-type SALMFamide precursor-derived neuropeptides; and (iv) ArKPR7 is a receptor for L-type SALMFamide precursor-derived neuropeptides.

## Discussion

### Discovery of ligands for an expanded family of kisspeptin-type receptors in echinoderms

Here, we report the discovery of an expanded family of eleven G-protein-coupled receptors (GPCRs) in the starfish *A. rubens*, which based on phylogenetic analysis and comparison of gene structure are identified as homologs of vertebrate kisspeptin-type receptors. Therefore, we named these receptors *A. rubens* kisspeptin-type receptors ArKPR1-11. Homologs of ArKPR1-11 were also identified in other echinoderms. Previously, we reported the identification of a protein precursor of two kisspeptin-like peptides in *A. rubens* (ArKP1.1, ArKP1.2) and homologs have subsequently been identified in other echinoderms [31, 32, 53, 55, 59]. Therefore, we tested ArKP1.1 and ArKP1.2 as candidate ligands for ArKPR1-11, which revealed that ArKP1.1 and/or ArKP1.2 act as ligands for ArKPR1, ArKPR6, ArKPR7, ArKPR8, and ArKPR9, but with variable selectivity/potency. Thus, ArKPR1 is only activated by ArKP1.2, whereas ArKPR8 and ArKPR9 are activated by both peptides but preferentially by ArKP1.1 and ArKP1.2, respectively. Furthermore, ArKP1.1 and ArKP1.2 only acted as ligands for ArKPR6 and ArKPR7 at high concentrations (> 1 µM). Based on these findings, we concluded that ArKP1.1 and/or ArKP1.2 are ligands for ArKPR1, ArKPR8, and ArKPR9 and other peptides may act as ligands for the other kisspeptin-type receptors in *A. rubens*.

To identify other potential ligands for kisspeptin-type receptors in *A. rubens*, we analyzed known neuropeptide precursor sequences in this species [31] for the presence of peptides that share sequence similarity with ArKP1.1, ArKP1.2, and/or vertebrate kisspeptins. A protein previously annotated as a tachykinin-type precursor [31] was identified as the precursor of two kisspeptin-like peptides — ArKP2.1 and ArKP2.2. However, it was not possible to test ArKP2.1 because of its low solubility in water, but ArKP2.2 was successfully tested. Notably, we discovered that ArKP2.2 does not act as a ligand for two *A. rubens* tachykinin-type receptors (ArTKR1, ArTKR2; [37]; Additional file 15), demonstrating that our original annotation of this peptide as a tachykinin-type neuropeptide was incorrect and highlighting the difficulty in determining neuropeptide relationships based solely on sequence similarity. Furthermore, and importantly, we found that ArKP2.2 acts as a potent ligand for the *A. rubens* kisspeptin-type receptor ArKPR3. Interestingly, however, ArKP2.2 did not act as a ligand for ArKPR2 and ArKPR4, which are closely related to ArKPR3. We speculate, therefore, that ArKP2.1 may act as a ligand for ArKPR2 and/or ArKPR4 physiologically, but we were unable to test this hypothesis because of the insolubility of ArKP2.1. Receptors closely related to ArKPR2-4 and KPP2-type precursor proteins are also present in other echinoderms. Therefore, we conclude that a novel kisspeptin-related neuropeptide signaling system comprising ArKPP2-derived neuropeptides and ArKPR2-4-type receptors has been discovered in *A. rubens*. It is noteworthy that neuropeptides derived from orthologs of ArKPP2 in the sea urchin *S. purpuratus* and the sea cucumber *A. japonicus* have been annotated previously as cholecystokinin-related peptides [55, 60], which further highlights the difficulty in determining neuropeptide relationships based solely on sequence similarity. Furthermore, building upon a previous study [33], our findings from *A. rubens* provide a basis for testing neuropeptides derived from the KPP2-type precursor AjKPP2 as ligands for kisspeptin-type receptors in *A. japonicus.*

Other *A. rubens* proteins found to comprise neuropeptides that share sequence similarity with kisspeptins were two SALMFamide-type precursors. SALMFamides were the first neuropeptides to be identified in echinoderms over 30 years ago, with two related peptides known as SALMFamide-1 (S1; GFNSALMF-NH_2_) and SALMFamide-2 (S2; SGPYSFNSGLTF-NH_2_) both being isolated from the starfish species *A. rubens* and *Asterias forbesi* [34, 56]. Subsequent studies have revealed that SALMFamide-type neuropeptides are present in other echinoderms and analysis of transcriptome/genome sequence data has revealed that eleutherozoan echinoderms have two SALMFamide genes: a gene encoding an L-type SALMFamide precursor that largely comprises neuropeptides with a C-terminal Leu-*X*-Phe-NH_2_ motif (where *X* is a variable) and a gene encoding an F-type SALMFamide precursor that largely comprises neuropeptides with a C-terminal Phe-*X*-Phe-NH_2_ motif [57]. Thus, in *A. rubens*, the L-type SALMFamide precursor comprises S1 and six other neuropeptides with a Leu-*X*-Phe-NH_2_ motif, which we refer to here as ArS1.1-ArS1.7 based on their position in the precursor protein, and the F-type SALMFamide precursor comprises S2 and seven neuropeptides with a Phe-*X*-Phe-NH_2_ motif, which we refer to here as ArS2.1-ArS2.8 based on their position in the precursor protein [31]. Furthermore, here we tested precursor-specific ‘cocktails’ of these peptides (S1.1-S1.7 or S2.1-S2.8) as candidate ligands for the *A. rubens* kisspeptin-type receptors ArKPR1-11. Importantly, we discovered that the ArS1.1-ArS1.7 ‘cocktail’ and S1 (ArS1.4) act as potent ligands for ArKPR7 and the ArS2.1-ArS2.8 ‘cocktail’ and S2 (ArS2.3) act as potent ligands for ArKPR6. ArKPR7 was also activated by ArKP1.1, ArKP1.2, ArKP2.2, and the ArS1.1-ArS1.7 ‘cocktail’ but only at high concentrations (> 1 µM), and therefore, we conclude that ArKPR7 acts physiologically as a receptor for S2 and other F-type SALMFamide precursor-derived peptides in *A. rubens*. Likewise, ArKPR6 was also activated by ArKP1.1, ArKP1.2, ArKP2.2, and the ArS2.1-ArS1.8 ‘cocktail’ but only at high concentrations (> 1 µM), and therefore, we conclude that ArKPR6 acts physiologically as a receptor for S1 and other L-type SALMFamide precursor-derived peptides in *A. rubens*. The identification of ArKPR6 and ArKPR7 as receptors for SALMFamide-type neuropeptides in echinoderms represents a significant milestone for research on neuropeptide signaling. Thus, neuropeptides discovered in invertebrates in 1991 [34, 56], 10 years before the discovery of the kisspeptin signaling system in mammals in 2001 [1], are in fact ligands for kisspeptin-type receptors, changing our perspective on the history of research on kisspeptin-type signaling.

The discovery of an expanded family of kisspeptin-type receptors in echinoderms and the identification of novel neuropeptide ligands for six of these receptors in the starfish *A. rubens* are a major advance in our knowledge of neuropeptide signaling systems in echinoderms. However, ligands remain to be identified for five of the eleven kisspeptin-type receptors in *A. rubens*. As discussed above, we speculate that ArKPR2 and/or ArKPR4 may be receptors for the peptide ArKP2.1, which we were unable to test effectively because of its insolubility. Ligands for ArKPR5 remain to be identified, although weak activation of this receptor was observed with several of the *A. rubens* kisspeptin-like peptides tested here but only at very high concentrations. It is noteworthy from our phylogenetic analysis that ArKPR5 is positioned in the same clade as the SALMFamide receptors ArKPR6 and ArKPR7. Therefore, undiscovered SALMFamide-related neuropeptides may remain to be identified that act as ligands for ArKPR5. Two other *A. rubens* kisspeptin-type receptors for which ligands were not identified are ArKPR10 and ArKPR11. It is noteworthy from our phylogenetic analysis that these receptors are positioned in a branch of the tree in Fig. 1 (branch 3.3) where there are very long branch lengths, and therefore, these receptors are much more divergent with respect to vertebrate kisspeptin-type receptors by comparison with the other *A. rubens* kisspeptin-type receptors ArKPR1-9. Interestingly, a kisspeptin-type receptor in the sea cucumber *A. japonicus* named AjKissR1, which has been shown to be activated by a peptide derived from a KPP1-type precursor [33], is positioned in the same branch of clade 3 (branch 3.3) as ArKPR10 and ArKPR11, and therefore, this receptor is also referred to as AjapKPR10_11 in Fig. 1. However, additional phylogenetic analyses (Additional files 4, 5, and 6) and CLANS analysis (Additional file 8) indicate that AjKissR1 is not closely related to ArKPR10 or ArKPR11.

The discovery of neuropeptides that act as ligands for kisspeptin-type receptors in *A. rubens* also provides a basis for the investigation of their physiological roles. Currently, nothing is known about the physiological roles of the kisspeptin-type neuropeptides ArKP1.1, ArKP1.2, ArKP2.1, and ArKP2.2 in *A. rubens*, and therefore, this will be an objective for future research. However, the physiological roles of orthologs of ArKP1.1 and ArKP1.2 have been investigated recently in the sea cucumber *A. japonicus*, and interestingly, in accordance with kisspeptin function in vertebrates, this has revealed evidence of roles in the regulation of reproductive physiology in these animals [33]. Furthermore, there is an extensive body of literature reporting the expression and actions of SALMFamide-type neuropeptides in starfish and in other echinoderms. A detailed review of these studies was published in 2014 [35], and therefore, here we will only summarize some key findings. The expression of the genes encoding L-type and F-type SALMFamide precursors has been examined in larvae of *A. rubens* using mRNA in situ hybridization methods [61] and the distribution of S1 and S2 derived from these precursors, respectively, has been examined in larval and adult *A. rubens* using immunohistochemical methods [62–64], revealing widespread patterns of expression indicative of roles in the regulation of a variety of physiological processes. Furthermore, in vitro and in vivo pharmacological studies have revealed that S1 and S2 act as muscle relaxants in *A. rubens* [65, 66] and likewise SALMFamide-type neuropeptides also act as muscle relaxants in other echinoderms [67]. More specifically, S2 causes stomach eversion in *A. rubens* [66], indicating that it is one of several neuropeptides involved in the regulation of the unusual feeding behavior of starfish where the stomach is everted out of the mouth and over the digestible soft tissue of prey (e.g., mussels) [43]. Furthermore, S1 causes inhibition of neural release of a relaxin-like gonadotropic neuropeptide in starfish [68]. Thus, SALMFamides are involved in the regulation of feeding behavior and reproductive physiology in starfish, observations that can now be interpreted in a comparative context with respect to the physiological roles of kisspeptins in vertebrates [9, 13, 15]. Further insights into the comparative and evolutionary physiology of kisspeptin-type signaling will be gained as we learn more about the functions of the different kisspeptin-type neuropeptides that have been identified here in echinoderms.

### Discovery of ligands for the expanded family of kisspeptin-type receptors in *A. rubens* provides new insights into the evolution of neuropeptide signaling in the Bilateria

Our discovery of neuropeptides that act as ligands for the expanded family of kisspeptin-type receptors in the starfish *A. rubens* not only provides insights into neuropeptide diversity in echinoderms but also provides broader and novel perspectives on the evolution of kisspeptin-type neuropeptide signaling in bilaterian animals. Evolutionary interpretations are based on our phylogenetic analysis, which revealed that the echinoderm kisspeptin-type receptors are positioned in three distinct clades, as discussed below.

Clade 1 includes the *A. rubens* receptor ArKPR3, which is activated by the peptide ArKP2.2, closely related receptors in *A. rubens* (ArKPR2, ArKPR4) and in other echinoderms, and chordate kisspeptin-type receptors. Therefore, this suggests that neuropeptides derived from KPP2-type precursors and their cognate receptor(s) constitute a kisspeptin-type signaling system in echinoderms that is closely related to chordate kisspeptin-type signaling.

Clade 2 includes ArKPR1, which is activated by the peptide ArKP1.2, a closely related receptor in the feather star *A. japonica* and lophotrochozoan kisspeptin-type receptors. Unfortunately, the neuropeptides that act as ligands for kisspeptin-type receptors in lophotrochozoans have yet to be identified. Therefore, our discovery of a ligand (ArKP1.2) for the kisspeptin-type receptor in starfish that is most closely related to lophotrochozoan kisspeptin-type receptors is noteworthy because it may provide a basis for the discovery of orthologous peptides in lophotrochozoans.

Clade 3 has the largest representation of echinoderm kisspeptin-type receptors and comprises three branches. Branch 3.1 includes the *A. rubens* kisspeptin-type receptors ArKPR8 and ArKPR9, both of which are activated by the *A. rubens* kisspeptin-like peptides ArKP1.1 and ArKP1.2 but with differing potencies. Branch 3.2 includes the *A. rubens* kisspeptin-type receptors ArKPR6 and ArKPR7, which are receptors for neuropeptides derived from the F-type and L-type SALMFamide precursors, respectively. Lastly, branch 3.3 includes the *A. rubens* kisspeptin-type receptors ArKPR10 and ArKPR11, for which ligands have yet to be identified.

The diversity and complexity of kisspeptin-type signaling systems in echinoderms present a challenge for interpretation, and it is clear from our phylogenetic analysis that there is a much greater variety of kisspeptin-type signaling systems in echinoderms than in vertebrates or in any other invertebrate taxa that have been investigated experimentally thus far. But is this diversity of kisspeptin-type signaling systems a characteristic that has uniquely evolved in echinoderms or is it reflective of the diversity that may also be applicable to other phyla? Or rephrasing this question from an evolutionary perspective, when during animal evolution did the diversity of kisspeptin-type signaling systems that we find in extant starfish, and by inference in other extant echinoderms, originate? Interestingly, an expanded family of kisspeptin-type receptors has also been identified in the cephalochordate *B. floridae* [16, 17]. However, our phylogenetic analysis indicates that this is largely a consequence of multiple gene duplications that have occurred within the cephalochordate lineage. Our observation that ArKPR1 and closely related receptors in other echinoderms are positioned in a clade (clade 2) that also contains hemichordate and lophotrochozoan kisspeptin-type receptors indicates that this kisspeptin-type signaling system originated in a common ancestor of all bilaterian animals, but was subsequently lost in some lineages, including chordates, nematodes, and arthropods. In contrast, the clade 1 kisspeptin-type receptors only comprise receptors from echinoderms, hemichordates, and chordates, and therefore, the evolutionary origin of this clade can be traced to the common ancestor of the deuterostomes, although we cannot rule out an earlier urbilaterian origin with subsequent loss in protostomes. The clade 3 kisspeptin-type receptors only have representation in echinoderms and hemichordates, and therefore, excluding a non-parsimonious scenario of multiple losses of these receptors in other phyla, we speculate these receptors may have originated in a common ancestor of the Ambulacraria. In this context, it is interesting that clade 3 includes *A. rubens* receptors that are activated by neuropeptides or ‘cocktails’ of neuropeptides derived from three different precursor proteins, ArKPP1 and the two SALMFamide-type neuropeptide precursors. It appears, therefore, that there has been a remarkable increase in the diversity of kisspeptin-type neuropeptides and receptors in echinoderms, and probably also in hemichordates. Accordingly, from a comparative perspective, it would be of interest in the future to identify neuropeptides that act as ligands for kisspeptin-type receptors in hemichordates (e.g., *S. kowalevskii*) as this may provide further insights into the evolution of kisspeptin-type signaling in the ambulacrarian branch of the animal kingdom.

## Conclusions

Our discovery of multiple kisspeptin-type signaling systems in the starfish *A. rubens*, which include SALMFamide neuropeptides, has changed our perspective on the history of research on kisspeptin signaling and provided important new insights into the evolution of neuropeptide diversity in the Bilateria. Furthermore, the discovery of multiple kisspeptin-type signaling systems in *A. rubens* and other echinoderms provides the foundations for a new era of experimental studies on the comparative and evolutionary physiology of kisspeptin-type signaling in invertebrates.

## Methods

### Identification of kisspeptin-type receptors in the starfish *A. rubens* and phylogenetic analysis of their relationships with kisspeptin-type receptors in other taxa

To identify kisspeptin-type receptors in the starfish *A. rubens*, neural transcriptome sequence data [31, 36] and *A. rubens* genome sequence data [44] were analyzed by BLAST using the sequence of the human kisspeptin receptor (NP_115940.2) and the sequences of kisspeptin-type receptors (XP_793873.2, XP_796286.1) previously identified the sea urchin *Strongylocentrotus purpuratus* [17] as queries. Eleven transcripts/genes encoding candidate *A. rubens* kisspeptin-type receptors were identified, and the sequences of the proteins encoded by these transcripts/genes were determined using the ExPASy translate tool (http://web.expasy.org/translate/) and named ArKPR1 to ArKPR11. Homologs of the *A. rubens* kisspeptin-type receptors were identified by analysis of transcriptome/genome/proteome sequence data from other echinoderm species, with thirteen, eight, seven, and seven kisspeptin-type receptors being identified in the starfish *Acanthaster planci*, the sea urchin *S. purpuratus*, the sea cucumber *Apostichopus japonicus*, and the crinoid *Annessia japonica,* respectively.

To facilitate the analysis of relationships between kisspeptin-type receptors in echinoderms and in other phyla/sub-phyla, the sequences of the human kisspeptin receptor (NP_115940.2) and the sequences of kisspeptin-type receptors (XP_003727259.1, XP_011680162.1, XP_784787.2, XP_01669579.1, XP_011669580.1, XP_796690.1, XP_796286.1, XP_793873.1, XP_787561.2) previously identified the sea urchin *Strongylocentrotus purpuratus* were submitted as BLAST queries against the proteomes of thirty species (*Homo sapiens*, *Mus musculus*, *Python bivittatus*, *Xenopus tropicalis*, *Lepisosteus oculatus*, *Latimeria chalumnae*, *Branchiostoma floridae*, *Branchiostoma belcheri*, *Ciona intestinalis*, *Asterias rubens*, *Acanthaster planci*,* Anneissia japonica*,* Apostichopus japonicus*,* S. purpuratus*, *Saccoglossus kowalevskii*,* Priapulus caudatus*,* Caenorhabditis elegans*,* Prostionchus pacificus*,* Steinernema carpocapsae*,* Euperipatoides rowelli*,* Hypsibius exemplaris*,* Ramazzottius varieornatus*,* Apis mellifera*,* Daphnia magna*,* Drosophila melanogaster*,* Clonorchis sinensis*,* Helobdella robusta*,* Platynereis dumerilii*,* Aplysia californica*,* Crassostrea gigas*,* Lingula anatina*,* Bugula neritina*,* Adineta steineri*,* Rotaria socialis*, *Rotaria sordida*) from sixteen bilaterian sub-phyla/phyla (Vertebrata, Cephalochordata, Urochordata, Echinodermata, Hemichordata, Priapulida, Nematoda, Onychophora, Tardigrada, Arthropoda, Platyhelminthes, Annelida, Mollusca, Brachiopoda, Bryozoa, Rotifera), which were downloaded from NCBI. Proteomes were cleaned to remove redundant sequences using cd-hit version 4.8.1 [69] with a sequence identity threshold of 0.9. BLAST analysis with reciprocal BLAST was performed using TBtools (Toolbox for biologists) v1.098746 [70], with an *e*-value of 1e − 20 and restricted to the first 15 hits.

To investigate the relationships of the echinoderm kisspeptin-type receptors with kisspeptin-type receptors in other taxa (including chordates, hemichordates, annelids, and mollusks; see Additional file 3 for a list of sequences), a phylogenetic analysis was performed using the maximum-likelihood method [51, 71]. Receptor sequences were aligned using MAFFT v7 E-INS-I (iterative). The sequences were trimmed using TrimAI with the gappy-out option [72]. The maximum-likelihood tree was built using PhyML version 3.0 (LG + G4 amino-acid substitution model, Branch Support bootstrap 1000 replicates) [50]. To specifically enable the investigation of relationships with an expanded family of kisspeptin-type receptors in cephalochordate *B. floridae*, an extended maximum-likelihood tree (LG + G4 amino-acid substitution models, Branch Support bootstrap 1000 replicates) was generated using IQ-tree version 1.6.12 [73, 74] and using SH-aLRT and ultrafast bootstrap (UFBoot) analysis with nearest neighbor interchange (NNI) correction tests [75, 76]. Informed by the results of these analyses, the phylogenetic position of echinoderm kisspeptin-type receptors with long branches in clade 3.3 of Fig. 1 was investigated in more detail. For this analysis, a reduced list of sequences was analyzed but with representative sequences for all clades in Fig. 1. A maximum-likelihood tree (WAG + G4 amino-acid substitution model, Branch Support bootstrap 1000 replicates) was generated using IQ-tree version 1.6.12, as described above. A Bayesian tree was constructed using MrBayes version 3.2.7a (WAG + G4 amino-acid substitution model, 120,000 samples every 1000 generations, two independent MCMC runs that used eight parallel chains composed of six heated and two cold chains at 0.1 of burnin temperature and burnin fraction of 25%) [77, 78].

A cluster-based analysis of the receptor sequences was performed using CLANS [79] with the scoring matrix BLOSUM62 and linkage clustering performed with an *e*-value of 1e^−40^ to identify coherent clusters. Two sets of sequences were analyzed using CLANS. Firstly, a large dataset containing sequences identified by the BLAST analysis of bilaterian proteomes (Additional file 2), which included kisspeptin-type receptors, the closely related galanin/allatostatin-A-type receptors, and other neuropeptide receptor types: allatotropin/orexin, neuropeptide Y, tachykinin, somatostatin/allatostatin-C, CCHamide/gastrin-releasing peptide. Secondly, a smaller dataset (Additional file 3) comprising the receptors in the phylogenetic tree is shown in Fig. 1.

### Comparative analysis of the structure of genes encoding kisspeptin-type receptors in echinoderms and other taxa

Gene structure was determined by comparing the known or predicted cDNA sequence for each receptor with genomic sequence data in the following species: the starfish *A. rubens*, the starfish *A. planci*, the sea cucumber *A. japonicus*, the sea urchin *S. purpuratus*, the spotted gar (*Lepisosteus oculatus*), the western-clawed frog (*Xenopus tropicalis*), *Homo sapiens*, and the mollusk *Crassostrea gigas* (with sequence data obtained from GenBank; https://www.ncbi.nlm.nih.gov/genbank/). Analysis of gene structure was performed using the NCBI ProSplign tool [80] and diagrams of gene structure were generated using IBS: Illustrator for Biological Sequences v1.0.3 [81]. A list of scaffolds, gene IDs, proteins, and sequence data used for this analysis is provided in Additional file 9.

### Analysis of the chromosomal location of genes encoding kisspeptin-type receptors in *A. rubens*

Using the information on the localization of the ArKPR1-11 genes obtained from the NCBI ProSplign tool [80], and the size of the corresponding chromosomes of *A. rubens* derived from genome sequence data [44], mapping of the ArKPR1-11 genes was performed using the software circus-0.69–9 [82]. A list of scaffolds, gene IDs, proteins, and sequence data used for this analysis is provided in Additional file 9. The absence or presence of genes in between genes encoding kisspeptin-type receptor genes was investigated by using the gene table from the NCBI data tables for *A. rubens* chromosomes 10 and 15; this was done to investigate the occurrence of tandem gene duplication giving rise to clusters of genes encoding phylogenetically closely related kisspeptin-type receptors.

### Identification of transcripts encoding precursors of kisspeptin-like peptides in the starfish *A. rubens* and determination of the mature structures of these peptides using mass spectrometry

To identify candidate ligands for kisspeptin-type receptors in *A. rubens*, the sequences of neuropeptide precursors previously identified in this species [31] were analyzed. Four transcripts encoding precursors of neuropeptides that share sequence similarity with vertebrate kisspeptins were identified: (1) the *A. rubens* kisspeptin-type precursor (ArKPP1; GenBank KT601705.1), the *A. rubens* L-type SALMFamide precursor (Ar-L-SALMFaP; GenBank KT601732.1), the *A. rubens* F-type SALMFamide precursor (Ar-F-SALMFaP; GenBank KP330476.1), and the *A. rubens* precursor of two tachykinin-like peptides that have a C-terminal GxFamide motif (ArTKP or ArKPP2; GenBank KT601707) [31]. To determine the structure of the mature peptides derived from these precursors, a methanol/acetic acid/water (90:9:1, v/v/v) extract of radial nerve cords from *A. rubens* was analyzed by mass spectrometry, employing methods that have been described in detail previously [38, 47] for other *A. rubens* neuropeptides.

### Sequence alignment of echinoderm kisspeptin-like peptides and vertebrate kisspeptin-type peptides

The sequences of echinoderm kisspeptin-like peptides were aligned with kisspeptin-type peptides that have been identified in the chordates (see Additional file 12) using MAFFT version 7 (5 iterations, substitution matrix; BLOSUM62) and then manually curated. Highlighting of the conserved residues was performed using BOXSHADE32 (www.ch.embnet.org/software/BOX_form.html) with 40% conservation as the minimum for highlighting.

### Comparative analysis of the structure of genes encoding precursors of kisspeptin-like peptides in echinoderms and kisspeptin-type peptides in vertebrates

The methods employed were the same as described for comparative analysis of the structure of genes encoding kisspeptin-type receptors (see above). A list of scaffolds, gene IDs, proteins, and sequence data used for this analysis is provided in Additional file 12.

### Analysis of the chromosomal location of genes encoding precursors of candidate ligands for kisspeptin-type receptors in *A. rubens*

The methods employed were the same as described for comparative analysis of the chromosomal location of genes encoding kisspeptin-type receptors in *A. rubens* (see above). A list of scaffolds, gene IDs, proteins, and sequence data used for this analysis is provided in Additional file 12.

### Pharmacological characterization of *A. rubens* kisspeptin-type receptors

To enable testing of neuropeptides as candidate ligands for the *A. rubens* kisspeptin-type receptors, cDNAs encoding ArKPR1-11 were custom synthesized with a 5′ partial Kozak translation initiation sequence (CACC) and incorporated into the eukaryotic expression vector pcDNA 3.1 ( +) (GenScript, Piscataway, NJ, USA). Chinese hamster ovary (CHO)-K1 cells stably expressing the calcium-sensitive aequorin fusion protein G5A [83] were used as an expression system. This cell line was supplied by Prof. Gáspár Jékely (University of Exeter) and tested negative for mycoplasma contamination using a MycoAlert PLUS kit (Lonza, Switzerland). The CHO-K1 cells were cultured and upon reaching 80% confluency were transfected with a plasmid containing an *A. rubens* kisspeptin receptor cDNA (ArKPR1-11) and a plasmid containing a cDNA encoding the promiscuous human G-protein Gα-16 that can couple a wide range of GPCRs to the phospholipase C signaling pathway. Neuropeptides were tested as ligands for each receptor in luminescence-based assays, as described previously [37]. The following *A. rubens* neuropeptides identified as candidate ligands for the eleven kisspeptin-type receptors were synthesized by Peptide Protein Research Ltd (Fareham, UK) with a purity of > 95%, as determined by high-performance liquid chromatography (HPLC), and then tested individually (1–4) or as peptide ‘cocktails’ (5–6): 1. ArKP1.1, 2. ArKP1.2, 3. ArKP2.1, 4. ArKP2.2, 5. a ‘cocktail’ of seven neuropeptides derived from the *A. rubens* L-type SALMFamide precursor (ArS1.1-S1.7) and 6. a ‘cocktail’ of eight neuropeptides derived from the *A. rubens* F-type SALMFamide precursor (ArS2.1-ArS2.8) (see Additional file 18 for the structures of these neuropeptides). ArKP1.1 and ArKP1.2 were dissolved in ultra-distilled water, but due to low solubility in water, ArKPP2.1, ArKPP2.2, the L-type SALMFamide ‘cocktail’ and the F-type SALMFamide ‘cocktail’ were first dissolved in dimethyl sulfoxide (DMSO) and then ultra-distilled water was added to obtain a peptide stock solution at 10^−3^ M in 10% DMSO. To obtain lower peptide concentrations for the receptor assays, the stock solution was diluted in the DMEM/F12 Nutrient Mixture medium (Thermo Fisher Scientific; Cat. No. 11039047) used for culturing CHO-K1 cells. However, because the addition of DMEM/F12 Nutrient Mixture medium to the 10^−3^ M solution of ArKPP2.1 in 10% DMSO caused precipitation, even with higher concentrations of DMSO in the stock solution, it was not possible to test ArKP2.1 as a ligand in the receptor assays. The peptides ArKP1.1, ArKP1.2, ArKP2.2, the L-type SALMFamide ‘cocktail’, and the F-type SALMFamide ‘cocktail’ were tested as ligands for the receptors in microtiter plate assays at concentrations ranging from 1 × 10^−15^ to 10^−4^ M (in triplicate for each concentration). Following the addition of transfected CHO-K1 cells to each test well of the microtiter plate, luminescence was recorded over a 35-s period using a FLUOstar Omega Plate Reader (BMG LABTECH; FLUOstar Omega Series multi-mode microplate reader). Data were integrated over the 35-s measurement period and the mean of the triplicate measurements was calculated. Responses were normalized to the maximum response obtained in each experiment (100% activation) and to luminescence measured with the vehicle media (0% activation). Dose–response curves were fitted with a four-parameter curve and EC_50_ values were calculated from dose–response curves based on at least three measurements from three independent transfections using Prism 6.0 (GraphPad, La Jolla, USA).

## Supplementary Information

**Additional file 1. **Transcript sequences and protein sequences of *Asterias rubens* kisspeptin-type receptors ArKPR1-11.

**Additional file 2. **Results of BLAST analysis of bilaterian proteomes using the sequence of the human kisspeptin receptor (NP_115940.2) and the sequences of kisspeptin-type receptors (XP_003727259.1, XP_011680162.1, XP_784787.2, XP_01669579.1, XP_011669580.1, XP_796690.1, XP_796286.1, XP_793873.1, XP_787561.2) previously identified in the sea urchin *Strongylocentrotus purpuratus* as queries. In each page the results of the BLAST (columns A-L) and reciprocal BLAST against *S. purpuratus *(columns O-Z) and *H. sapiens *(columns AC-AN) for each species are shown, with pages labelled with the name of the species analysed. In column AQ the annotation names from NCBI of the proteins used as queries for the reciprocal BLAST from each species are shown, with receptors annotated as GPR54 or kisspeptin-type receptors highlighted in blue.

**Additional file 3. **Accession numbers of the receptor sequences used for the phylogenetic or CLANS analysis shown in Fig. 1 and in additional files 4, 5, 6 and 8.

**Additional file 4. **Phylogenetic analysis of bilaterian kisspeptin-type receptors, including expanded receptor families in the starfish *A. rubens* (ArubKPR1-11), other echinoderms, and *Branchiostoma floridae*. The phylogenetic tree was constructed using the maximum-likelihood method, LG+G4 amino-acid substitution model, and rooted with galanin/allatostatin-A-type receptors as an outgroup. Boostrap support for each node is stated according to SH-aLRT/UFBoot methods. The scale bar indicates amino acid substitutions per site. Species names are as follows: Apla, *Acanthaster planci*; Ajap, *Apostichopus japonicus*; Arub, *Asterias rubens*, Anjap *Annessia japonica*; Bbel, *Branchiostoma belcheri*; Bflo *B. floridae*; Ctel, *Capitella teleta*; Cgig, *Crassostrea gigas*; Hsap, *Homo sapines*; Lcha, *Latimeria chalumnae*; Locu, *Lepisosteus oculatus*; Lgig, *Lottia gigantea*; Mmus, *Mus musculus*; Pdum, *Platynereis dumerilii*; Pbiv, *Python bivittatus*; Skow, *Saccoglossus kowalevskii*; Spur, *Strongylocentrotus purpuratus*; Xlae *Xenopus *laevis; Xtro, *X. tropicalis*. Accession numbers for the sequences of the receptors included in this tree are listed in additional file 3.

**Additional file 5. **Phylogenetic analysis of bilaterian kisspeptin-type receptors, including expanded receptor families in the starfish *A. rubens* (ArubKPR1-11) and other echinoderms. The phylogenetic tree was constructed using the maximum-likelihood method, WAG+G4 amino-acid substitution model, and rooted with galanin/allatostatin-A-type receptors as an outgroup. Boostrap support for each node is stated according to SH-aLRT/UFBoot methods. The scale bar indicates amino acid substitutions per site. Species names are as follows: Ajap, *Apostichopus japonicus*; Arub, *Asterias rubens*, Anjap *Annessia japonica*; Bflo, *Branchiostoma floridae*; Cgig, *Crassostrea gigas*; Hsap, *Homo sapines*; Locu, *Lepisosteus oculatus*; Pdum, *Platynereis dumerilii*; Skow, *Saccoglossus kowalevskii*; Spur, *Strongylocentrotus purpuratus*; Xtro *Xenopus tropicalis*. Accession numbers for the sequences of the receptors included in this tree are listed in additional file 3.

**Additional file 6. **Phylogenetic analysis of bilaterian kisspeptin-type receptors, including expanded receptor families in the starfish *A. rubens* (ArubKPR1-11) and other echinoderms. The phylogenetic tree was constructed using the Bayesian method, WAG+G4 amino-acid substitution model, and rooted with galanin/allatostatin-A-type receptors as an outgroup. Probabilities for nodes are stated according to SH-aLRT/UFBoot methods. The scale bar indicates amino acid substitutions per site. Species names are as follows: Ajap, *Apostichopus japonicus*; Arub, *Asterias rubens*, Anjap *Annessia japonica*; Bflo, *Branchiostoma floridae*; Cgig, *Crassostrea gigas*; Hsap, *Homo sapines*; Locu, *Lepisosteus oculatus*; Pdum, *Platynereis dumerilii*; Skow, *Saccoglossus kowalevskii*; Spur, *Strongylocentrotus purpuratus*; Xtro *Xenopus tropicalis*. Accession numbers for the sequences of the receptors included in this tree are listed in additional file 3.

**Additional file 7. **CLuster Analysis of Sequences (CLANS) of receptors identified by BLAST analysis of bilaterian proteomes using human and *S. purpuratus* kisspeptin-type receptors as queries. BLOSUM62 cluster map shows kisspeptin-type receptors, the closely related galanin/allatostatin-A-type receptors, and other neuropeptide receptor families: allatotropin/orexin, neuropeptide Y, tachykinin, somatostatin/allatostatin-C, CCHamide/gastrin-releasing peptide. Nodes are labelled with taxon-specific shapes and colors, as shown in the key. Connections represent BLAST relationships with a P value > 1e^-40^. All family names of receptors are labelled in the figure. The accession numbers for the receptors shown in this figure are provided in additional file 2.

**Additional file 8. **CLuster Analysis of Sequences (CLANS) of *A. rubens* kisspeptin-type receptors (ArKPR1-11) and other kisspeptin-type receptors from bilaterian taxa. BLOSUM62 cluster map of kisspeptin-type receptors and the closely related galanin/allatostatin-A-type receptors. Nodes are labelled with taxon-specific shapes and colors, as shown in the key. Connections represent BLAST relationships with a P value > 1e^-40^. Galanin/allatostatin-A-type receptors are enclosed within the dashed line. Names shown in dark blue with an associated symbol containing a blue dot are the receptors for which neuropeptide ligands have been identified experimentally in this study or others. Names shown in black with an associated symbol containing a black dot are *A. rubens* kisspeptin-type receptors for which neuropeptide ligands have yet to be identified. The accession numbers for the receptors shown in this figure are provided in additional file 3.

**Additional file 9. **Source sequence data for the analysis of the structure of genes encoding kisspeptin-type, galanin-type and allatostatin A-type receptors shown in Fig. 2A and additional file 10 and genes encoding kisspeptin-type receptors in other echinoderms.

**Additional file 10. **Comparative analysis of the structure of genes encoding galanin-type receptors and allatostatin-A-type receptors. The exon/intron structure of genes encoding galanin/allatostatin A-type receptors from *Homo sapiens*, the starfish *A. rubens*, and three protostome invertebrate species are shown. Exons are shown as rectangles, with non-coding regions white and protein-coding regions black or colored (regions encoding predicted transmembrane domains 1-7 are shown in red, orange, yellow, green, blue, dark purple and light purple, respectively). Introns are shown as lines, with intron length (bases) stated underneath. Intron phase is stated above the line at the start of introns that interrupt coding exons. Species names are as follows: Hs (*Homo sapiens*), Ar (*A. rubens*), Dm (*Drosophila melanogaster*), Ac (*Aplysia californica*) and Cg (*Crassostrea gigas*). A list of ID numbers are shown in additional file 9.

**Additional file 11. **Data analysed to determine the number of genes, if any, located between genes encoding kisspeptin-type receptors in the *A. rubens* genome. The results of this analysis are shown in Fig. 2C.

**Additional file 12. **Accession numbers for the precursors of neuropeptides included in the alignments shown in Fig. 3.

**Additional file 13. **Sequences of chordate kisspeptin-type precursors (A) and precursors of neuropeptides that are candidate ligands for kisspeptin-type receptors in echinoderms (B-D). The N-terminal signal peptide is shown in blue, the neuropeptides predicted or shown to be derived from these proteins are shown in red (with the C-terminal glycine that is a substrate for amidation shown in orange) and monobasic or dibasic cleavage sites are shown in green. The neuropeptides (red) are named in accordance with the precursor they are derived from and their relative position in the precursor, which explains the nomenclature used in Fig. 3. Species names are abbreviated as follows: Hs, *Homo sapiens*, Lo, *Lepisosteus oculatus*, Bf, *Branchiostoma floridae*; Ar, *Asterias rubens*; Sp, *Strongylocentrotus purpuratus*; Aj, *Apostichopus japonicus*.

**Additional file 14. **Comparison of luminescence measurements of CHO-K1 cells transfected with the *A. rubens* kisspeptin-type receptors ArKPR1-11 at 35 seconds after exposure to candidate peptide ligands (10^-5^ M). Each bar represents mean values (± S.E.M.) from at least two independent experiments, with each experiment performed in triplicate. ArKP1.1 (green), ArKP1.2 (pink), ArKP2.2 (purple), ‘cocktail’ of L-type SALMFamide precursor derived peptides ArS1.1-7 (blue), ‘cocktail’ of F-type SALMFamide precursor derived peptides ArS2.1-8 (red) and assay media as a negative control (black). Triton X-100 (olive green), which triggers luminescence via receptor-independent mechanisms, was tested as a positive control to check for cell viability. The source data for these experiments are provided in additional file 15.

**Additional file 15. **Receptor assay source data for the graphs shown in Fig. 5 and in additional files 14, 19 and 20.

**Additional file 16. **Structure of genes encoding precursors of candidate ligands for kisspeptin-type receptors in other echinoderms. Comparison of the exon/intron structure of genes encoding precursors of candidate ligands for kisspeptin-type receptors in echinoderms and genes encoding kisspeptin-type precursors in three vertebrate species. Exons are shown as rectangles, with non-coding regions white and protein-coding regions black or colored (regions of exons encoding the N-terminal signal peptide, neuropeptides and predicted monobasic or dibasic cleavage sites are shown in blue, red, and green, respectively). Introns are shown as lines, with intron length (bases) stated underneath and intron phase stated above. Species names are abbreviated as follows: *Acanthaster planci*, Ap; *Strongylocentrotus purpuratus*, Sp; *Apostichopus japonicus*, Aj. The accession numbers for the sequences of the precursors shown in this figure are listed in additional file 17.

**Additional file 17. **Source sequence data for the comparative analysis of the structure of genes encoding precursors of candidate ligands for kisspeptin-type receptors in the starfish *A. rubens* and genes encoding vertebrate kisspeptin precursors, as shown in Fig. 4, and genes encoding precursors of candidate ligands for kisspeptin-type receptors in other echinoderms, as shown in additional file 16.

**Additional file 18. **Mass spectra for peptides derived from the *A. rubens* precursor proteins ArKPP1, ArKPP2, ArL-SALMFaP and ArF-SALMFaP as detected in radial nerve cord extracts.

**Additional file 19. **Concentration-response graphs for *A. rubens* kisspeptin-type receptors (ArKPR2,4,5,10,11) that were not activated by any of the candidate ligands tested. Key: Green = ArKP1.1, Pink = ArKP1.2, Purple = ArKP2.2; Blue = ‘cocktail’ of neuropeptides derived from the L-type SALMFamide precursor (ArS1.1-7); Red = ‘cocktail’ of neuropeptides derived from the F-type SALMFamide precursor (ArS2.1-8). Each point represents mean values (± S.E.M.) from at least four independent experiments, with each experiment performed in triplicate. Luminescence is expressed as a percentage of the maximal response observed in each experiment. Triton X-100 (olive green), which triggers luminescence via receptor-independent mechanisms, was tested as positive control to check for cell viability and the response to Triton X-100 in each experiment was assigned as 100% luminescence. The source data for these experiments are provided in additional file 15.

**Additional file 20. A**. Concentration-response graph comparing the potency/efficacy of S2 (S2.3; EC_50_ = 2.93 x 10^-8^ M) and a ‘cocktail’ of neuropeptides derived from the F-type SALMFamide precursor (EC_50_ = 1.33 × 10^-9^ M) as ligands for ArKPR6. **B**. Concentration-response graph comparing the potency and efficacy of S1 (S1.4; EC_50_ = 4.79 x 10^-9^ M) and a ‘cocktail’ of neuropeptides derived from the L-type SALMFamide precursor (EC_50_ = 2.43 × 10^-9^ M) as ligands for ArKPR7. Each point represents mean values (± S.E.M.) from at least four independent experiments, with each experiment performed in triplicate. Luminescence is expressed as a percentage of the maximal response observed in each experiment. The source data for these experiments are provided in additional file 15.

## Data Availability

All datasets generated or analyzed during this study are included in this published article and the supplementary additional files. The *A. rubens* genome sequence data analyzed in this paper are publicly available via the NCBI accession number PRJEB33974 [44].

## Abbreviations

AjKissR1: *Apostichopus japoncius* Kisspeptin-type receptor 1
AjKissR3: *Apostichopus japoncius* Kisspeptin-type receptor 3
ArKP1.1: *Asterias rubens* Kisspeptin-type peptide 1.1
ArKP1.2: *Asterias rubens* Kisspeptin-type peptide 1.2
ArKP2.1: *Asterias rubens* Kisspeptin-type peptide 2.1
ArKP2.2: *Asterias rubens* Kisspeptin-type peptide 2.2
ArKPP1: *Asterias rubens* Kisspeptin-type precursor 1
ArKPP2: *Asterias rubens* Kisspeptin-type precursor 2
ArKPR1: *Asterias rubens* Kisspeptin-type receptor 1
ArKPR2: *Asterias rubens* Kisspeptin-type receptor 2
ArKPR3: *Asterias rubens* Kisspeptin-type receptor 3
ArKPR4: *Asterias rubens* Kisspeptin-type receptor 4
ArKPR5: *Asterias rubens* Kisspeptin-type receptor 5
ArKPR6: *Asterias rubens* Kisspeptin-type receptor 6
ArKPR7: *Asterias rubens* Kisspeptin-type receptor 7
ArKPR8: *Asterias rubens* Kisspeptin-type receptor 8
ArKPR9: *Asterias rubens* Kisspeptin-type receptor 9
ArKPR10: *Asterias rubens* Kisspeptin-type receptor 10
ArKPR11: *Asterias rubens* Kisspeptin-type receptor 11
ArF-SALMFaP: *Asterias rubens* F-type SALMFamide precursor
ArL-SALMFaP: *Asterias rubens* L-type SALMFamide precursor
ArS1.1: *Asterias rubens* SALMFamide1.1
ArS1.2: *Asterias rubens* SALMFamide1.2
ArS1.3: *Asterias rubens* SALMFamide1.3
ArS1.4: *Asterias rubens* SALMFamide1.4
ArS1.5: *Asterias rubens* SALMFamide1.5
ArS1.6: *Asterias rubens* SALMFamide1.6
ArS1.7: *Asterias rubens* SALMFamide1.7
ArS2.1: *Asterias rubens* SALMFamide2.1
ArS2.2: *Asterias rubens* SALMFamide2.2
ArS2.3: *Asterias rubens* SALMFamide2.3
ArS2.4: *Asterias rubens* SALMFamide2.4
ArS2.5: *Asterias rubens* SALMFamide2.5
ArS2.6: *Asterias rubens* SALMFamide2.6
ArS2.7: *Asterias rubens* SALMFamide2.7
ArS2.8: *Asterias rubens* SALMFamide2.8
ArTKR1: *Asterias rubens* Tachykinin-type receptor 1
ArTKR2: *Asterias rubens* Tachykinin-type receptor 2
BjGPR54L-1: *Branchiostoma japonicum* GPR54-like receptor 1
BLAST: Basic Local Alignment Search Tool
cDNA: Complementary DNA
CHO: Chinese hamster ovary
CLANS: CLuster ANalysis of Sequences
EC_50_: Half maximal effective concentration
GPR54: G-protein-coupled receptor 54
HPLC: High-performance liquid chromatography
Kiss1: Kisspeptin precursor 1
KissR1: Kisspeptin receptor 1
KP10: Kisspeptin-10
KP13: Kisspeptin-13
KP14: Kisspeptin-14
KP54: Kisspeptin-54
KPP1: Kisspeptin-type precursor 1
KPP2: Kisspeptin-type precursor 2
NCBI: National Center for Biotechnology Information
S1: SALMFamide-1
S2: SALMFamide-2
