# Supplementary material for: Receptor deorphanization in an echinoderm reveals kisspeptin evolution and relationship with SALMFamide neuropeptides
Source: BMC Biol. 2022 Aug 24;20:187. doi: 10.1186/s12915-022-01387-z (PMC9400282; doi:10.1186/s12915-022-01387-z)
Supplement: Supplementary file 1 — Additional file 1. Transcript sequences and protein sequences of Asterias rubens kisspeptin-type receptors ArKPR1-11. [file 12915_2022_1387_MOESM1_ESM.docx]

1 gacaccactacgccaacctgaccgtcaaaccaaaccgtctacgtcataatgacgtcatat

**M T S Y**  4

61 gaggtggacattacgggtgtgttgaatgacacctcatcaaaatcatggcttgatcaggct

**E V D I T G V L N D T S S K S W L D Q A** 24

121 atggagcccggcccagaaacagtgttcgtgccagtagtctggtccctcattatagccgtt

**M E P G P E T V F V P V V W S L I I A V** 44

181 ggagtcaccactaatgctgtagtagtctacgtggtcatgctgcatatgaagatgacaaca

**G V T T N A V V V Y V V M L H M K M T** **T** 64

241 gtgacaaactactacatcgtgaacttagcactgaccgatatatcgtatctgctgttttgt

**V T N Y Y I V N L A L T D I S Y L L F C** 84

301 acaccgttcactacgttgacgttcacattgtatggttacctgactgaggggatgtgtaga

**T P F T T L T F T L Y G Y L T E G M C R** 104

361 gttatatcgtatgtacaacaggcgtctgttcatgcaacctgtatgaccctgaccatcatg

**V I S Y V Q Q A S V H A T C M T L T I M** 124

421 agtgcagatcgctacttcgccatcgtgtacccaatcaagtcaatgaagtacagaaccaga

**S A D R Y F A I V Y P I K S M K Y R T R** 144

481 cgattgtcgtttcttatcaaccttagtgtttggatcctttcctacattatggcaatacca

**R L S F L I N L S V W I L S Y I M A I P** 164

541 gctcccatcttcgtagaagcagagagccacttctactacacgggcgagaaatacttctgc

**A P I F V E A E S H F Y Y T G E K Y F C** 184

601 tttgaagcattcagcacgccaaccaagcaagccgtgtactacacctacgtcctcgtcttc

**F E A F S T P T K Q A V Y Y T Y V L V F** 204

661 acctacatcttacctctaattgtgatatgcgcatgttattccctcctactacgaacgacg

**T Y I L P L I V I C A C Y S L L L R T T** 224

721 tggacgaggcatcatccctcggcacaaaacagtcaccgaacccggcaaaacctcctccaa

**W T R H H P S A Q N S H R T R Q N L L Q** 244

781 aaacgacgcatgacccgcatggttcttgttgttgtttgtctattttttgtgtgctggtta

**K R R M T R M V L V V V C L** **F F V C W L** 264

841 ccaacacatgtatttaactgttgggaacgctacagcacccatgatttcccctactacagc

**P T H** **V F N C W E R Y S T H D F P Y Y S** 284

901 gacgcggtgtactatgccagaattctagcagtgacattagcctattccaattcatgcctt

**D A V Y Y A R I L A V T L A Y S N S C L** 304

961 aatccttttgtgtatgcctttatggggaataacttccgtactagctttaagaaggcgttc

**N P F V Y A F M G N N F R T S F K K A F** 324

1021 ccgttttgcttcaggaggcggattgttatgagaaacacctttcaattagtgaaccgatta

**P F C F R R R I V M R N T F Q L V N R L** 344

1081 cgcagggctagggtgactgaagatggcgggacaagggtgaccgtcctggcagtctgagaa

**R R A R V T E D G G T R V T V L A V *** 362

1141 aacaatactgtcaataaccctagcaagaatataaatacctttaaaaacaaaccaacaata

1201 gggattgaacagcgcaattaatcctataatgctctatgacatctgtatcacataaaaaca

1261 cccactagacatttttggttttatcgacgtggcgttgatttgtccttactttatgatatt

1321 tataaataaacatcaaatcttgaatctgttacttaaaatggcatcctttattttgtttta

1381 tctttgtttcctatacattattgcatagactttttcgcaagtacaaataattttttgcgc

1441 aagcgctaactgtttaatggagatgcttgctggtctatctagcggtcaaacttgatcgct

1501 atagctagactagcaagcaccaaattccaccctaaagcgagtttttgcgataaggtctat

1561 tggcatctgaaagtgcgccctcttttgtcagttaaacaatattttttctttaactcttct

1621 ttttctttaacttgatttgaccatcttttaatagtaatctcccaacgtaaccaaatagag

1681 gctaaaagcataatggtctgatccccttatgacatgacaaattagtaccactctaaactg

1741 tgcaaacaaggatacaagacaatagcaaaataataaacaaacattacctgtaaatttcct

1801 tggagcagggggcatccatcttgaattcaatggctaccattaacacattatgacgttcag

1861 cagttagcagactttacaaggtaaatccacgctcacaaggcctcagtgtatccaaacatt

1921 gtgacgttcagcgtcaagtttccagcgacaagtttccgtatggcgccac

**A. *Asterias rubens* kisspeptin receptor 1 (ArKPR1).** The transcript sequence (lowercase, numbering on the left) and the deduced amino acid sequence (uppercase; numbering on the right) are shown. This transcript was identified by analysis of *A. rubens* radial nerve cord transcriptome sequence data (contigs 1100140 and 1083499; scaffold 34497). However, because of absence of overlap in the contig sequenes, PCR cloning was performed to confirm the sequence of this region, using the primers highlighted in yellow. When compared with the sequence obtained from the transcriptome data, the PCR product had three nucleotide substitutions (highlighted in black or pink), none of which affected the encoded protein sequence. The GenBank accession numbers for these transcript and protein sequences are MT358419.1 and QVN25218.1, respectively. The sequences have also been confirmed by genome sequencing (XM_033783456.1, XP_033639347.1) but with polymorphisms, the positions of which are highlighted here in red or pink.

1 gt

3 gaaattgcacacgcactgccgataagaataaatctaccctgatctatagactttgatatg

63 taggactatcattgcgggcatagagtaaaacttccttgtagtttgggcgggaaaatgaca

123 aatgaaaacccgtctgccaataaatgaagcggcctatggcagtggccacataaggtagag

183 gaaagtacttgagcagtaccaaacggctgaacagaacagtggaggtggattctgcaaaat

243 aagagatctggttgaaaaagaggtttggatcccgtggcttttgagtggattactttacaa

303 agctcggcaatgtcaatgcgaacttttactattgttaggtgtatttaggtttctcaagtt

363 tcagtcaagatgacgacctcgatcactcaaactattggatttctcgtgaattcctctgag

**M T T S I T Q T I G F L V N S S E** 17

423 tgctgtcatgagtcaatcgctttcacaaccaaaactctcttcggagaaacgactactact

**C C H E S I A F T T K T L F G E T T T T** 37

483 agcagcatcccaaccatcaccacagacgaagccgcagtggtgcaagccacagcggggcaa

**S S I P T I T T D E A A V V Q A T A G Q** 57

543 gctgcctggcttatcccggtcatctttgggctgatcacggtctgcggcgtggttggtaac

**A A W L I P V I F G L I T V C G V V G N** 77

603 ttcctcgtgatctacgtgattgtccgtcacggtcacatgaagaccgtgacgaactactac

**F L V I Y V I V R H G H M K T V T N Y Y** 97

663 atcgtgaacctagccgttactgatatctcctttctgctctgctgtgcaccgttcacggct

**I V N L A V T D I S F L L C C A P F T A** 117

723 actctatttgtgtctccaaactggctcttcgggagattcatgtgcaagttcgtcttctac

**T L F V S P N W L F G R F M C K F V F Y** 137

783 atgatgcaggtgactggtcagtgcacgtgtctaacactgacagctatgagtgtggaccgg

**M M Q V T G Q C T C L T L T A M S V D R** 157

843 taccaggctattgttcatccaatcaaatcactcaagtctcgtacgaccagagtagcgtgc

**Y Q A I V H P I K S L K S R T T R V A C** 177

903 attgtcaacatgtgcatctggattggatcactgctgatctccgtgccggtggccatcttc

**I V N M C I W I G S L L I S V P V A I F** 197

963 tttgatttatccctctacatcaacctgttggtctgctctgagatgtggcccctggacatc

**F D L S L Y I N L L V C S E M W P L D I** 217

1023 atgttccctggatacgccgtcttcagctttatcctcctctacgtcattcccatcttcacc

**M F P G Y A V F S F I L L Y V I P I F T** 237

1083 atttccgtctgttacagcctcatgctccgcaagctgtggtctcgcgtgtcacctggtgaa

**I S V C Y S L M L R K L W S R V S P G E** 257

1143 gacaacaactcccatcttaacaacgcccgtcagaaacgcaaagtcacccgcatggtcctg

**D N N S H L N N A R Q K R K V T R M V L** 277

1203 gtggtggtgctggtcttcgctatctgctggctgcccacctacatcatcaacctctggatc

**V V V L V F A I C W L P T Y I I N L W I** 297

1263 cgtcttgacccgtacttccccaagaccaacgccacgtatattttcaagatggcggcgcac

**R L D P Y F P K T N A T Y I F K M A A H** 317

1323 acgttgtcgtacgccaactccagcgtcaacccgttcgtgtacgcctttatgggagagaac

**T L S Y A N S S V N P F V Y A F M G E N** 337

1383 tacaggcggtacttcaagaaggcgttcccggtgtgcttccgccagcgagtgagacgtccc

**Y R R Y F K K A F P V C F R Q R V R R P** 357

1443 acggacttcacgacatctcgcacggaccctactggaaacttcaacgggggaactgcgggt

**T D F T T S R T D P T G N F N G G T A G** 377

1503 attggtgggcggactgttgaaaccgttgcgttggactaacaaagtgaatgatttaggatt

**I G G R T V E T V A L D *** 389

1563 aaagtgacatactgcgattctctgtttgtcgaacctacattttattttattgttgtatgg

1623 taactgcatacaaaatgtgtatacatgtataagttcttaataatagtaaggacacctcct

1683 cgacgataatatgtggtcacttattttgtgcacttccttttccttaaaggggaaagtata

1743 cgtttggttatc

**B. *Asterias rubens* kisspeptin receptor 2 (ArKPR2).** The transcript sequence (lowercase, numbering on the left) and the deduced amino acid sequence (uppercase; numbering on the right) are shown. This transcript was identified by analysis of *A. rubens* radial nerve cord transcriptome sequence data (contig 1114028). The GenBank accession numbers for these transcript and protein sequences are MT358420.1 and QVN25219.1, respectively. The sequences have also been confirmed by genome sequencing (XM_033783882.1, XP_033639773.1) but with a single polymorphism, the position of which is highlighted here in red.

1 tt

3 gttcctcgacgcaaatattcaaaaatgtcgactccagccggccttctcaacgtcaccact

**M S T P A G L L N V T T** 12

63 gctatcccgaatgttacagaagcttcaatggatgacgggggagccagtgtgtataccaaa

**A I P N V T E A S M D D G G A S V Y T K** 32

123 ctggttcccgccctaatcggcattataaccctggttggattggttggtaactcgatcgtc

**L V P A L I G I I T L V G L V G N S I V** 52

183 gtctacgtcatcgtgtgccagggacatctgaagacggttaccaactattacattgtgaat

**V Y V I V C Q G H L K T V T N Y Y I V N** 72

243 cttgccataactgacatattcttcctggtattctgcgcaccttttactgcgtcgatttat

**L A I T D I F F L V F C A P F T A S I Y** 92

303 gcaacaccgagttggctgtttggtcgcttcatgtgcaagtttgtcttctacatgatgcag

**A T P S W L F G R F M C K F V F Y M M Q** 112

363 gcgactgcccaagcaacatgtgccaccctgacagcaatgagtatcgaccgttactatgcc

**A T A Q A T C A T L T A M S I D R Y Y A** 132

423 attacagacccgctcaaggctctcaagactcgtacaccgagagtggctatcgtcgtcagc

**I T D P L K A L K T R T P R V A I V V S** 152

483 gtcggtatctggacattctctgcagtgcttgccataccagttgcagtattcttcgacatc

**V G I W T F S A V L A I P V A V F F D I** 172

543 gatgtcgtgcagtttcaaaaccagacatatgacatctgcgatgaaatgtggcctctcaaa

**D V V Q F Q N Q T Y D I C D E M W P L K** 192

603 atcgttaaccagggctacggcgtttactgcttcgtgatgctgtacctcataccactaaca

**I V N Q G Y G V Y C F V M L Y L I P L T** 212

663 atcatcgtcgtttgttactccatcgtgctgaaccgtctctggaaggctgtctcacccacc

**I I V V C Y S I V L N R L W K A V S P T** 232

723 gaggagacccacgctcccgtccaccttcgcatgctgatacagaagagacgaatcacccgt

**E E T H A P V H L R M L I Q K R R I T R** 252

783 atgatcttagctgttatagtcgcatttgccgcttgttggattggaacccacatcatgtcg

**M I L A V I V A F A A C W I G T H I M S** 272

843 ctctggcgtcgccttgatgtgaacttcccaagaacatccacgtccgcccttgtgtttcaa

**L W R R L D V N F P R T S T S A L V F Q** 292

903 accatcgctcatttgttaatgtacttcaactcttgcgtcaacccgtttgtgtacgccttt

**T I A H L L M Y F N S C V N P F V Y A F** 312

963 atgggtgggaacttccggaaacagatggccagagcgtttcctttcttagctgggaaaaag

**M G G N F R K Q M A R A F P F L A G K K** 332

1023 tctgctatgacggaaggcccaactggtagtttaatcaaatcgaagtccacgcaggtttaa

**S A M T E G P T G S L I K S K S T Q V *** 351

1083 actggttaaaactattgattgtatgcttaaaaggtttagttttacaacgttgaacatgga

1143 taacacgtgaagaacgaaacaacgagagatgtctgtcgttgaactacaagggtgattcgg

1203 acaagtatgtgacaaaacaatgcgtttgtttctcgggaagatgtctaaaattatgagttg

1263 tagggcctaccattatctttattcccaaattgaagtcgagtgcctggtatttacccgaca

1323 gaattgtgtgttctttatgtagctcattggcaaagtcaaacgtgaagcaaaacggagtat

1383 atcattgtacatttcctgactctaaattccagacagtggtcgcaataaagtgcgccatca

1443 agcttttgacccgggttgtcactaacttgttcacagagctatggtactgatctctatttt

1503 attgaccgtatactagagatcattgagcattgaccgtgcttaccaccaatttaagcacta

1563 aaggtataccattcttctcttactgcagtgctgaacgaggtttatccggacataaaagaa

1623 acataacttgttgtccatatatcgactgcaacgtatgtacatgcggcaaacaccacattt

1683 gtgttgtcaatcatgtaatacatagaccgggcccagtttcatggctctg

**C. *Asterias rubens* kisspeptin receptor 3 (ArKPR3).** The transcript sequence (lowercase, numbering on the left) and the deduced amino acid sequence (uppercase; numbering on the right) are shown. This transcript was identified by analysis of *A. rubens* radial nerve cord transcriptome sequence data (contig 1113702). The GenBank accession numbers for these transcript and protein sequences are MT358421.1 and QVN25220.1, respectively. The sequences have also been confirmed by genome sequencing (XM_033783844.1, XP_033639735.1) but with polymorphisms, the positions of which are highlighted here in red.

1 gg

3 tacttttccgcgtgacgtattgcctaatttgcatgtcttgcccccgtgtattggtatgcg

63 tatcaaaagcatccgcagctgtgtcaaccagatcacaagcttttcggaacatctgtacat

123 gcacattgattgtaaagggaacgggagtacgaccgtgtctgagactcatcgactaccggg

183 gccagagctgcgttcaacggggcaagtcttcacacataactgcctctatcgtcgtaaaag

243 atcaactccctcatcaggagaagtcctcgcactttcagggaagagacttaagggagactc

303 ccacctgtccctaaagagatagtcaagacaacatccctcgacggatcagagcgtcgaagg

363 cctggtactctctgcggggtcacaagagcacatcgccccgatcgagttccagtaattagg

423 ggacgaggttgtttttgccaggggaagatgacgaccacggtagccacgattttccctttg

**M T T T V A T I F P L** 11

483 agtacactaggcaccaataatgattcagtcgggaacgaaaccttttaccagcaaccggct

**S T L G T N N D S V G N E T F Y Q Q P A** 31

543 gatcttaaaccaccacatcatgcgttgcttatgccaatactttttggcattctgatggta

**D L K P P H H A L L M P I L F G I L M V** 51

603 gtcggcatcgttggtaacaccttagtgatcttcgtcatcttaaagatgcgacaattcaag

**V G I V G N T L V I F V I L K M R Q F K** 71

663 acggtcactaactactacgtcgtcaacctcgccgttgccgacattctgtttctctgtatc

**T V T N Y Y V V N L A V A D I L F L C I** 91

723 tgtgcgccctcaacggctgctcaatacggatcgccttcgtttctcggtggtcgattcatg

**C A P S T A A Q Y G S P S F L G G R F M** 111

783 tgtaaaatggtctactacatgcagtcggtctcagctcaggtgacctgtctattcctagta

**C K M V Y Y M Q S V S A Q V T C L F L V** 131

843 gcgatgagtattgaccgattccaagccatagtcagaccactcaagtcattaaagacccgc

**A M S I D R F Q A I V R P L K S L K T R** 151

903 accctccacaatgcagccacaattagcatcgtcatatggctttttgcagtcacagtctac

**T L H N A A T I S I V I W L F A V T V Y** 171

963 atacccttgttggtcttctttgacacagtggatattccatacaacggcggaataatactg

**I P L L V F F D T V D I P Y N G G I I L** 191

1023 ctctgtaaagagttctggtctaagacatggagtgagctgttctccatttggatcttcctc

**L C K E F W S K T W S E L F S I W I F L** 211

1083 tttacctacacactcccccttgttgtcatctccatctgctatagtatgatgatactgaac

**F T Y T L P L V V I S I C Y S M M I L N** 231

1143 ttatggcagcgcgtcgtgccgacagacgccctcagcggccccgcaaacgacaggaacctc

**L W Q R V V P T D A L S G P A N D R N L** 251

1203 agacagaaacgcaagataacatggatggttctgacagtcgtgattgtgtttgccgtgtgc

**R Q K R K I T W M V L T V V I V F A V C** 271

1263 tggcttcctgtacatgttatacagatctggatggaatttgatgtgaattttccgtacacc

**W L P V H V I Q I W M E F D V N F P Y T** 291

1323 atggcaacgctggatttcaaggcggctgggcatgcccttatctacattaactcctgcgct

**M A T L D F K A A G H A L I Y I N S C A** 311

1383 aaccccttcatatacaccttccttggggagaacttccagaagaactttaagaaaactttc

**N P F I Y T F L G E N F Q K N F K K T F** 331

1443 cactgctgctttaagaaggtacggccgacacgagagggcgctggtcatgtagccaacacc

**H C C F K K V R P T R E G A G H V A N T** 351

1503 ggcacgcagcagggatcgtccagtgaacaggcagggacgagacgaaagacaggaggcgga

**G T Q Q G S S S E Q A G T R R K T G G G** 371

1563 cgtactcaaatgagcacattgacgtcatcgaatgttgaagctaaaggaatagagcagatc

**R T Q M S T L T S S N V E A K G I E Q I** 391

1623 gataataaccactgctgagagaccaaatcttatcaatacacccaaacaactgtctcacca

**D N N H C *** 396

1683 ttttgtattaccattacacatgggagacaaggtcccacccaaccacacgaggtccgactt

1743 aagggtaccatggaggtagaaatggaggtctatttctacctccagggtgggacgacgttt

1803 ttgaataatgagacctactcctttaacacagcaccatgtgatggtctacaaggtgttacg

1863 gcgcaatatgctgccaatcaagtacactgggttaacttacatgcgttacacaacacacg

**D. *Asterias rubens* kisspeptin receptor 4 (ArKPR4).** The transcript sequence (lowercase, numbering on the left) and the deduced amino acid sequence (uppercase; numbering on the right) are shown. This transcript was identified by analysis of *A. rubens* radial nerve cord transcriptome sequence data (contig 1116161). The GenBank accession numbers for these transcript and protein sequences are MT358422.1 and QVN25221.1, respectively. The sequences have also been confirmed by genome sequencing (XM_033783522.1, XP_033639413.1) but with a single polymorphism, the position of which is highlighted here in red.

1 taaaagaaatgtaggattattcgctcaattaaacaaacgaagtcggtgtattgaaaacat

61 ttcgaagtgaccaatatgtaactagtggttattctacgagcgagcacacaacgtgttggt

121 caattgccaaaaagagtgtcttcgtgacgtctgactgctcgtgcgcatgacgtgtcgcag

181 aaagaatcaccgcacatatttttcttcttctaccgtgtgcggtgcgtgcggcatttttaa

241 tgccaaccaacttgaaagagaagtatttgtattctgagtcttcaccatagctgaaccaac

301 caacatgtgatttgcagtttttgaagcaagagtcagagaatatcaacttctttaatctgc

361 gctgggttggaaattggaacgaaggcttttcccgaacagattgcgccttaatgacaaatc

421 ctcgttgttgaatatcggtattggactctgccacgaaatacttgccttggtggaagacgt

481 tcgtacaaacctacatcaatcaaacacgaggactagtgtagcttggaacaggacagtaac

541 tgaacagatatctattttcctggttggaagggtttacaacagggaggtgaagttggcggt

601 gaatgtgatgcgcccggagcctacttcaagggcgagatggccattaggtgtctgtagctg

661 ttgatcaacctttgtcttcatttcgagaacgagagaaaacgtgttctcattgcttttcaa

721 cggtatgaaaacaccagtagaataatgtgaagctcacatgaaaggacactgtactgtgtc

781 atcatgatgtcaactgcagatcaccaactcggcctattacaccagatccagatcagtaac

**M M S T A D H Q L G L L H Q I Q I S N** 19

841 ttatcaacgagcatcatggaagactttgaagtcgaaaattattacgactcaaacaatacg

**L S T S I M E D F E V E N Y Y D S N N T** 39

901 agcgactatgtgttcgacatctcagtcagtatattcaacgccaccagcagaattctggta

**S D Y V F D I S V S I F N A T S R I L V** 59

961 cctacgacattcttcctgcttgccttgatgggacttgtcggtaactgttcggtgatgttt

**P T T F F L L A L M G L V G N C S V M F** 79

1021 attatctgtcgacacaccgatatgcagacggttacgaactatttcatcgctaatctggca

**I I C R H T D M Q T V T N Y F I A N L A** 99

1081 atcacagatgtagcaacagtgttgttctgcatactgcctacagccttacagaattcaggt

**I T D V A T V L F C I L P T A L Q N S G** 119

1141 attatacctatgagcactggtgtatgcaaaggagtcaactacattcagtttgtcactgtg

**I I P M S T G V C K G V N Y I Q F V T V** 139

1201 caggctacgtgttgtacccttactgcaatgtcgattgacaggtactttctgattgttcac

**Q A T C C T L T A M S I D R Y F L I V H** 159

1261 gctgtacgttctagaaggagccgtactacaaacaaggtcctgattataaacgtcacgatc

**A V R S R R S R T T N K V L I I N V T I** 179

1321 tgggcagtttcattcatgatgcactctccagtggcagtcgtatccaaagtaaccagctac

**W A V S F M M H S P V A V V S K V T S Y** 199

1381 aactactgcgaaacagtttttgggactgttcgtggggagagagtgtttcaaactttcgca

**N Y C E T V F G T V R G E R V F Q T F A** 219

1441 acgctctcgatgtacgtggtgccactgattataaacctggtgtgttacatcagtatactg

**T L S M Y V V P L I I N L V C Y I S I L** 239

1501 ctacaagtgtggacgagaacagcacgagggacggagagtgcgcaggcccaggaaagagcc

**L Q V W T R T A R G T E S A Q A Q E R A** 259

1561 gttcgacgcaaacggaaaatcacgcgcatggtgtttgtcgtggtgttactgtttgctgtt

**V R R K R K I T R M V F V V V L L F A V** 279

1621 tgttgggctcctaagcacttctttagaatgtggattgcttttgactatgtggagtttcat

**C W A P K H F F R M W I A F D Y V E F H** 299

1681 atgacgagtaatagacactatgtactaatgggctcgttgcagtttgtcgccctctgtctt

**M T S N R H Y V L M G S L Q F V A L C L** 319

1741 gcttatggcaatagctgtgtcaatccatttgtctacgcattcaccacaactagtttcaag

**A Y G N S C V N P F V Y A F T T T S F K** 339

1801 aagtacttcaagaaggttttcaaaccgtgctgtagatttgaagataggcaggccagaacg

**K Y F K K V F K P C C R F E D R Q A R T** 359

1861 agtgtgaatcagtcgagggtttcaaaagttataactggcgaagagtcactggtttgacca

**S V N Q S R V S K V I T G E E S L V *** 377

1921 ttatagatggcggttcgcatgacagaaaagtcataaaactaccaactggattacagtttg

1981 aaatacaagaaattaaagcctgcactttcttgcacatttccacaagtaaatgactttcac

2041 atgcaatcgtctgtgggcacggcagtatgggtcttttggacggttattgtggtgttttat

2101 aggaagacatgaaaatagacattacagtaggattaattcaacctagataacaatcacgtt

2161 atacttagataatacgataattttacagaaaaacattacatttttaccgaccaaaccatc

2221 tgactccaaccctctcccttatttctcgtttcactcaaaaccgttggatcaccttcaata

2281 cactattcccaaaacaagttgaatgacttaactgttttgccagtgttgagaaactacccc

2341 ttttaaacccggaaggtggatgcgttgtatacacaaatcaaattctgacgtggtttccac

2401 aacttaggtttatattttatattttccttgttttgttacaacatacgtctctgcagacta

2461 ctctccaaacatatgattgctctgaaagtgcgtatatgatacgcattcagtcgttcttga

2521 ctgtaaatcctttggtaattgcaatgcacagatcaaataatacggtttcattaaaaccac

2581 cccgatttagatggg

**E. *Asterias rubens* kisspeptin receptor 5 (ArKPR5).** The transcript sequence (lowercase, numbering on the left) and the deduced amino acid sequence (uppercase; numbering on the right) are shown. This transcript was identified by analysis of *A. rubens* radial nerve cord transcriptome sequence data (contig 1121461). The GenBank accession numbers for these transcript and protein sequences are MT358423.1 and QVN25222.1, respectively. The sequences have also been confirmed by genome sequencing (XM_033778438.1, XP_033634329.1).

1 tg

3 ctgtgaggaagagttctccagtcttgaagaaacgtaactaaagaacaccacctttgctag

63 agtgaggggtcactgttttacgaaccatcccatggcaccaatgttgctgtgtggggcgac

123 ggttcgctcccgttacaattactgactttttgtgcttcagatcaaataaaaatacctgct

183 ttgtttatcacctctttcagcttcgataaagaaccaaccatgacggactcgcaagcattt

**M T D S Q A F** 7

243 gttacggatatcgctacgacaatggtggaggtgtatggaccaaccaacttgactagcttg

**V T D I A T T M V E V Y G P T N L T S L** 27

303 tttgacagcacaagctcttacatcagtgggccgtgtaccgagggtaactccagcctcggg

**F D S T S S Y I S G P C T E G N S S L G** 47

363 gatggggtaactccgtctggtatgacccctgggttgcagttccctgattcctttttaaac

**D G V T P S G M T P G L Q F P D S F L N** 67

423 ccaatccggattgtcatgcccatcattatggccatcatttccatcatcgggctagtgggt

**P I R I V M P I I M A I I S I I G L V G** 87

483 aacggcactgtcctctgtatcatcttcaaatatcgagagatgcagaacattaccaactat

**N G T V L C I I F K Y R E M Q N I T N Y** 107

543 tttatcgctaatctggcactgacggatgttgccatgctgggcatctgcgctattcccact

**F I A N L A L T D V A M L G I C A I P T** 127

603 gcggctggtctagcaggaatgaaaatggaggaaggactgtgcaagggtgttaactacatg

**A A G L A G M K M E E G L C K G V N Y M** 147

663 cagtttgtagcggtccaggcaacatgctgtaccctgacagcgatgtcaatagacaggtat

**Q F V A V Q A T C C T L T A M S I D R Y** 167

723 ctccttatcgttcacgctgtcaaatcaagaaaaactagaacgacgactagggccatcata

**L L I V H A V K S R K T R T T T R A I I** 187

783 gtcaacgtggcagtctggattgcgtctttcatcgtgcacagtccagtagcgatattctac

**V N V A V W I A S F I V H S P V A I F Y** 207

843 cagctaaccgatcaggggtgcgaacttaatatcggtaaggacggcaatggagctaagtac

**Q L T D Q G C E L N I G K D G N G A K Y** 227

903 tactacctaggtgctttcttatccatgtacgtcattccactgatgatcatactcatgtgc

**Y Y L G A F L S M Y V I P L M I I L M C** 247

963 tatgccaagatcctcatcatcgtctggaggaagacatccgctggtacggagagcgcccaa

**Y A K I L I I V W R K T S A G T E S A Q** 267

1023 gcccatgagcggtccattcgtcagaagcgcaagatcacccgtatggtttttatcgtagtc

**A H E R S I R Q K R K I T R M V F I V V** 287

1083 ctcctctttgctgtctgctgggcaccaattcactgtattcttctgtgggagcagtttaaa

**L L F A V C W A P I H C I L L W E Q F K** 307

1143 accgaggatataacgaatcttagcacggcgatggcatttgcatgtcttcgcttgtttgcc

**T E D I T N L S T A M A F A C L R L F A** 327

1203 ttgtgtctggcttacgccaacagtctcacgaacccatttatttatgccttcacaacagca

**L C L A Y A N S L T N P F I Y A F T T A** 347

1263 agctttaagaaacatttcaagaaggttctttcctgctcaacacccactgagagcaaggaa

**S F K K H F K K V L S C S T P T E S K E** 367

1323 cagaaaactatgggaccaacgagcacctacaagaaaattggaaagtacgtcaatgaatac

**Q K T M G P T S T Y K K I G K Y V N E Y** 387

1383 tcttcagtcaacacatgtgatacaaaagtttgaaaagggttcatagggtctacaatgttg

**S S V N T C D T K V *** 397

1443 tgttttgatacctctccccgacgagcccatattagtgttgttgttgttgctaggaaatat

1503 atgcacattcttaaattgtcaaaatacttgtcattgtctgatctgtcaagcaactggcgc

1563 ccatcagtcccatatgtgtaccgggcgttaaccctgagtacgcatccgagagtttcactt

1623 tccttcctgtctttttaaagatacactgtccatttgaggtgctctacgacgccataaatt

1683 gattgggccaatttggctgaatctatacagcacttacattagcatgaactgcacatgtcc

1743 gagcgttatgatagagagttaatgggttactttgttggactgtttacagattcctggcta

1803 cggccccgtccagttttagatcgagacaaactccgcatgacctgtaaactccgggaatat

1863 accgtctgcgccacaacgtgtacggtcagtttttttgaatttgccacacctactaaccgg

1923 ttgaaagggcgccgtttcgaacatgtgcaccattgatgtagcagtgtcagctgcattgac

1983 tacctcaactgaacactgtgacgtagaagtgtatatcagtgtacataaccgatgctttcg

2043 gttgattgttaatatatctgactttaatgtccgattccgtttgttttctctcccagtgaa

2103 gtgagcatccacttacacttgaagtgtaaacatcgcaatggctgaccttattgtccgggt

2163 tttcgggaacaattcctttttggcaccgtactcactttctaactggggacctttacccaa

2223 ccgaggactctattgtcacctgttgtttttagtccccggttggaatgtttctacacctaa

2283 ccaaacttgtgtttgtctgcacttgtactatactatggagtgattctccgatttattttc

2343 ttgttcagctaatccattttgttttgcactttgtatattcaaacgtaaagtgtctcttac

2403 acaaccagttttgtcaaaaattaactcttcgtagaattatagtaacttcacattatcaaa

2463 cggtattatatgcataatgacgtacgcttcgatattgtatagtttatgctccctttataa

2523 cggcaaacaattgttttgtttccggttgtaagcatttggattttcgaaaccgttggaaac

2583 cttgtattttattagaccttttaagtgatgcggccaccttattgggtatgttgctgtaca

2643 tataaaatgctttcgagagctttttgaagccgaaatcttacaatgtattgcttacagatg

2703 aaacggaaatttgacattttaatcgacaaatcagcttaacgttttaaacaaacatgtaaa

2763 gtttaactttatttgttgtgtttttccccattgtcgtaaccacattgtgtctacgctact

2823 gccgcaaatatttataatcgtcagtgttgacaatttgtatagaacttgtataaacaaaag

2883 tctatgcgtgtctataatagtcgctgatgcaacgtggacaatattgttttcttaaattaa

2943 aatacaaatacaaacattaatgagtatagtcaacggtgacaaacgacgtattatatcaag

3003 taatacaattcaagttacgtcagcgttcagactgggccccatacatgtaaatacaccgac

3063 atttctctatactttgattgataaattggggtttgcaggataccgttgcgtaacattgcg

3123 tatccgatatgtgtagtcatggtatcttgttatattattcctttttctaaaga

**F. *Asterias rubens* kisspeptin receptor 6 (ArKPR6).** The transcript sequence (lowercase, numbering on the left) and the deduced amino acid sequence (uppercase; numbering on the right) are shown. This transcript was identified by analysis of *A. rubens* radial nerve cord transcriptome sequence data (contig 1123777). The GenBank accession numbers for these transcript and protein sequences are MT358424.1 and QVN25223.1, respectively. The sequences have also been confirmed by genome sequencing (XM_033778496.1, XP_033634387.1), with the exception of the C-terminal region of the protein highlighted in red where there is evidence for the existence of an isoform encoded by alternatively spliced mRNA.

1 gtggctgcatgcagttcgcagaccgtacaaaacgacacgtgctggttgagacccgttcac

61 tctctcccatcagtctattgcgatagcattattgtctttttaaaacaacagttgagaagt

121 tgaggcggtgatgttgcctgttcctgcggtgggcctgtatctgcgaactgataatttgta

181 actactatcactgcttattcatttagctctgctacaacattgaacagttctacatcattg

241 tatttcccgagagcaattttgttttcgcgccaccctcaaaatcaaacgctgattgagtct

301 gggaaccttgtggatgctgctgtacgaaactatctctgaacaatggagggaacctgtggc

**M E G T C G** 6

361 aactgctccagtgatgtcgggggtgatgtcgatccgattactggtataatattccctgtc

**N C S S D V G G D V D P I T G I I F P V** 26

421 cttatggtgttaattgccgcagtggggatcactggtaactcattggtacttcatatcatc

**L M V L I A A V G I T G N S L V L H I I** 46

481 ttcagacatcgtgatatgcgaactgtgaccaacagttttgtagccagtttagcgttgagt

**F R H R D M R T V T N S F V A S L A L S** 66

541 gacattgctatgctgacgatgtgcgtcataccaacggctaccgtggccattactgaaaca

**D I A M L T M C V I P T A T V A I T E T** 86

601 tggaaacttggggattttctttgcaaggcagcgagttacacgtcatttgtgactgttcaa

**W K L G D F L C K A A S Y T S F V T V Q** 106

661 gccacttgtttgacgctaacagccatgacggttgaccgttactatttgatagtacacgca

**A T C L T L T A M T V D R Y Y L I V H A** 126

721 gtcaagtctcgaaacacacgcactgtgatgaaagctgtagtcatcaatgtggcaatctgg

**V K S R N T R T V M K A V V I N V A I W** 146

781 atcttctcttctttgattcatctcgcgacccctgtcttcacagcggttgactcagacagc

**I F S S L I H L A T P V F T A V D S D S** 166

841 aattgtgccaatacgttcccgaatccagacgtagatgctaaaatctacggagtgtatgcc

**N C A N T F P N P D V D A K I Y G V Y A** 186

901 ttcttgggaatgtacatcatcccactgatgttgattatgttctgctacgcgaaaattctc

**F L G M Y I I P L M L I M F C Y A K I L** 206

962 attcaaatctggcaaaaaacttcgggtggaacggagagcgcacaagcccacacccgtgcc

**I Q I W Q K T S G G T E S A Q A H T R A** 226

1022 ctgaaaaggaaaaggaaaatcacacgaatggtacttattgtggtagttctattcgcgttc

**L K R K R K I T R M V L I V V V L F A F** 246

1082 tgctgggcgccacttcaaatattcatcatctggacgaattttaattatgcccaagtgtca

**C W A P L Q I F I I W T N F N Y A Q V S** 266

1142 gagatgaggaaagacgttttcctttttctgcgaggttcattccaatgtatggcgtacgcc

**E M R K D V F L F L R G S F Q C M A Y A** 286

1202 aatagttgcgtgaacccatttgtgtacgcgttcacaacgacaagcttcagaaaatacttc

**N S C V N P F V Y A F T T T S F R K Y F** 306

1262 agaaaaatgttcgcaacgtgccgtggaaacgtctacaaggatcgcactagtatcagcatc

**R K M F A T C R G N V Y K D R T S I S I** 326

1322 agtatgacaactaagacagagagattagcagctgaagaagattcttcaatttaaaacagt

**S M T T K T E R L A A E E D S S I *** 343

1382 gtggatgaaacaacaagacattcttaaagcaggaacacatcatacagaatgagaacacac

1442 aacgaaatagagaggtttacgggaccgtttgtagtggatatagtagagcatcgtgtgtat

1502 tcagagcagtgtttgtattttgtgtttgataccggttaaggtaagaaaacattggaaatt

1562 ggaaaccgatacatgcagaaggaacaacggaacacgaaaacgctctcagtttacttgaac

1622 taggtcatttctgaaacgggaaacgcggatgtcgtgggcatcatcgaagagccgagagcc

1682 aaacggaccaaacggaggagaaacaacaggcacgctttgattgcccttcctccttttcaa

1742 tcagccagccagccgagcttccttcttcgggcaacccctttcccctctgtgtgtgtcgtc

1802 tccctgttagtatgctaatttttaactagcaaagggtcaagtaacgcattggaaaatcca

1862 aagggcaacactgggtaatgagttaatccactgagtaagtgtagacaatgatttacaaag

1922 catgctttaaagggggcgaacgcaacaaacaaattatatgtgtgtataaactacataaaa

1982 tacgggggtggctagatggaaatgtgatagagaggttgccaggtataatggtatattatt

2042 tttaaagacggagacataaactcggtaatgttttcaatgaaagaggaagacgaaattaaa

2102 ttagcattacataattggttttgctagca

**G. *Asterias rubens* kisspeptin receptor 7 (ArKPR7).** The transcript sequence (lowercase, numbering on the left) and the deduced amino acid sequence (uppercase; numbering on the right) are shown. This transcript was identified by analysis of *A. rubens* radial nerve cord transcriptome sequence data (contig 1118327). The GenBank accession numbers for these transcript and protein sequences are MT358425.1 and QVN25224.1, respectively. The sequences have also been confirmed by genome sequencing (XM_033778997.1, XP_033634888.1) but with a single polymorphism, the position of which is highlighted here in red. Furthermore, the predicted start codon for XP_033634888.1 corresponds to the methionine residue at position 115 in this sequence.

1 tagtaaagcagacccacttgtcatctttagcggaacctgttcgagcttattttgccaaat

61 gcactgtgggcactgatcctttctcattggatttgtggatacctctgcactgaaatgaaa

121 cagcggctactggcttcctcctcatcgttttagcaagagccagcaaagttccgcatctcg

181 aggatttagaatattcagttgactcgtcgcaacttcgaagtggggattgttctactaaca

241 attgcacactttcacctgtgacctaaactcaactcaagaaagaaagaactagcgagaaaa

301 aaaaaagacatgtcgagtggggattttctaagtgatagttattcgatgtactataatgat

**M S S G D F L S D S Y S M Y Y N D** 17

361 aacgctaccgtcgaggcggcatcactgggccccagtgccgtcttagtgcccctgactctc

**N A T V E A A S L G P S A V L V P L T L** 37

421 ggcatcatcgcggtcctggccgtggtgggcaacgcactagtcatctatatcgtgctccgg

**G I I A V L A V V G N A L V I Y I V L R** 57

481 tatcggaacatgaggagcagtgtgaccaacttttatatcatgaacgtagccatcagtgat

**Y R N M R S S V T N F Y I M N V A I S D** 77

541 atcgtctttgtggtgatatgtgtcccgctgacatcagtgagctacggtatgacttactgg

**I V F V V I C V P L T S V S Y G M T Y W** 97

601 ccatttggacagttcttctgcaagttgaacgcctacatgcaatgcgtgtccgttcaagct

**P F G Q F F C K L N A Y M Q C V S V Q A** 117

663 acgtgtacaacacttaccgccatgacagtggatagatattacgtcatcatgtcgccatta

**T C T T L T A M T V D R Y Y V I M S P L** 137

723 gcatcaaggcgcacgcgcaccatatgcagagctggtatggtgtgtgcgtccatttggata

**A S R R T R T I C R A G M V C A S I W I** 157

783 ttttcagctgttgttcacattccagtagctgttttcttcaagatagaggtcatcaattgg

**F S A V V H I P V A V F F K I E V I N W** 177

843 tttggtgagatcaacgagtactgtaagtttaccctgcacaagcctgcggctctctccggc

**F G E I N E Y C K F T L H K P A A L S G** 197

903 tacttcatctatctgagtctatccacgttccttttacccctcacgatcattgccgtgtgt

**Y F I Y L S L S T F L L P L T I I A V C** 217

963 tatagcctcatattatcacatctgtggagtctaagccgggtcggtagatgccgggagccc

**Y S L I L S H L W S L S R V G R C R E P** 237

1023 acgtcgactgccgggtacgacccgctgtcgcagcagagtcgctctggtgcccttcccccg

**T S T A G Y D P L S Q Q S R S G A L P P** 257

1083 gcacaaagctcggcccggacggccagcaaaacgtggaaaaccacccggatcgtgctgtgt

**A Q S S A R T A S K T W K T T R I V L C** 277

1143 gttgttatgctttttgccgtttgctgggcaccccttcaggcgttcaacgtgtggaatgca

**V V M L F A V C W A P L Q A F N V W N A** 297

1203 atcgaccccgagcaccacagctcgtcggttcacgtaaataatctacgagtgttctgcctg

**I D P E H H S S S V H V N N L R V F C L** 317

1263 tgtcttgcgtatgcaaactcttgcatcaaccctatcgtttacgccctcgcggggaccagt

**C L A Y A N S C I N P I V Y A L A G T S** 337

1323 tatcggcaccatctccatcagatggtgtcaggaagcaacaagagcgggcgcatcaatagc

**Y R H H L H Q M V S G S N K S G R I N S** 357

1383 aggttctcaccaacgggaggtagaaattcctcatacagatcggttcgtcggatgtctcga

**R F S P T G G R N S S Y R S V R R M S R** 377

1443 accgaagctacatctgtaagtacttgtgtttgaaacacacgaatgtgactttgaaagtgg

**T E A T S V S T C V *** 387

1503 ctcagaacaaacggtgtatcacacttgtgtgttcaaaacccatctgccccttgacatgtc

1563 aatttggatactcacccttatctatcaccgtttacacgtagataggggaggcttatcccc

1623 tcactggatcgtacgctaattgctgtttatatggatgtagcttttgtgaatgtagttttt

1683 tctcccggaacaaattggaagagctcattcgtttaaccatttaacagccgactgcatact

1743 aacacctgcatccggggcagtggagtccgggacaaggtatttgaacgacttgggatggaa

1803 ggatacttcacagtttgccgtatttgcagtgcattttgctgacaaatattcaaacagttg

1863 tgttcaccctagactagtgtagaaacgattgtaaatccaaggctaatagatgacatgata

1923 ggatacaaagcctcacgtccgtattgttacaaactggtctaagcagtaggtcctaacagt

1983 tcaaacaaagtctttccgttggctgagagacgattatggtaaagcggtccgtattgatgt

2043 attttaaaggcaaggtacacgttccgtaaatgtgaaagaccagtgttctcatctggtgta

2103 tccaataataagcataaaataacaggcttgtgaa

**H. *Asterias rubens* kisspeptin receptor 8 (ArKPR8).** The transcript sequence (lowercase, numbering on the left) and the deduced amino acid sequence (uppercase; numbering on the right) are shown. This transcript was identified by analysis of *A. rubens* radial nerve cord transcriptome sequence data (contig 1118377). The GenBank accession numbers for these transcript and protein sequences are MT358426.1 and QVN25225.1, respectively. The sequences have also been confirmed by genome sequencing (XM_033778687.1, XP_033634578.1) but with polymorphisms, the positions of which are highlighted here in red.

1 cttgaattatttgacttgcgatacacgttaccactctggacaagagacagtgtttcccca

61 taaagaagcgtttcctcttcattatgttaacttgtgcgctcaattaggccccgtccgtac

121 gcggcagttcatcgtgaggcgaagacgcgcattgttggccgtcggactgttgctgggatc

181 ccctcgtctagtaacccatgagctcagccaggtgtgaggactacagaggcagagcggatg

241 gggctcaaactgatcatcacctcattactctgttaaactctacggagtgggaaaagtaca

301 ccttctattccaatgacttgcataaggtgtaggggttaacaatccgcaatacaacacccc

361 tgctggtgtattgagttatcttcattccaccgtctgcgcaactaaatccctcctctcagc

421 atttttttttttattacgcgattgggacgacgacgacgcaagaaaaatcattatacgccg

481 tgtgtttatcgctctgggactgctgcagcagcagatgccaccatcattacaagcaagatg

**M**  1

541 gagtaccctgatgtatcttattcctactcttacagcagtttctactactcatccaattca

**E Y P D V S Y S Y S Y S S F Y Y S S N S** 21

601 aacttcaccaatgggttcgaaggagagacgggtgttcacgctatactagttccgatcatc

**N F T N G F E G E T G V H A I L V P I I** 41

661 tttgggataattaccgtggtcgggctgattggtaacggctgtgtggtgatagtcattgcc

**F G I I T V V G L I G N G C V V I V I A** 61

721 cggaaccgatgcatgaggactgtcactaatttcttcataatgaataatgccatcacggat

**R N R C M R T V T N F F I M N N A I T D** 81

781 atggtgtttgtggtaatctgtgcacccgttacggcgtcgcagtttatactcaccgattgg

**M V F V V I C A P V T A S Q F I L T D W** 101

841 atttttggtgacttcatatgtaagctggtggtcttcatgcagtatgtgtcagtgcaagcc

**I F G D F I C K L V V F M Q Y V S V Q A** 121

901 tcatgttccaccatcatggcaatgacaatagaccgttacatggtcatacttcaccctatg

**S C S T I M A M T I D R Y M V I L H P M** 141

961 cggtcacttcatgctaggacgatacgccggactacatgtataaacatcgtcatatggcta

**R S L H A R T I R R T T C I N I V I W L** 161

1021 acttcgtttttacttcacgtgccggtggcgatttactacgaacaagtgtccgagccaaac

**T S F L L H V P V A I Y Y E Q V S E P N** 181

1081 aacggctacttctgcggcacgaactttgtgaacatcacggccagtaaaatctaccatttc

**N G Y F C G T N F V N I T A S K I Y H F** 201

1141 tacgccgtcatcgttctctacgtgtttccgttcctcgtcatgacattttgctacctccgg

**Y A V I V L Y V F P F L V M T F C Y L R** 221

1201 attctgcgcaaggtgtggagtaagtacctcatcgtgacgtcatccacgcagacgcagagg

**I L R K V W S K Y L I V T S S T Q T Q R** 241

1261 aagcgcaggtggaagataacgcgcatgactcttctggtcgtcattctcttcggcatctgc

**K R R W K I T R M T L L V V I L F G I C** 261

1321 tgggggccgatccacgccgtacacttggtggcgctgttcaagacggccaccagcgcgact

**W G P I H A V H L V A L F K T A T S A T** 281

1381 gagattgactggtcgtactataacttctccatattctgcctgtgtatctcatactcgaac

**E I D W S Y Y N F S I F C L C I S Y S N** 301

1441 tctgccattaaccctttcgtgtacgctttcagcggtcggagttatcgctctctgttgcta

**S A I N P F V Y A F S G R S Y R S L L L** 321

1501 tcctgtttgaaacggaaaccagggaagaaccccgtgagtcgaatgtgcagcagtaggacc

**S C L K R K P G K N P V S R M C S S R T** 341

1561 aaagcaggatcggcacagtactctttggaaatgaacaacctcattcgaaccaatggtcag

**K A G S A Q Y S L E M N N L I R T N G Q** 361

1621 aatgggagatcgccaaagacgaggtatacgaaccagtatatcggtggagggcgccatatt

**N G R S P K T R Y T N Q Y I G G G R H I** 381

1681 gtcagagctgtaccggaataagggagcaaattctactgttctatttatagctgaaagaca

**V R A V P E *** 387

1741 ctgctgaactccattgggtggagcactgaaccacttattcactttgtagaccggttaaga

1801 attcattattgctttaaaaatagtgaataatttgttatggaaaagggtattgatgtaaca

1861 gaacattttatattatgcctatttttgcctatcatctctgtctaaatttgtcggtctgct

1921 cgttttcttcattataagtcctaccgacctatacaaattatcttgaaaaaaattagtatt

1981 tttcaaaacatgtacagtaaaacctcatctcgtccaccctcgaattctgtttctgcttgt

2041 atttccatttcatctcttgctgggtacactttattattattattatt

**I. *Asterias rubens* kisspeptin receptor 9 (ArKPR9).** The transcript sequence (lowercase, numbering on the left) and the deduced amino acid sequence (uppercase; numbering on the right) are shown. This transcript was identified by analysis of *A. rubens* radial nerve cord transcriptome sequence data (contig 1117923). The GenBank accession numbers for these transcript and protein sequences are MT358427.1 and QVN25226.1, respectively. The sequences have also been confirmed by genome sequencing (XM_033778818.1, XP_033634709.1). However, the predicted start codon for XP_033634709.1 corresponds to the methionine residue at position 127 in this sequence.

1 acctttacatgcaatacattcttagcggagcagtagacgggttacacgtcaggaatcaatc

62 tagaacgtatactcaagtcttggatttagtcccgagtggtggacctttacatgcaataca

122 ttcttggcggggaagaggagaaagctcaacaccatctataagctagcaagctgaggaaat

182 attaaactgtgagatgcgacttcgattaaagtgatctaaaaacctcttgcgataatgtga

242 acgagttgatggatgtgtaagacgtgcacgaatgcaggtatctcgcccagccagttgggc

302 cttggaaatactattggttaaccaggagcggacgctaaagcctactaagcagtttgatca

362 attggagacaggcatttccaggtcgtcaagtgagcacaagggtgtctgcttcaaccggcc

422 aataaaagggtactccatgtatgcacgctggccacacacgggcttaagagtatgctgcct

**M L P**  3

482 aacgtcacacacaacactaccatgcatatatccgacgaggtattcggccaattggtcatg

**N V T H N T T M H I S D E V F G Q L V M** 23

542 gtgctcttaacggtgctcatatcggtggtgtcagcggtgggaattttcggaaacgcgctg

**V L L T V L I S V V S A V G I F G N A L** 43

602 gtcatgtttatcgtgttctgctacaaggacatgcacaccgtcatcaactactcgttcgct

**V M F I V F C Y K D M H T V I N Y S F A** 63

662 aacctagctctgactgacctgacccttttgctcctggacggcttgcctactgctgcggat

**N L A L T D L T L L L L D G L P T A A D** 83

722 actatggggacaaacttctcggctagtctaggatgcaatatcccaatatacctgcaatat

**T M G T N F S A S L G C N I P I Y L Q Y** 103

782 gttaccgctgaagtaacgagcttgaccctcgcatttctatcctacgaccgttaccagctc

**V T A E V T S L T L A F L S Y D R Y Q L** 123

842 atcgttcaccctataaagtcactaagcagacggtctccaaagacaatactgaaaatgttc

**I V H P I K S L S R R S P K T I L K M F** 143

902 gccggaatatggctagtttctttcttaatacaggttcccgtcgcccttgtcgctggcccg

**A G I W L V S F L I Q V P V A L V A G P** 163

962 acagcatcgggttcgtgttctgagttctacctctccagcggggagcagtatttcttcgcc

**T A S G S C S E F Y L S S G E Q Y F F A** 183

1022 ttcgagacggcctccctgtactgcatccctttagccatcattatcttctgctacgccacg

**F E T A S L Y C I P L A I I I F C Y A T** 203

1082 atgggccgaaagatgctagccgcggggccacggagcagcatacggcgcaggcgcagtgtg

**M G R K M L A A G P R S S I R R R R S V** 223

1142 aacagtatcctcttcgtcatcatcctgtacgtcctctgctggttgccggtccacgttgtt

**N S I L F V I I L Y V L C W L P V H V V** 243

1202 cacctctggatggcctttgaccccgaggtcgaaccgactgacgcgctctacaccgaactg

**H L W M A F D P E V E P T D A L Y T E L** 263

1262 cacaccgccgccaacgtcctgttgtttatgaacagcagcgtgaacccgtttgtgtacacc

**H T A A N V L L F M N S S V N P F V Y T** 283

1322 cttggggggccttcataccggaggcacattaaaacgctggcgatatcaatcgtcaaatgc

**L G G P S Y R R H I K T L A I S I V K C** 303

1382 aggttcaaccaaccggaccctcgtcgccagcagagtatatcaagaagaagtggagctgag

**R F N Q P D P R R Q Q S I S R R S G A E** 323

1442 agatcgtctgatgcatatgttgtgaacaacagaccgaaaccatcacctgcaccctcaaca

**R S S D A Y V V N N R P K P S P A P S T** 343

1502 aatgaaccggtagaaccagggagtatacaatgttcttcaacgtggcta**tga**gctcacttg

**N E P V E P G S I Q C S S T W L ***  359

1562 tacacataggatagtgacactacaaatctttcatttttttgcactgatatcttttgtatc

1622 cacttattacggaaaccacttgaggcctatggtatgaacaggcgacaaaactttcgagct

1682 gaactgctttgcaccacacacaggcacgtacacttcgattttgaaagggcaagagggcac

1742 cagtgcattttctcctcggtaagggccggtcatacgaggaaattgtgtgtttcaactgga

1802 acatttttagggcaccaaggcaattaccagggagcatggaggcaatcgccttcgttgcct

1862 ccgtgaagtactatcaggtctgttcaccctcttgcacgtatgcactagagcctacagtct

1922 gactgggacattcacctctcgtttgctaaagggcagggcaccaaggcgtgttcgttgcac

1982 ttggctccgtgaccactctcttgcatgtatgcactaaatgcctatagtctgactgagaca

2042 tccacttctcgtgtttgaaagggcaagggcgccaaggcatgttcgttgcctctgtgaagt

2102 atcacggctgactaccctcttgccatttatatgcacaaaggcctatatagtctgacttga

2162 gacatccacctctcgcacgggcgttttcgaagttaagctcttaacaatgtaatcatacgg

2222 gaagtaaaaacatggtgcactgggacactttcacggttgcagacgttaaattgaccacat

2282 gatatcgagaaattggttttctcaaggagctcttatggtaacgtctgattgcactagatc

2342 aaattaccagcatcttacttagaaaccttggggtatatataacctatatgatggataata

2402 ataatagcaacgccacgaggcaagactaggcacgcgaaacacgttagttttacatcaaac

2462 ttacagaaagagactttgatacacgttgtattttcgcactgatattaacattgtgttcac

2522 agaagtgacgtaaacaagtgacgttggttatataataagtacggacgacagggttttgga

2582 gaagtttgaaattactgtaaaataattatttaatctggttattgactcttttataatcaa

2642 gttcggtatgaagatgacaacacatacagtgatcaattttttacgtaaaactttttcgac

2702 cacacagtattaatttttttaacatatagtacacacgaaagactgtggaagtctttttca

2762 ggcaaaacaaataaaacaagggaagaggaaaaggaagctattccatacatagggaacatg

2822 ttctaaatttacgggttactattccgtacgtaccaattcgcacgtattaatgtttttgtc

2882 actatacacgtttacgtattaaaaaatacactgtcataaaaaaaacgaaaagacacgcac

2942 actagtgaatatacgtatgtacgatttagtaataattatttatgtacgtaaaatcttctt

3002 tttgttatgtgcacttcagaaaaatattctcttcaacacttgtaaaaacaccgtgtcgac

3062 gtgtatgtaagcccataattttatagtgtttaaaaaccctttcttgggtgatgtacttct

3122 tttgttttaaaggcagtggacactattggtaattactcaaaatgattattggca

**J. *Asterias rubens* kisspeptin receptor 10 (ArKPR10).** The transcript sequence (lowercase, numbering on the left) and the deduced amino acid sequence (uppercase; numbering on the right) are shown. This transcript was identified by analysis of *A. rubens* genome sequence data (XM_033769619.1, XP_033625510.1, nucleotides 533 - 3176) and *A. rubens* radial nerve cord transcriptome sequence data (contig 1081437, nucleotides 1 - 806; contig 1033161, nucleotides 835 - 1306).

1 gtgaactacaggtgaaatagtgtgtatagctcaacatcaaccatggcaaacactggagtc

**M A N T G V**  6

61 gtctcggctacgcagttgataacagaacaaacaagtagtgcagcgttgatgacaaccatg

**V S A T Q L I T E Q T S S A A L M T T M**  26

121 cttctaccgttcatgaacgactcatcagcttggaacggcacggggaacggaactgacgaa

**L L P F M N D S S A W N G T G N G T D E**  46

201 gatgtcgattcttatgttgacggttcggacgacggacgcgaattcggccccatacagatc

**D V D S Y V D G S D D G R E F G P I Q I**  66

261 atcgccgtcaccatgtttgttgtcattttcgtcggcctgatcggaaacggtgccgtcata

**I A V T M F V V I F V G L I G N G A V I**  86

341 tacatcgtggtacgtcacaaggacatgcacaccacgacaaacttctccttcgctaacctg

**Y I V V R H K D M H T T T N F S F A N L**  106

401 gcagtgacagactttctgtttctgcttgttcacgccctgactacgtctattgataacatt

**A V T D F L F L L V H A L T T S I D N I**  126

461 ggctttaatctatcattgacaatcaactgttggcccaccttctacttgcgatatgttgta

**G F N L S L T I N C W P T F Y L R Y V V**  146

521 gctgaggtcacttgtttgacattggctctgatgtcatatgacagatatagaatggttgct

**A E V T C L T L A L M S Y D R Y R M V A**  166

581 catccattggacagcataagacataccaaacgacgtaagaatggtatcctcgtttgtatc

**H P L D S I R H T K R R K N G I L V C I**  186

641 ctgttcatatggattatttcgttggctttattttcacccgtcctcatctacaccactaag

**L F I W I I S L A L F S P V L I Y T T K**  206

701 tactatgggttgttctgcaccatcatattcccttgggcttacggccagaaagtctttcac

**Y Y G L F C T I I F P W A Y G Q K V F H**  226

761 acctcagctgccttcgtcatgtacgtcattccactctcattcatcgtcatgtgctacctg

**T S A A F V M Y V I P L S F I V M C Y L**  246

821 atgattttcctcaaactcagaaagcccaatcccggggaagtaggaataggtcaaaggtca

**M I F L K L R K P N P G E V G I G Q R S**  266

881 cggcgcatgcgcaagactctccgcttgattctggtcgtggtggttgtgtttgcactgtcg

**R R M R K T L R L I L V V V V V F A L S**  286

941 tggctgttagaacataccttcatcgtctgggtcgtatgggacccaaatttcctcccccaa

**W L L E H T F I V W V V W D P N F L P Q**  306

1001 tcgaaactttttactaccacaacggctaccactaaagtcattctgtatgtgaatagtgcc

**S K L F T T T T A T T K V I L Y V N S A**  326

1061 gccaacccgttcatgtatccctttgctgggactggttttaccaagcatctcgggtgttgt

**A N P F M Y P F A G T G F T K H L G C C**  346

1121 aaacggaggggccttagtagaaacaacactatggcaactagtacattcagttgtggatct

**K R R G L S R N N T M A T S T F S C G S**  366

1181 acaatgaagttatctgtggtacaatctaccgtcagcacatcggtacagagtttagatagc

**T M K L S V V Q S T V S T S V Q S L D S** 386

1241 attaatgtcagtgtccccgcaacagggtatcaaaaccatgggcaggtgtgaacaatagag

**I N V S V P A T G Y Q N H G Q V *** 402

**K. *Asterias rubens* kisspeptin receptor 11 (ArKPR11).** The transcript sequence (lowercase, numbering on the left) and the deduced amino acid sequence (uppercase; numbering on the right) are shown. This transcript was identified by analysis of *A. rubens* genome sequence data (XM_033778369.1, XP_033634260.1) and *A. rubens* radial nerve cord transcriptome sequence data (contig 1086882, nucleotides 1 - 907; contig 876302 nucleotides 895 - 1103; contig 567718 nucleotides 1086 to 1257).
