# Supplementary figures and images for: Receptor deorphanization in an echinoderm reveals kisspeptin evolution and relationship with SALMFamide neuropeptides
Source: BMC Biol. 2022 Aug 24;20:187. doi: 10.1186/s12915-022-01387-z (PMC9400282; doi:10.1186/s12915-022-01387-z)

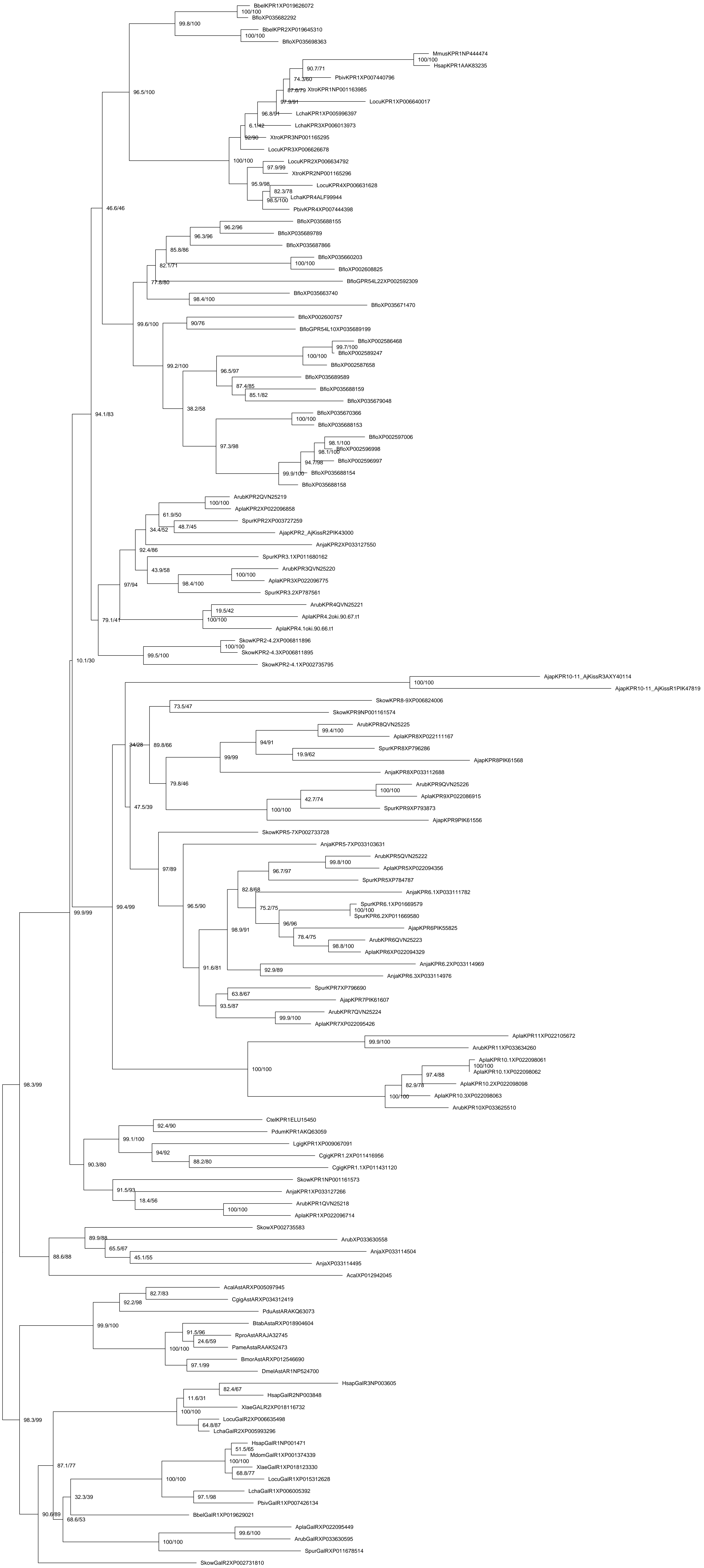

Supplement: Supplementary file 4 — Additional file 4. Phylogenetic analysis of bilaterian kisspeptin-type receptors, including expanded receptor families in the starfish A. rubens (ArubKPR1-11), other echinoderms, and Branchiostoma floridae. The phylogenetic tree was constructed using the maximum-likelihood method, LG+G4 amino-acid substitution model, and rooted with galanin/allatostatin-A-type receptors as an outgroup. Boostrap support for each node is stated according to SH-aLRT/UFBoot methods. The scale bar indicates amino acid substitutions per site. Species names are as follows: Apla, Acanthaster planci; Ajap, Apostichopus japonicus; Arub, Asterias rubens, Anjap Annessia japonica; Bbel, Branchiostoma belcheri; Bflo B. floridae; Ctel, Capitella teleta; Cgig, Crassostrea gigas; Hsap, Homo sapines; Lcha, Latimeria chalumnae; Locu, Lepisosteus oculatus; Lgig, Lottia gigantea; Mmus, Mus musculus; Pdum, Platynereis dumerilii; Pbiv, Python bivittatus; Skow, Saccoglossus kowalevskii; Spur, Strongylocentrotus purpuratus; Xlae Xenopus laevis; Xtro, X. tropicalis. Accession numbers for the sequences of the receptors included in this tree are listed in additional file 3. [file 12915_2022_1387_MOESM4_ESM.pdf]

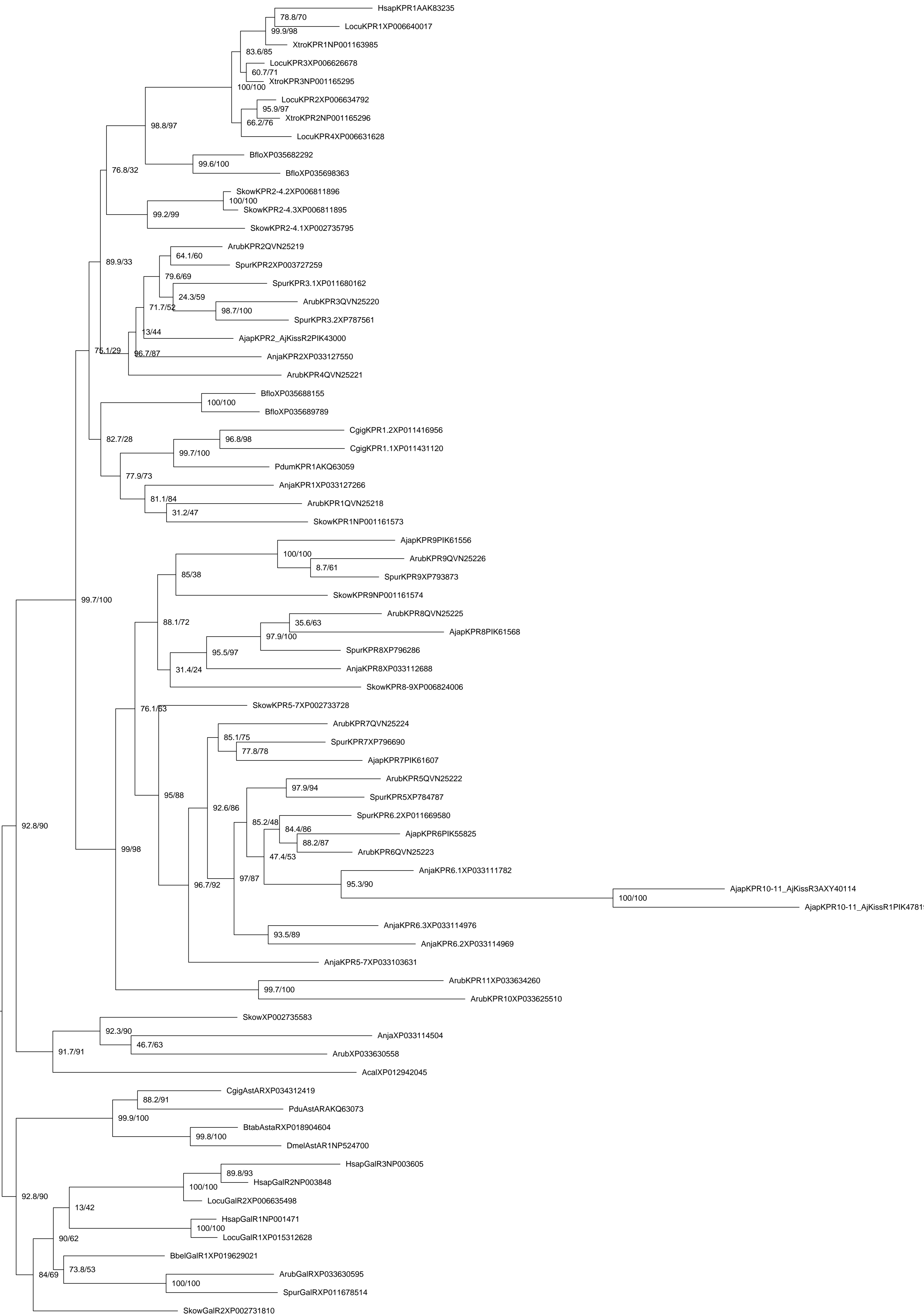

Supplement: Supplementary file 5 — Additional file 5. Phylogenetic analysis of bilaterian kisspeptin-type receptors, including expanded receptor families in the starfish A. rubens (ArubKPR1-11) and other echinoderms. The phylogenetic tree was constructed using the maximum-likelihood method, WAG+G4 amino-acid substitution model, and rooted with galanin/allatostatin-A-type receptors as an outgroup. Boostrap support for each node is stated according to SH-aLRT/UFBoot methods. The scale bar indicates amino acid substitutions per site. Species names are as follows: Ajap, Apostichopus japonicus; Arub, Asterias rubens, Anjap Annessia japonica; Bflo, Branchiostoma floridae; Cgig, Crassostrea gigas; Hsap, Homo sapines; Locu, Lepisosteus oculatus; Pdum, Platynereis dumerilii; Skow, Saccoglossus kowalevskii; Spur, Strongylocentrotus purpuratus; Xtro Xenopus tropicalis. Accession numbers for the sequences of the receptors included in this tree are listed in additional file 3. [file 12915_2022_1387_MOESM5_ESM.pdf]

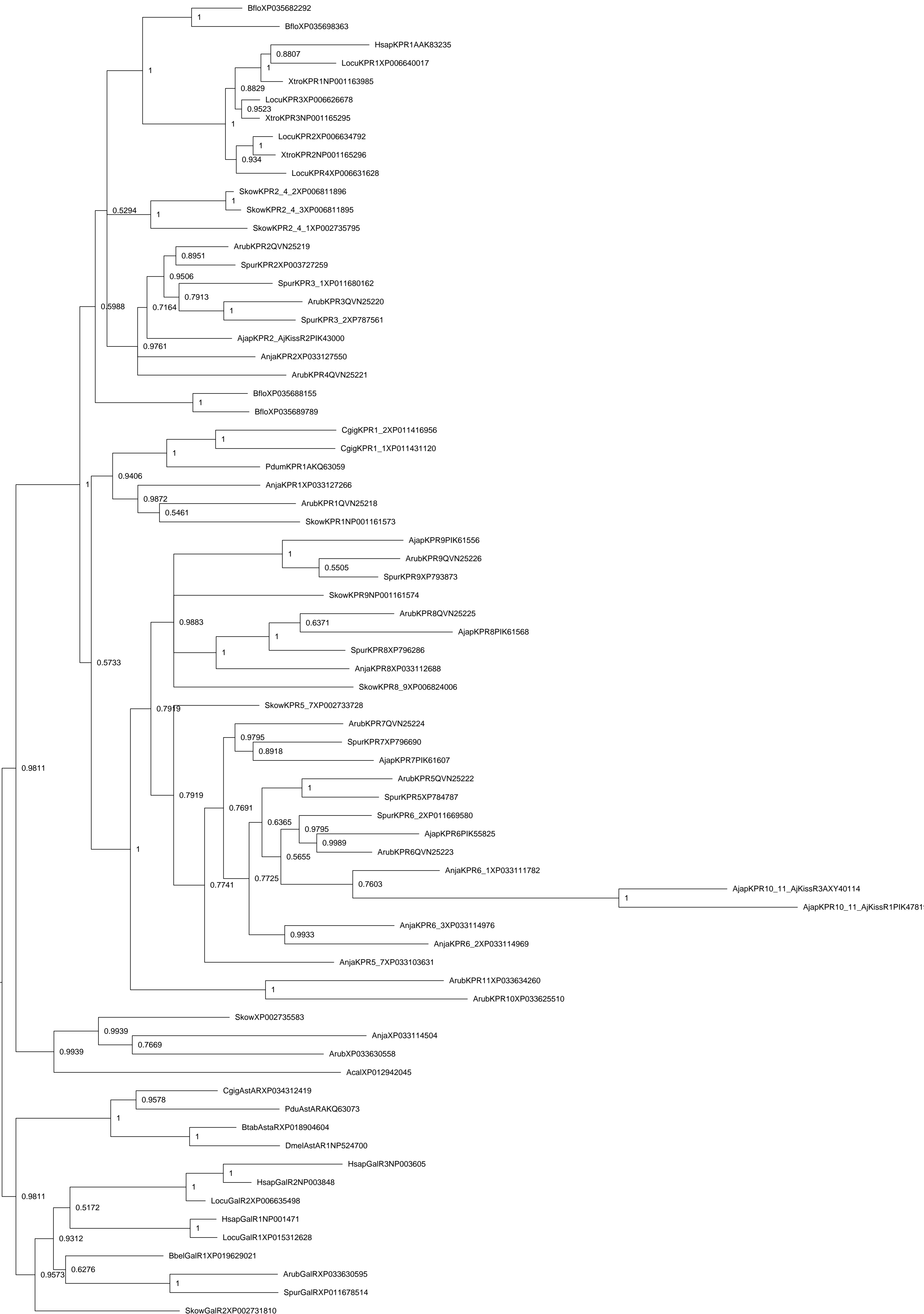

Supplement: Supplementary file 6 — Additional file 6. Phylogenetic analysis of bilaterian kisspeptin-type receptors, including expanded receptor families in the starfish A. rubens (ArubKPR1-11) and other echinoderms. The phylogenetic tree was constructed using the Bayesian method, WAG+G4 amino-acid substitution model, and rooted with galanin/allatostatin-A-type receptors as an outgroup. Probabilities for nodes are stated according to SH-aLRT/UFBoot methods. The scale bar indicates amino acid substitutions per site. Species names are as follows: Ajap, Apostichopus japonicus; Arub, Asterias rubens, Anjap Annessia japonica; Bflo, Branchiostoma floridae; Cgig, Crassostrea gigas; Hsap, Homo sapines; Locu, Lepisosteus oculatus; Pdum, Platynereis dumerilii; Skow, Saccoglossus kowalevskii; Spur, Strongylocentrotus purpuratus; Xtro Xenopus tropicalis. Accession numbers for the sequences of the receptors included in this tree are listed in additional file 3. [file 12915_2022_1387_MOESM6_ESM.pdf]

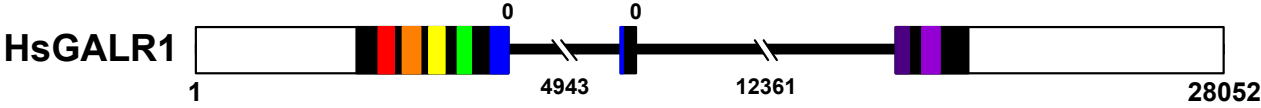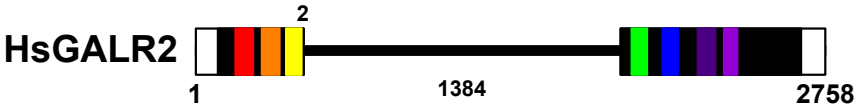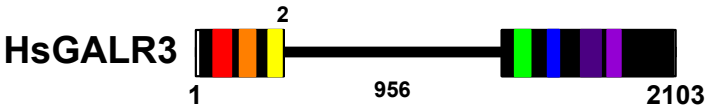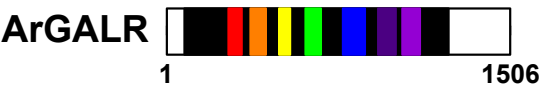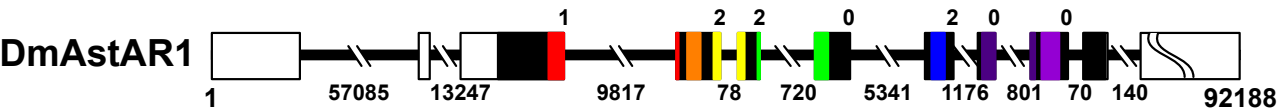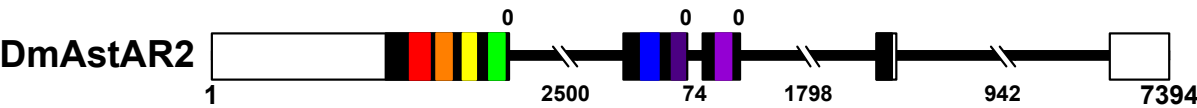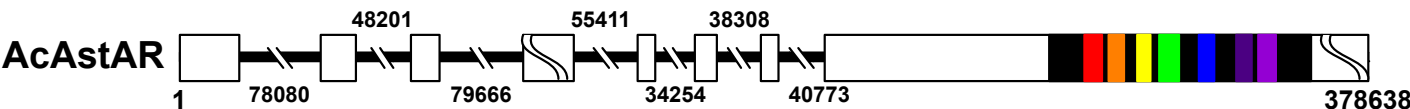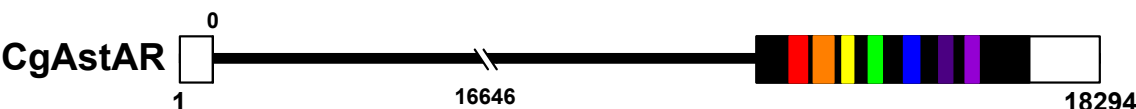

Supplement: Supplementary file 10 — Additional file 10. Comparative analysis of the structure of genes encoding galanin-type receptors and allatostatin-A-type receptors. The exon/intron structure of genes encoding galanin/allatostatin A-type receptors from Homo sapiens, the starfish A. rubens, and three protostome invertebrate species are shown. Exons are shown as rectangles, with non-coding regions white and protein-coding regions black or colored (regions encoding predicted transmembrane domains 1-7 are shown in red, orange, yellow, green, blue, dark purple and light purple, respectively). Introns are shown as lines, with intron length (bases) stated underneath. Intron phase is stated above the line at the start of introns that interrupt coding exons. Species names are as follows: Hs (Homo sapiens), Ar (A. rubens), Dm (Drosophila melanogaster), Ac (Aplysia californica) and Cg (Crassostrea gigas). A list of ID numbers are shown in additional file 9. [file 12915_2022_1387_MOESM10_ESM.pdf]

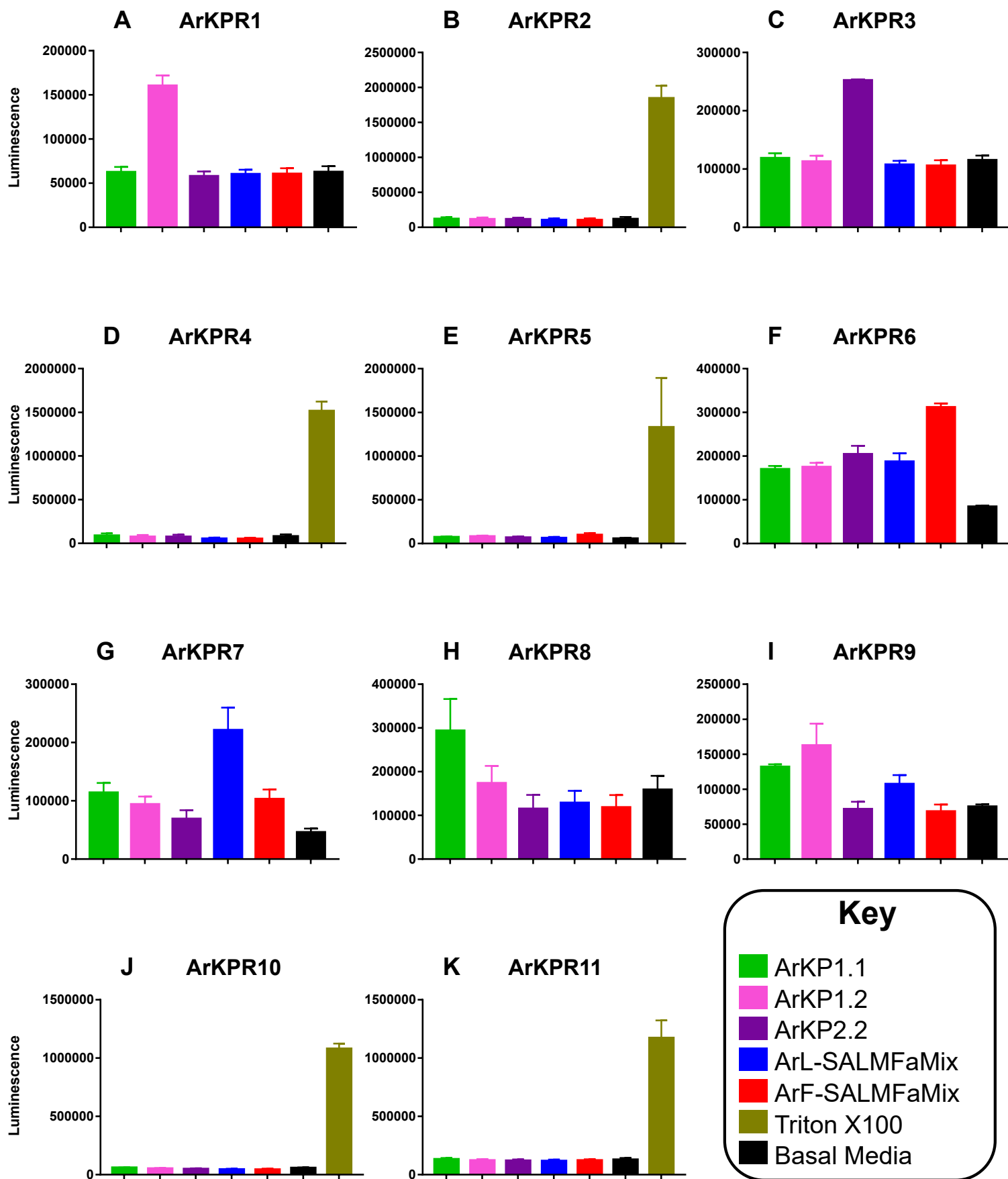

Supplement: Supplementary file 14 — Additional file 14. Comparison of luminescence measurements of CHO-K1 cells transfected with the A. rubens kisspeptin-type receptors ArKPR1-11 at 35 seconds after exposure to candidate peptide ligands (10-5 M). Each bar represents mean values (± S.E.M.) from at least two independent experiments, with each experiment performed in triplicate. ArKP1.1 (green), ArKP1.2 (pink), ArKP2.2 (purple), ‘cocktail’ of L-type SALMFamide precursor derived peptides ArS1.1-7 (blue), ‘cocktail’ of F-type SALMFamide precursor derived peptides ArS2.1-8 (red) and assay media as a negative control (black). Triton X-100 (olive green), which triggers luminescence via receptor-independent mechanisms, was tested as a positive control to check for cell viability. The source data for these experiments are provided in additional file 15. [file 12915_2022_1387_MOESM14_ESM.pdf]

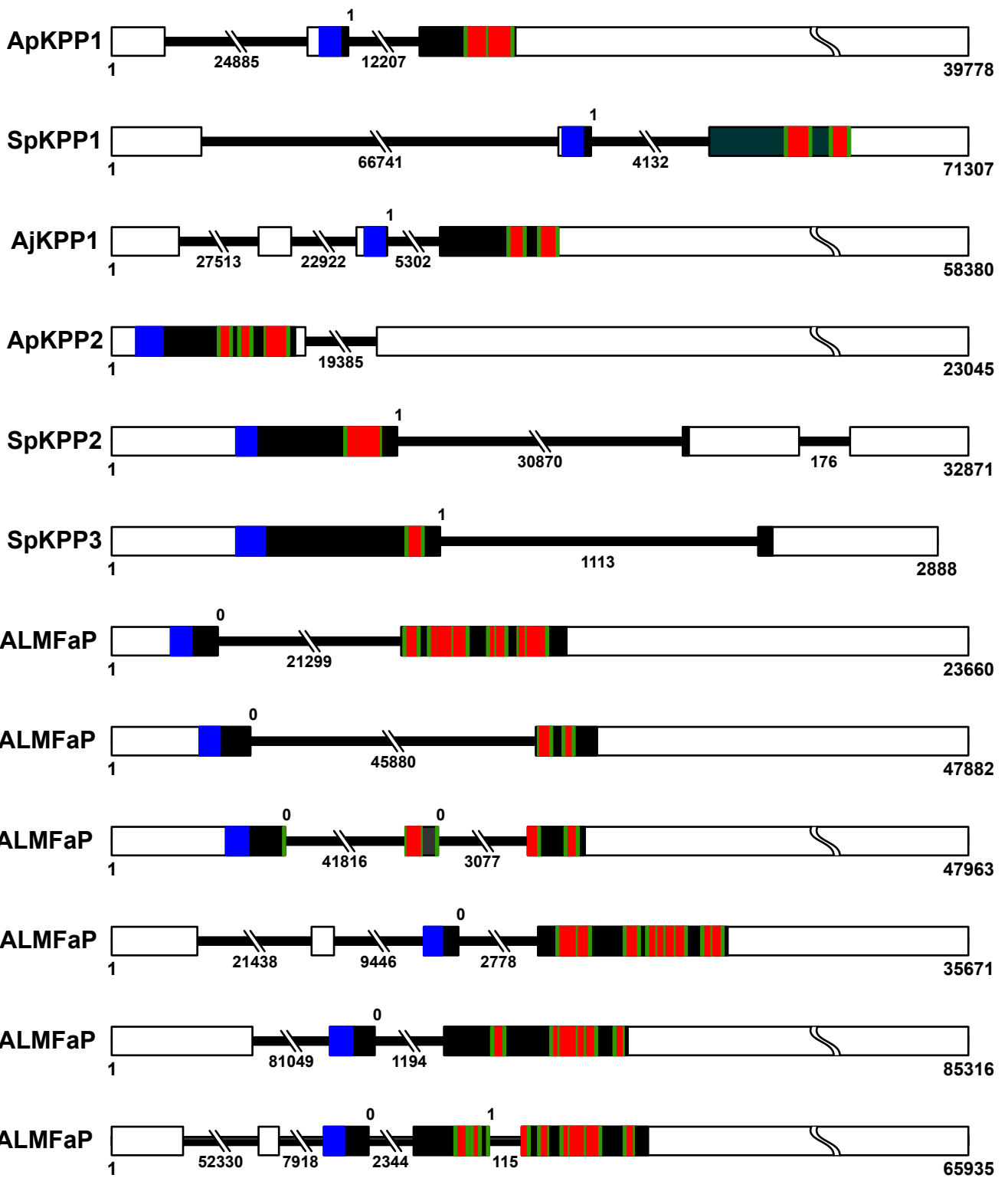

Supplement: Supplementary file 16 — Additional file 16. Structure of genes encoding precursors of candidate ligands for kisspeptin-type receptors in other echinoderms. Comparison of the exon/intron structure of genes encoding precursors of candidate ligands for kisspeptin-type receptors in echinoderms and genes encoding kisspeptin-type precursors in three vertebrate species. Exons are shown as rectangles, with non-coding regions white and protein-coding regions black or colored (regions of exons encoding the N-terminal signal peptide, neuropeptides and predicted monobasic or dibasic cleavage sites are shown in blue, red, and green, respectively). Introns are shown as lines, with intron length (bases) stated underneath and intron phase stated above. Species names are abbreviated as follows: Acanthaster planci, Ap; Strongylocentrotus purpuratus, Sp; Apostichopus japonicus, Aj. The accession numbers for the sequences of the precursors shown in this figure are listed in additional file 17. [file 12915_2022_1387_MOESM16_ESM.pdf]

**A** ArKPR2

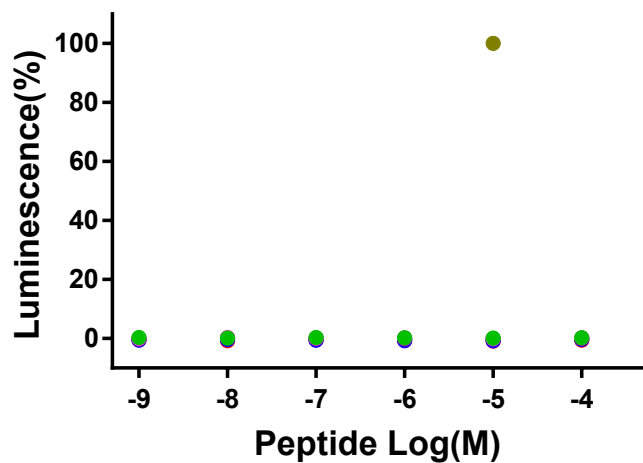

**B** ArKPR4

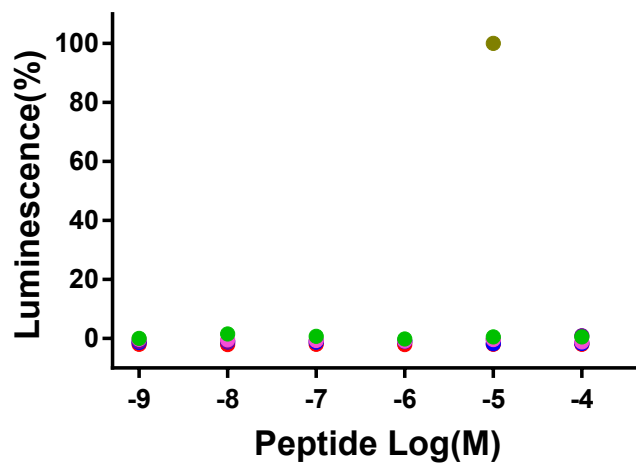

**C** ArKPR5

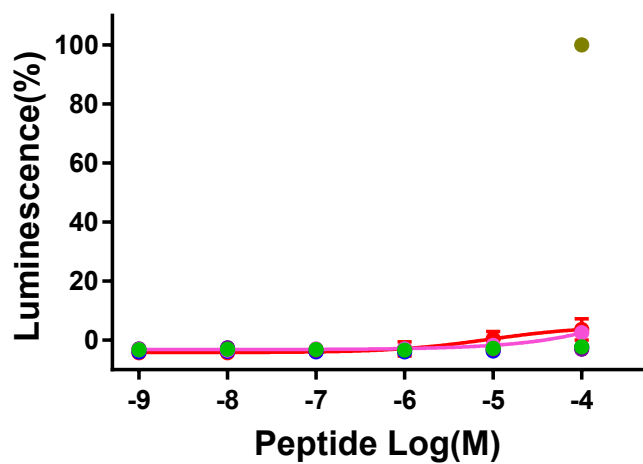

**D** ArKPR10

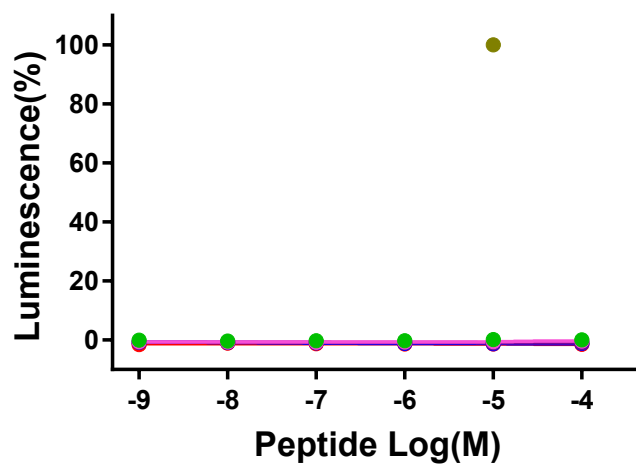

**E** ArKPR11

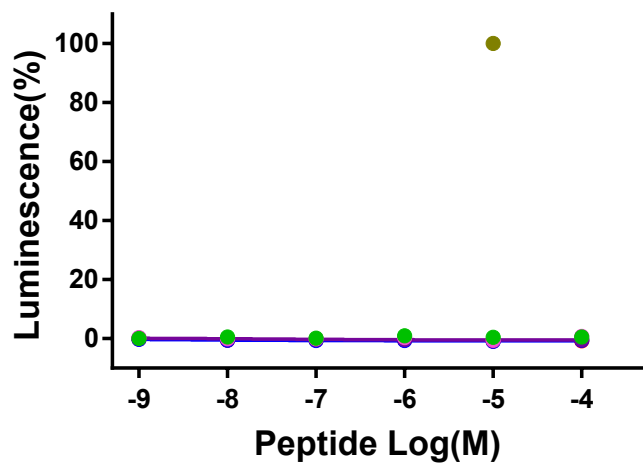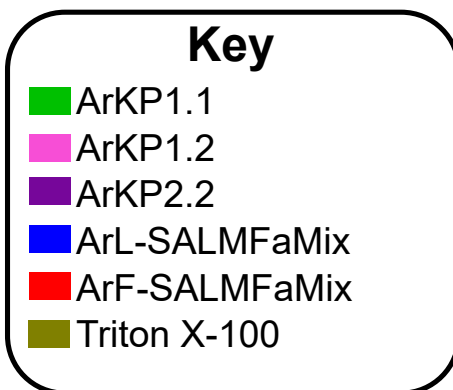

Supplement: Supplementary file 19 — Additional file 19. Concentration-response graphs for A. rubens kisspeptin-type receptors (ArKPR2,4,5,10,11) that were not activated by any of the candidate ligands tested. Key: Green = ArKP1.1, Pink = ArKP1.2, Purple = ArKP2.2; Blue = ‘cocktail’ of neuropeptides derived from the L-type SALMFamide precursor (ArS1.1-7); Red = ‘cocktail’ of neuropeptides derived from the F-type SALMFamide precursor (ArS2.1-8). Each point represents mean values (± S.E.M.) from at least four independent experiments, with each experiment performed in triplicate. Luminescence is expressed as a percentage of the maximal response observed in each experiment. Triton X-100 (olive green), which triggers luminescence via receptor-independent mechanisms, was tested as positive control to check for cell viability and the response to Triton X-100 in each experiment was assigned as 100% luminescence. The source data for these experiments are provided in additional file 15. [file 12915_2022_1387_MOESM19_ESM.pdf]

## A ArKPR6

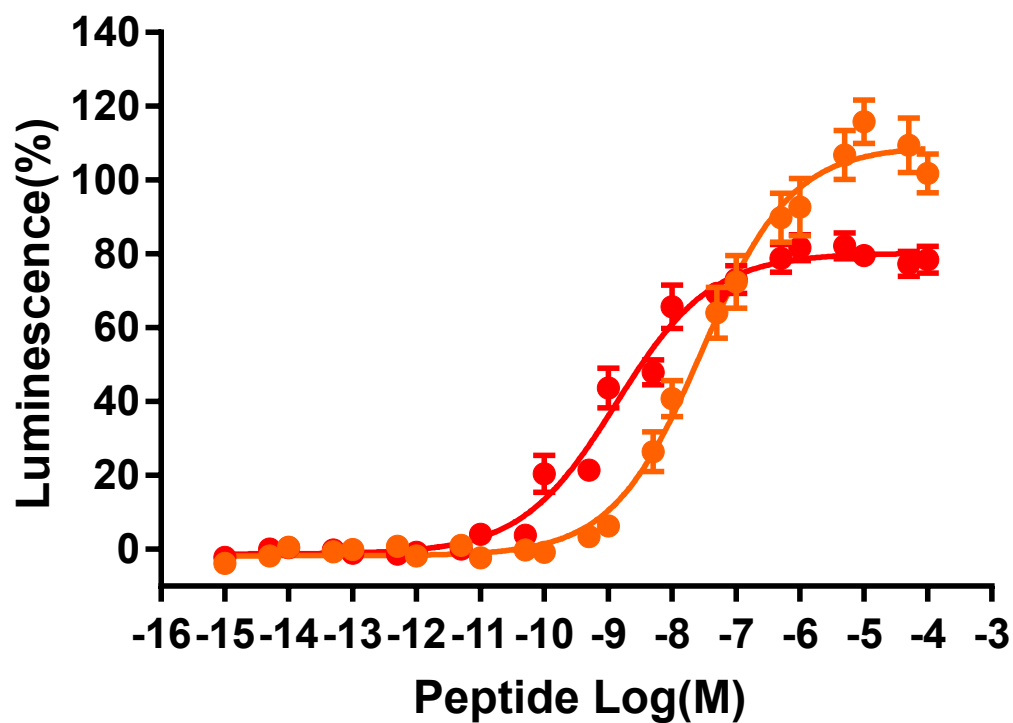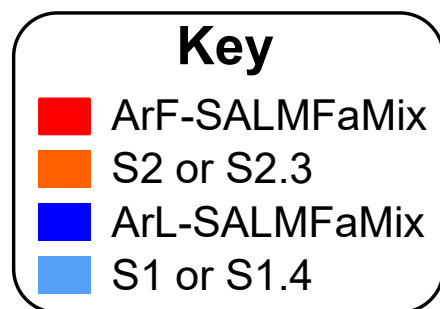

## B ArKPR7

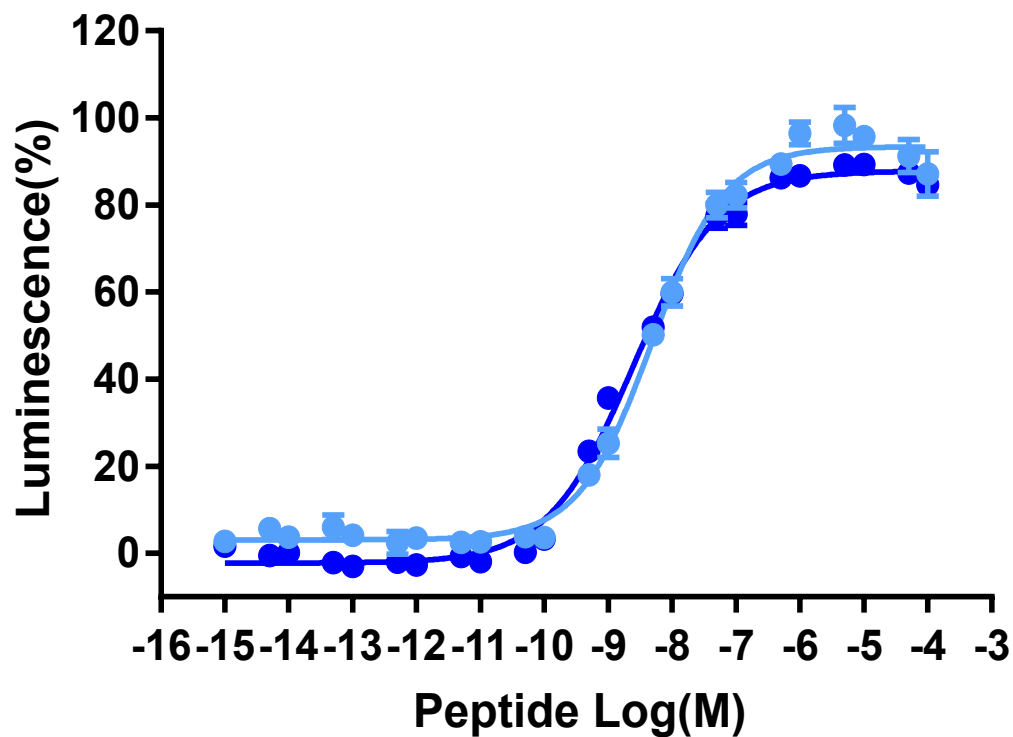

Supplement: Supplementary file 20 — Additional file 20. A. Concentration-response graph comparing the potency/efficacy of S2 (S2.3; EC50 = 2.93 x 10^-8^ M) and a ‘cocktail’ of neuropeptides derived from the F-type SALMFamide precursor (EC50 = 1.33 × 10^-9^ M) as ligands for ArKPR6. B. Concentration-response graph comparing the potency and efficacy of S1 (S1.4; EC50 = 4.79 x 10^-9^ M) and a ‘cocktail’ of neuropeptides derived from the L-type SALMFamide precursor (EC50 = 2.43 × 10^-9^ M) as ligands for ArKPR7. Each point represents mean values (± S.E.M.) from at least four independent experiments, with each experiment performed in triplicate. Luminescence is expressed as a percentage of the maximal response observed in each experiment. The source data for these experiments are provided in additional file 15. [file 12915_2022_1387_MOESM20_ESM.pdf]
