# Supplementary material for: Receptor deorphanization in an echinoderm reveals kisspeptin evolution and relationship with SALMFamide neuropeptides
Source: BMC Biol. 2022 Aug 24;20:187. doi: 10.1186/s12915-022-01387-z (PMC9400282; doi:10.1186/s12915-022-01387-z)
Supplement: Supplementary file 7 — Additional file 7. CLuster Analysis of Sequences (CLANS) of receptors identified by BLAST analysis of bilaterian proteomes using human and S. purpuratus kisspeptin-type receptors as queries. BLOSUM62 cluster map shows kisspeptin-type receptors, the closely related galanin/allatostatin-A-type receptors, and other neuropeptide receptor families: allatotropin/orexin, neuropeptide Y, tachykinin, somatostatin/allatostatin-C, CCHamide/gastrin-releasing peptide. Nodes are labelled with taxon-specific shapes and colors, as shown in the key. Connections represent BLAST relationships with a P value > 1e-40. All family names of receptors are labelled in the figure. The accession numbers for the receptors shown in this figure are provided in additional file 2. [file 12915_2022_1387_MOESM7_ESM.pdf]

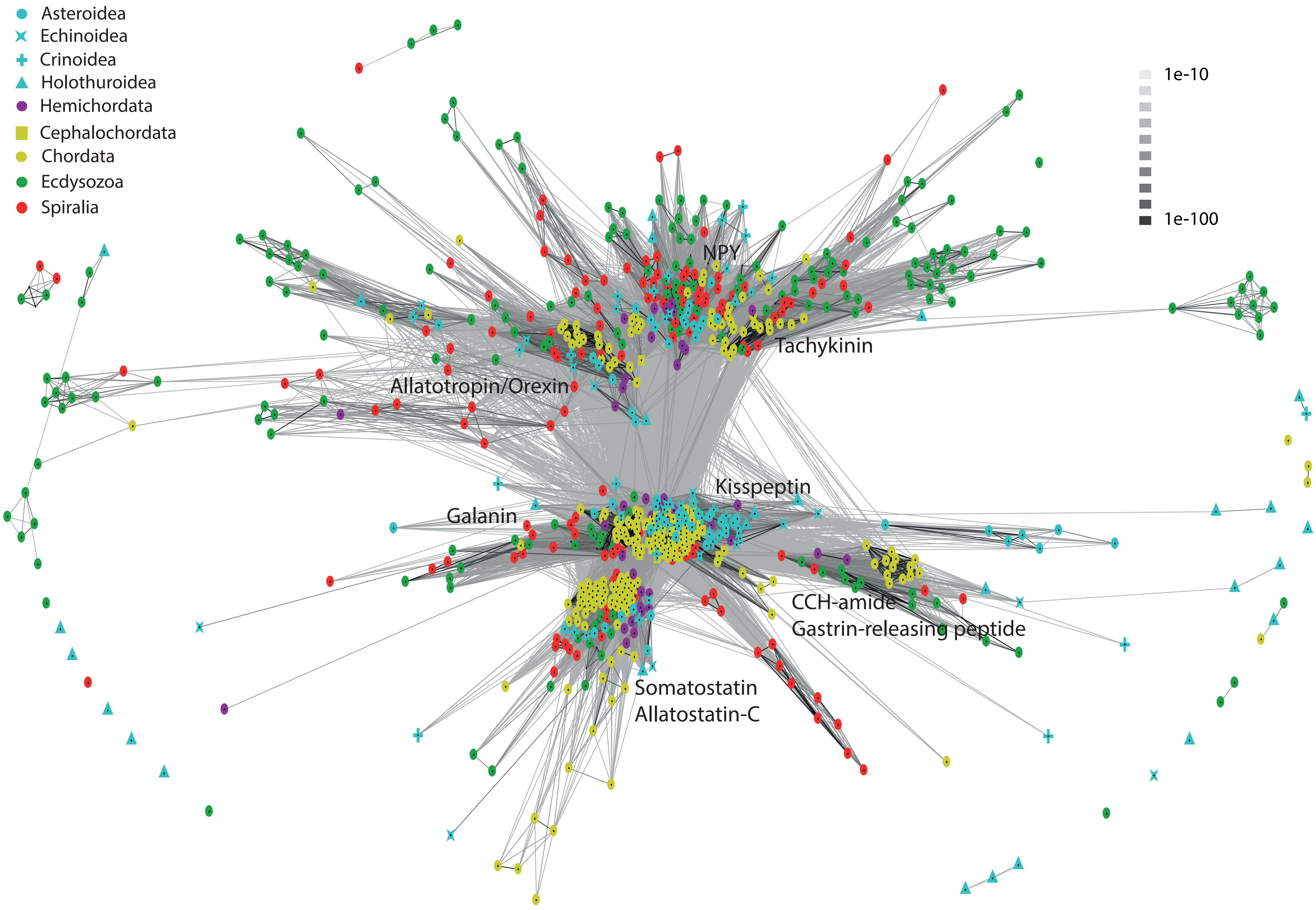

Asteroidea
Echinoidea
Crinoidea
Holothuroidea
Hemichordata
Cephalochordata
Chordata
Ecdysozoa
Spiralia
1e-10
1e-100
NPY
Tachykinin
Allatotropin/Orexin
Kisspeptin
Galanin
CCH-amide
Gastrin-releasing peptide
Somatostatin
Allatostatin-C
