# Supplementary material for: Receptor deorphanization in an echinoderm reveals kisspeptin evolution and relationship with SALMFamide neuropeptides
Source: BMC Biol. 2022 Aug 24;20:187. doi: 10.1186/s12915-022-01387-z (PMC9400282; doi:10.1186/s12915-022-01387-z)
Supplement: Supplementary file 8 — Additional file 8. CLuster Analysis of Sequences (CLANS) of A. rubens kisspeptin-type receptors (ArKPR1-11) and other kisspeptin-type receptors from bilaterian taxa. BLOSUM62 cluster map of kisspeptin-type receptors and the closely related galanin/allatostatin-A-type receptors. Nodes are labelled with taxon-specific shapes and colors, as shown in the key. Connections represent BLAST relationships with a P value > 1e-40. Galanin/allatostatin-A-type receptors are enclosed within the dashed line. Names shown in dark blue with an associated symbol containing a blue dot are the receptors for which neuropeptide ligands have been identified experimentally in this study or others. Names shown in black with an associated symbol containing a black dot are A. rubens kisspeptin-type receptors for which neuropeptide ligands have yet to be identified. The accession numbers for the receptors shown in this figure are provided in additional file 3. [file 12915_2022_1387_MOESM8_ESM.pdf]

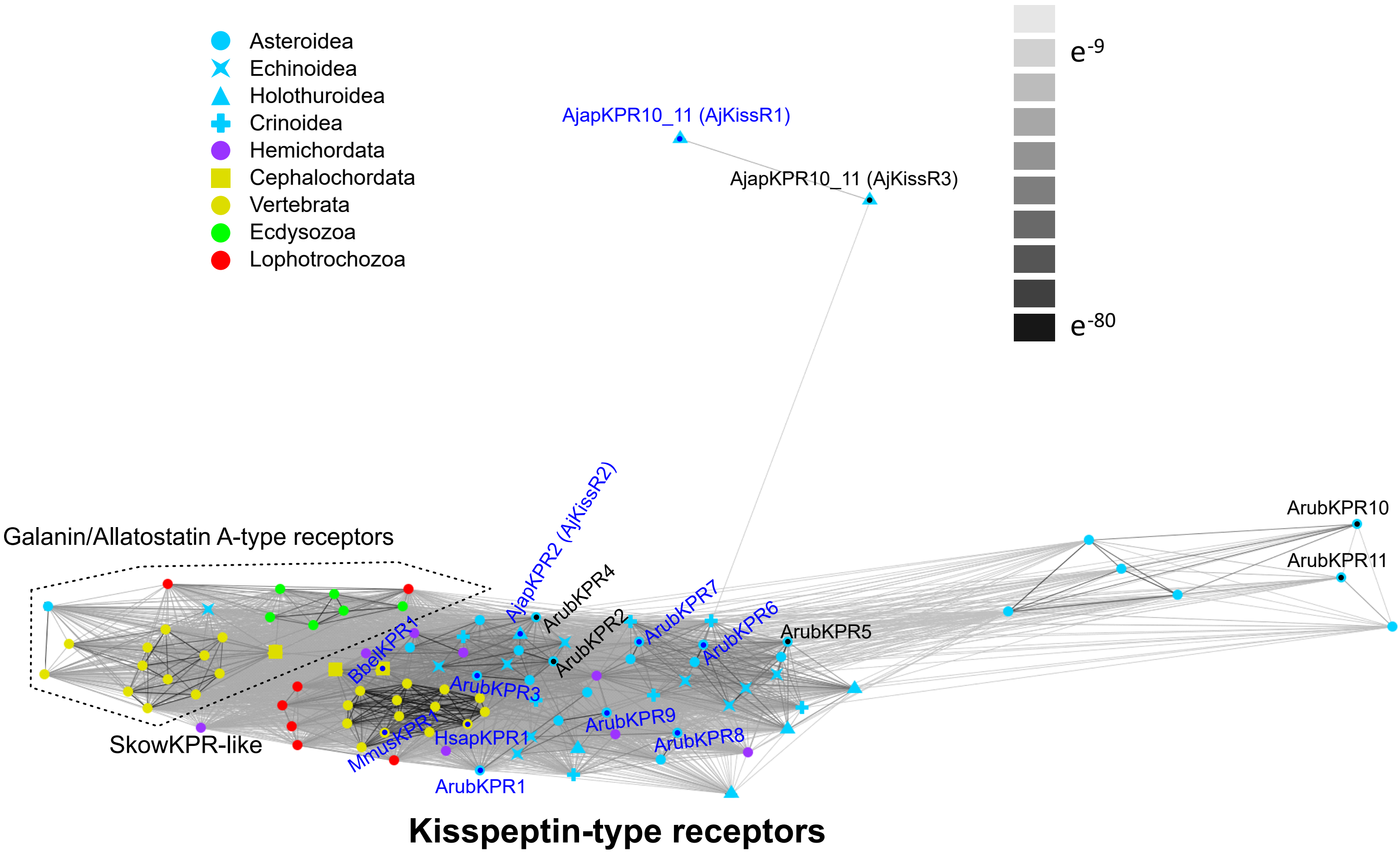
Asteroidea
Echinoidea
Holothuroidea
Crinoidea
Hemichordata
Cephalochordata
Vertebrata
Ecdysozoa
Lophotrochozoa
e-9
e-80
AjapKPR10_11 (AjKissR1)
AjapKPR10_11 (AjKissR3)
Galanin/Allatostatin A-type receptors
AjapKPR2 (AjKissR2)
ArubKPR4
ArubKPR2
ArubKPR7
ArubKPR6
ArubKPR5
ArubKPR10
ArubKPR11
BbelKPR1
ArubKPR3
ArubKPR9
ArubKPR8
HsapKPR1
MmusKPR1
ArubKPR1
SkowKPR-like
Kisspeptin-type receptors
