# Supplementary material for: Receptor deorphanization in an echinoderm reveals kisspeptin evolution and relationship with SALMFamide neuropeptides
Source: BMC Biol. 2022 Aug 24;20:187. doi: 10.1186/s12915-022-01387-z (PMC9400282; doi:10.1186/s12915-022-01387-z)
Supplement: Supplementary file 13 — Additional file 13. Sequences of chordate kisspeptin-type precursors (A) and precursors of neuropeptides that are candidate ligands for kisspeptin-type receptors in echinoderms (B-D). The N-terminal signal peptide is shown in blue, the neuropeptides predicted or shown to be derived from these proteins are shown in red (with the C-terminal glycine that is a substrate for amidation shown in orange) and monobasic or dibasic cleavage sites are shown in green. The neuropeptides (red) are named in accordance with the precursor they are derived from and their relative position in the precursor, which explains the nomenclature used in Fig. 3. Species names are abbreviated as follows: Hs, Homo sapiens, Lo, Lepisosteus oculatus, Bf, Branchiostoma floridae; Ar, Asterias rubens; Sp, Strongylocentrotus purpuratus; Aj, Apostichopus japonicus. [file 12915_2022_1387_MOESM13_ESM.pdf]

Chordate precursors of kisspeptin-type peptides

HsKPP1 NP_002247.1
MNSLVSWQLLLFLCATHFGEPLEKVASVGNSRPTGQQLESLGLLAPGEQSLPCTERKPAATARLSRRGTSLSPPP
HsKP1
ESSGSPQQPGLSAPHSRQIPAPQGAVLVQRE**KDLPNYNWNSFGLRFGKR**EAAPGNHGRSAGRG

LoKPP1 XP_015197899.1
MGHTGKEMPCSPLRTESTMLLLTMMLMMSVQLVEPWAGHPQISSASPTVSGKKPEPGVQDILRRMSTTPPPGARL
LoKP1
ILPAAGKIPPALASLLFGSRFPRRGGWAQARPQPPAA**KREKNLSAYNWNSFGLRYGKR**RSNTPPPQLG

LoKPP2 XP_015207582.1
MNRFLLLLAAVVVCQQRTMGKPLSGSSPAEQFQNSASTPAGLGSLPHLAVREVEGPNSADQASLCYFVQESEVES
LoKP2
QISCKL**RFTRSKFNFNPFGLRFGKR**NRNIVANDRSAIPSELLLYLLYLKETGLAP

LoKPP3 XP_015195061.1 LoKP3.1
MRLPAISLLVAAVCLGVQSADPSQFLPLPLRTLRYRNVDSAPPPLPVDAPGLLASRLEGDS**RRQAGEPYNVNSFG**
LoKP3.2
LRFGKKRDTQISWISSGLRSGGKRGRPERGEA

BfKPP1 XP_002608257.1
MRTTAFLLASLLVLLHTILPSTVDSLALGGREAQHRQDCGGRRIRSRVPVGLKRAWPDRRGGLNSDDIFWEDTLP
BfKP1
NSIERRQPSTEIDDEEVVDSADPD**RVQYNPNAWSRFGR**SMCP

BfKPP2 XP_002591617.1
MAVEAQMTSRAVVVAVLLVSMATSHPAAHAHLVSWAVPAKIEAQPAPQYEDVADSNSLALEAHEPLLEALSPESQ
BfKP2
PRHPASQPDSYRPLLIRLPWPLARALS**KRARKPPNMNAWGQPWGKR**DLSLVN

BfKPP3 XP_002608256.
MSPHIPGKTMVALLLILAAVASESRFAHKLPSFPGLQQTSGPYGPREPATTPLRSFTQESRWQPWKPAGVVQTSE
BfKP3
GSSAGFGLLHEWFRIGS**KRSGGAWVADTNMDDISPNMFSLHGKR**NVS

BfKPP4 XP_002608492.1
MMKRRLLVAVLLLVACSMGCRGTQRDILLPHGAREDTAKSGDGRAVHDAVLLREVLTHGPTIEALVRSTMTTVLV
ATGWMYKMMHGKVPGLDGSSRRGLQQGRRQGEEASTLHGILTSNIATTEQNSTSTARQTTETEVSTSHTA**RSGWE**
BfKP4.1 BfKP4.2
YENEPPEYNPNSWSVFGRMVSPQASPAPPEQDDEPGVPGMPVLAEMPP**KAAANLNMWSSFGRR**SAADRIPERTRD
BfKP4.3
GSPV**RSSSVNPAFFLTPFG**DQTDKQRSIEERQVKQTGDAAASHKRTPDPRVYPPNARPTLQPDWTKIPFFG

Echinoderm precursors of kisspeptin-like peptides

ArKPP1 ALJ99947.1
MEWFTKCCLLVILAVCFGSSFVLGDGRNLQGYNGDLYNGEFENEEGTEALRNIIGQIIDDVDAKNNIRTAILEDT
ArKP1.1 ArKP1.2
LEHAQYEPDKRSGRCRSGTKCIM**RGPNPNTASRVLPFGKR**EDDSPNKLARRGRGPP**KNSRARGGRTLLPFGKRR**

SpKPP1 XP_003725009.1
MMEVRTSVTAVTVLLALLCAVATGDDYVPSKLYADSSPGSMYDVDGVATQPDDDNDVDDLLMTVIARLIQNIDPN
SpKP1.1
KLDKASTHRLLAAITGLNPPQQIHLSKALLDENLDPANADFMIPDDDKRSRCRGRQC**RNVGGLNPNANLRPLPFG**
SpKP1.2
KRSRFSGASQHQGSLSSSSLGDFSN**KRGRTKNRIRERVPHFLPFGRR**

AjKPP1 AWU78782.1
MDKIVFPILLSLLCGTVFSASLADTNLKDYEDRLDDARERVLKLIAGLLSDDTYQEQFTGEQDEDDLAVNIPILE
AjKP1.1
NLLAENDGEDVIDADDTAELIFESLSNNGRPIDEKRAGSLDCLEASCEDVE**RRGRQPNRNAHYRTLPFGKR**VQRQ
AjKP1.2
TFSTVRNT**RKSAVKNKNKSRARPPLLPFGK**

ArKPP2 ALJ99949.1
MLLAMAPNGEMMTRFLLAAHFLLLAVSIVNARVYFNGEDETKSGLLELSEYGENEKVDGTEDVDGQQVEDRQWKG
ArKP2.1
EDQWKSGLYAAQRSLQSYPNTAKRSWPQTGMYNKQSTNWLRALAQEPRWHSAMA**KRQLWANQQSGLFGKR**EADME
ArKP2.2
RTLPAWNVKRSAEEREFARQS**RGGGVPHVFQSGGIFGKR**SSDDWAKRYE

SpKPP2 XP_011683115.1
MALNRLKVACCVVFVYLCLALLTSAKEFSPLTGDDFAKNYDPQPWGVDNPAPMNLQDPQNTFYNEADVGKELDEK
SpKP2.1
RGTKNDRLRGLRRLSRLYGELGLDRLRPVKKTPETADDMYSLDDKRNDAGMP**KRDAGPHAWYGTGMFGKR**TDENR
SpKP2.2
GRWRI**RSPRPQPYRVGLFGKR**DFETVDMNELAQIIEAIREDESRR

SpKPP3 XP_011679113.2
MAYFLRSMIFNVIITSVLLALFIGTVPSQTANAYDEVKQWHNELLDDPDTTYQHIESAGKNDEKRNAFNDKLRGL
RRLSRIIDSLGFESDIRPAKKSPEIVDKLDNANGNGGRQIVSSGHIINQNSGVIDDAIPLVGDGLRNVASPQDMD
SpKP3
GRQTPSDQQEGWKLKSRVTTNADEPKETGVPKRSALGLEAFRQKSRES**RRRWGGKASRPQPFRIGLFGKR**DAAET
LGMINTSDLADIIAEMLNGDPNNNQPSQTD

AjKPP2 AXU40272.1
MANLYYISISLIAVVVLSSVICVLADGIDGESPLAKRRRLSIEKLRNTGRLRRIFKKRFDENEFQGVDDEDELMS
AjKP2.1 AjKP2.2
PTDFLDFLDELEALERLKENKIEYEKRGNA**RREGSRKMNGWYTGMFGKR**SSAASI**RETRNIPQTYLSGDYFGKR**F
SDESPAEGLDWKRTYNLEDTAEDLE

Echinoderm precursors of L-type SALMFamide peptides

ArL-SALMFa ALJ99974.1
ArS1.1
MKGQHLLAVAVVVVAGSFGIIEAYSPFGGYNRAPFDNVWVRADSMARGGSTGEDEANEQRMTGA**KRPAGASAFHS**
ArS1.2 ArS1.3 ArS1.4 ArS
ALSYGKRGDDDSAEVE**RRAYHSALPFGKR**TPIE**KRAYHTGLPFGKR**DDEAAEQDAMME**RRGFNSALMFGKRLHSA**
1.5 ArS1.6 ArS1.7
LPFGKRGYHSALPFGKRLDTTDEGDIIE**RRGYHSGLPFGKR**ATDDEAVNDILDQLRSEEN

SpL-SALMFaP EC439101. SpS1.1
MQVQQITVFLVACTLSVLVVAYAQEDAETVLLNRLRDIAARAAAGELPDFFADVDDYKRGG**KKNMGSIHSHSGIH**
SpS1.2
FGKRRDSESSERARNT**KRMRLHPGLLFGKR**APVQKWDQWQAQDTYNPDWELGQFN

AjSALMFaP AWU78784.1
MKAYQIIVPAVMCVLAAILARTEADGELRILNNRLFELTKELEERLREQQLEDADLILTEDGDQEIGM**KKVVSRA**
AjS1.1 AjS1.2
WSPLVGQTGIAFGKRTDGLDRARSQTDQRA**KKTRSRSMFGNTALPFGKR**AGYIPHAQEIWDLQDAANNLDTFEEV
AjS1.3
PV**KKRMGFTGNTGILLGKR**NADDAQE

Echinoderm precursors of F-type SALMFamide peptides

ArF-SALMFaP ALM01494.1

ArS2.1
MMVRFVALLGAVSLLVCQSAGLDAADVEEQDEFNKPYAPDSSYADLNALLGNNVPSLHSAS**KRQQSDREREVEAA**
ArS2.2 ArS2.3
QTQFYPYGRRTDPRKASGGFTFGKRGQYFIPIPYEKREMDEVNPYSVAKRDDELTGLEEYQAS**KRSGPYSFNSGL**
ArS2.4 ArS2.5 ArS2.6 ArS2.7 ArS2.8
TFGKREPE**KRNIFGSYDFGKRAYGNNFSFGKRGMGVSSFSFGKR**SGLEGEQMMPED**KRAFGDFSFGKRNNGLSSF**
TFGKREGER

SpF-SALMFaP XP_003724375.1
MLFTMRVLVALALCLCFIAPSPVLSFTMPEEKFVENKMADVGEEGTGQNNINSIAKSLIREVFGAAEEREMEAEN
SpS2.1
EAEDEAELSLSKRTTGSTRPQREIRARAQYAA**RRPPVTTRSKFTFGKR**SSPTPVISRPLAEQLLEELQRNAEMSD
SpS2.2 SpS2.3 SpS2.4 SpS2.5 SpS2.6
DWRESDKLALLNDAALYDSLVDSHQVQ**KDAYSAFSFGKRGMSAFSFGKRAQPSFAFGKRGLMPSFAFGKRPHGGS**
SpS2.7
AFVFGRRDWAPREQDFANAAEESGPY**KRGDLAFAFGKR**EDQ

AjF-SALMFaP MF401999.2
MAPPSIFLLISVSFLAVLHPSHTETLDHTGAIVKEIQMELPHDMETAQLLLQGQELRDLADELALDYLNERSDDD
AjS2.1 AjS2.2 AjS2.3
DTFSNIAKREPGRSSTTNNKDTMYEPFV**RRGVPPYVVKVTYGKR**SDD**KRFKSPFMFGKR**EDLNGLD**KRGYSPFMF**
AjS2.4 AjS2.5 AjS2.6 AjS2.7
GKREMAQPHLSE**KRARYSPFTFGKR**DGADDEDENLEEV**KRGGYSALYFGKRVPELAESDGGQSKLYFGKRGHRGG**
AjS2.8
QFSQFKFGKREDGALGMDANEDDEMEQNFEKKDAITQN**KRFKSSFYLGKR**NYVAENEDMEDLQDV
