# Supplementary material for: Receptor deorphanization in an echinoderm reveals kisspeptin evolution and relationship with SALMFamide neuropeptides
Source: BMC Biol. 2022 Aug 24;20:187. doi: 10.1186/s12915-022-01387-z (PMC9400282; doi:10.1186/s12915-022-01387-z)
Supplement: Supplementary file 18 — Additional file 18. Mass spectra for peptides derived from the A. rubens precursor proteins ArKPP1, ArKPP2, ArL-SALMFaP and ArF-SALMFaP as detected in radial nerve cord extracts. [file 12915_2022_1387_MOESM18_ESM.docx]

Ai


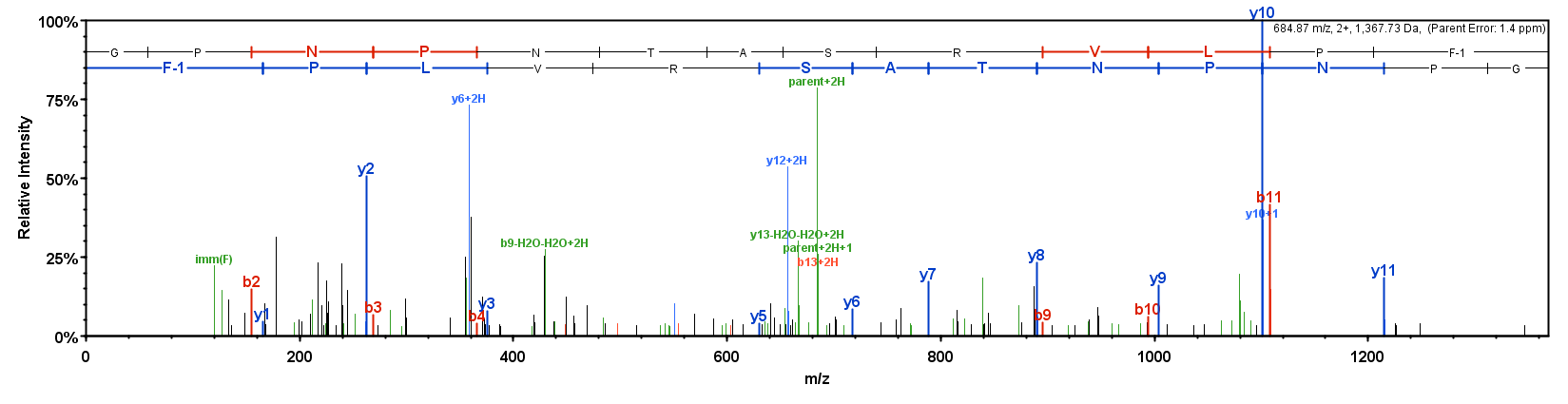


Aii


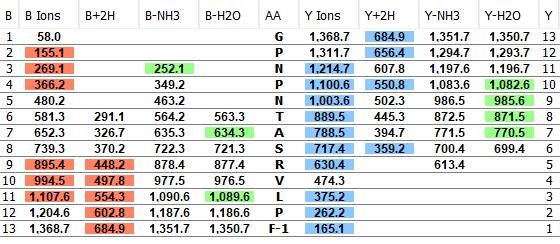


Supplementary Figure 4A (i) Mass spectrum derived from MS/MS analysis of the peptide ArKP1 (GPNPNTASRVLPF-NH_2_) present in a methanol acetic acid extract of radial nerve cords from *Asterias rubens*. (ii) MS/MS data is shown with the b series of fragment ions in red, fragment ions from the y series in blue, and other identified fragment ions in green.

Bi


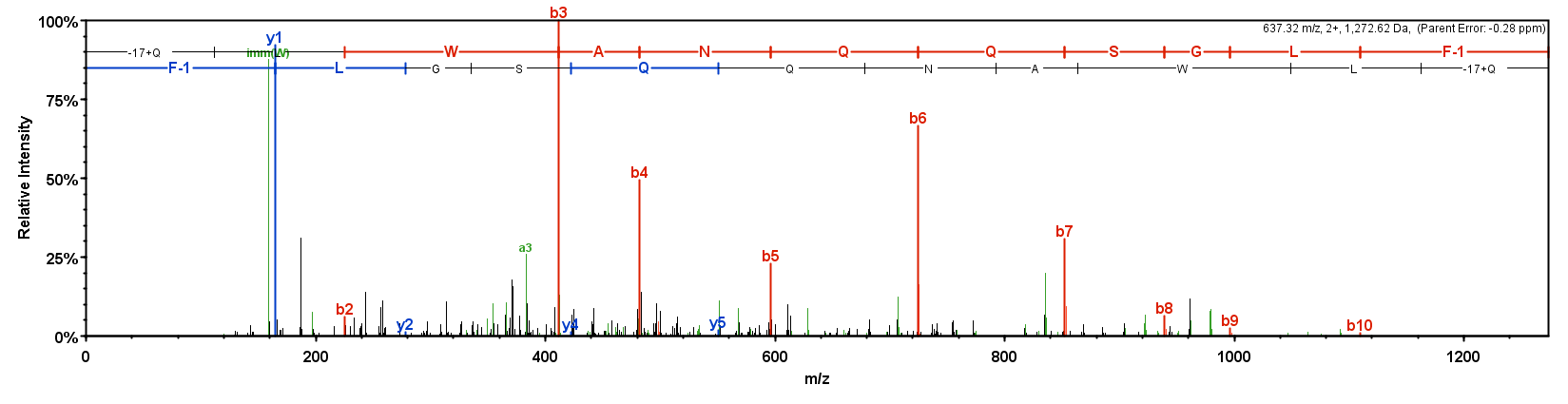


Bii


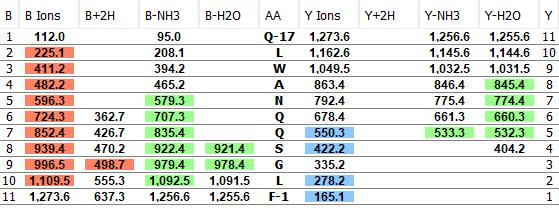


Supplementary Figure 4B (i) Mass spectrum derived from MS/MS analysis of the peptide ArKP2.1 (ArKP2.1; pQLWANQQSGLF-NH_2_) present in a methanol acetic acid extract of radial nerve cords from *Asterias rubens*. (ii) MS/MS data is shown with the b series of fragment ions in red, fragment ions from the y series in blue, and other identified fragment ions in green.

Ci


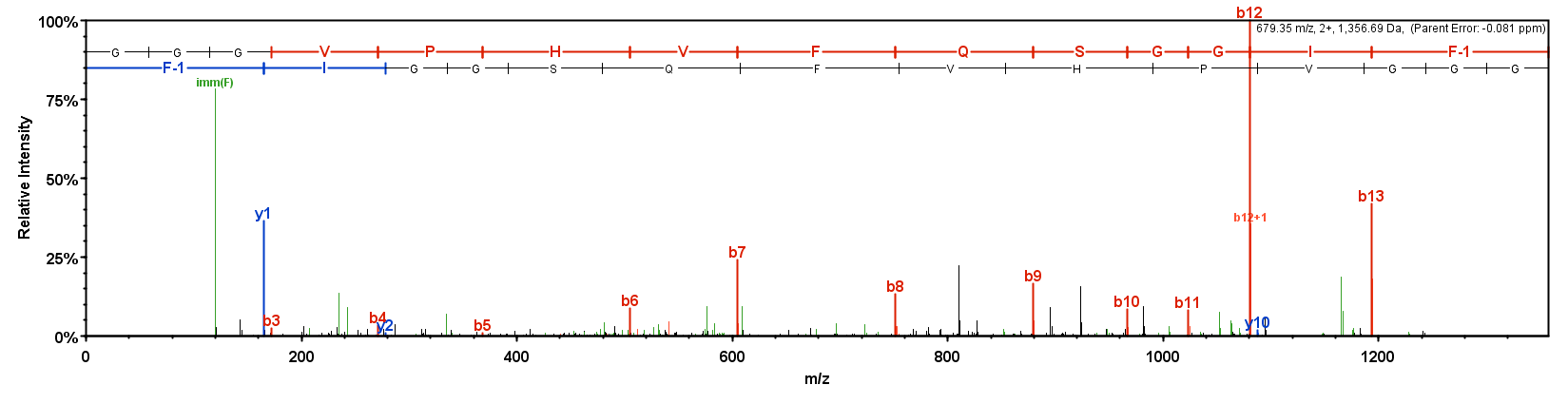


Cii


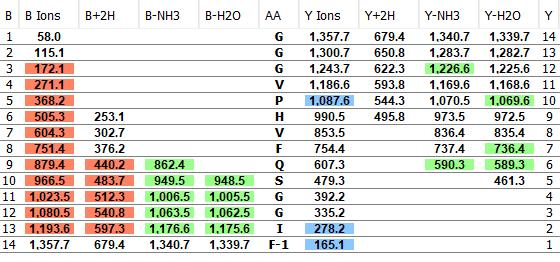


Supplementary Figure 4C (i) Mass spectrum derived from MS/MS analysis of the peptide ArKP2.2 (ArKP2.2; GGGVPHVFQSGGIF-NH_2_) present in a methanol acetic acid extract of radial nerve cords from *Asterias rubens*. (ii) MS/MS data is shown with the b series of fragment ions in red, fragment ions from the y series in blue, and other identified fragment ions in green.

Di


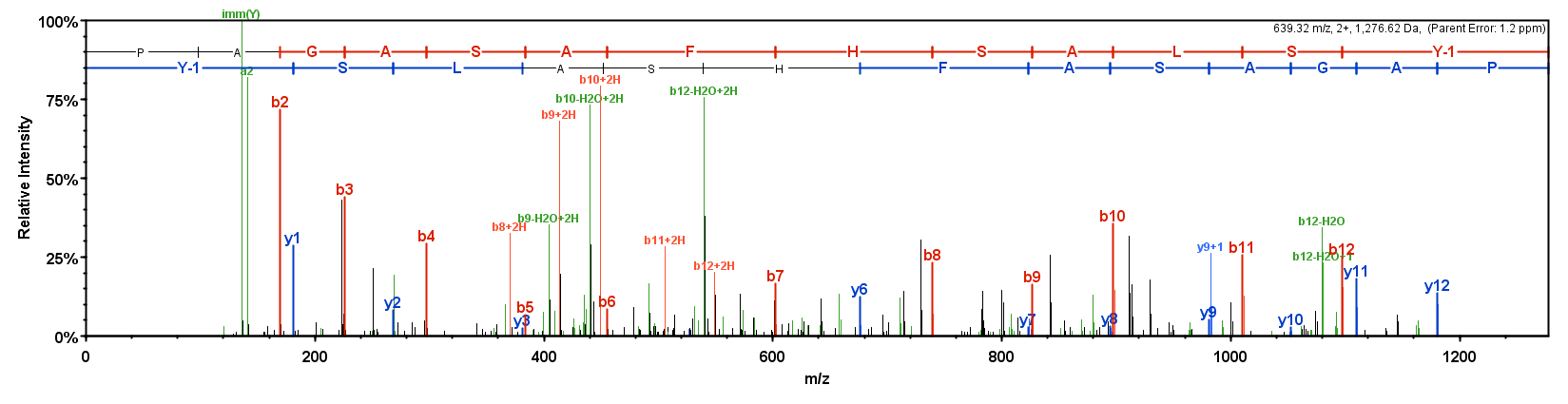


Dii


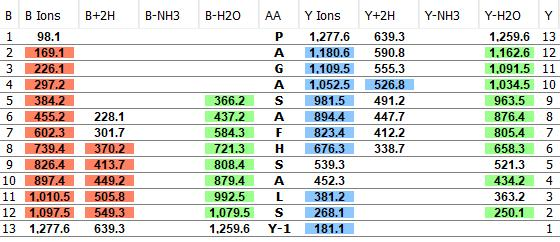


Supplementary Figure 4D (i) Mass spectrum derived from MS/MS analysis of the peptide ArS1.1 (PAGASAFHSALSY-NH_2_) present in a methanol acetic acid extract of radial nerve cords from *Asterias rubens*. (ii) MS/MS data is shown with the b series of fragment ions in red, fragment ions from the y series in blue, and other identified fragment ions in green.

Ei


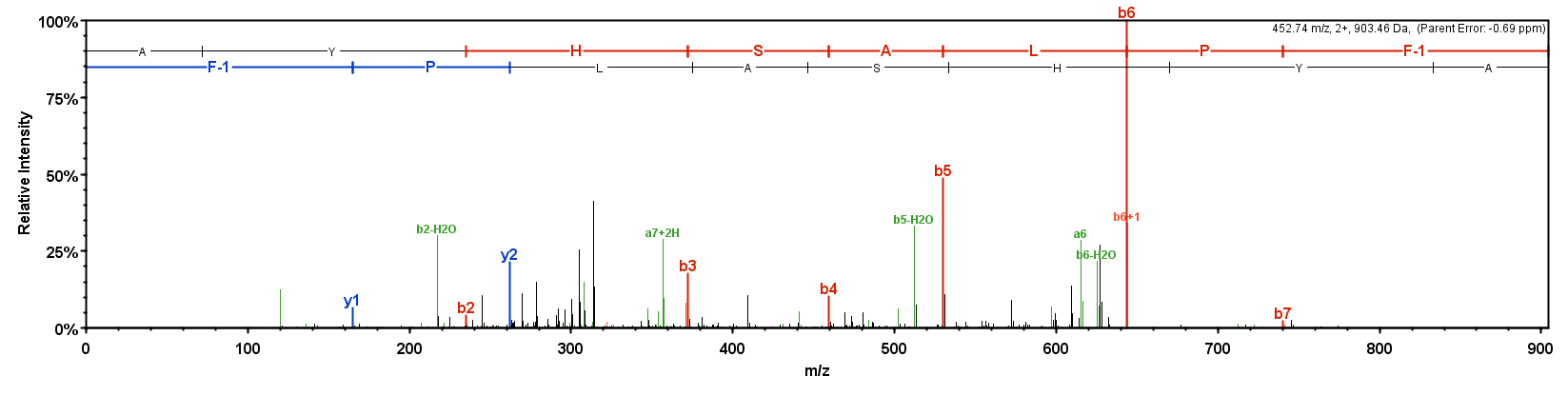


Eii


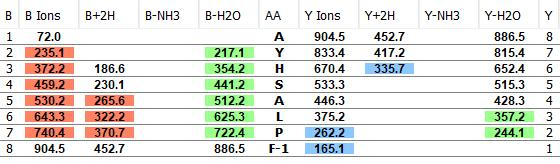


Supplementary Figure 4E (i) Mass spectrum derived from MS/MS analysis of the peptide ArS1.2 (AYHSALPF-NH_2_) present in a methanol acetic acid extract of radial nerve cords from *Asterias rubens*. (ii) MS/MS data is shown with the b series of fragment ions in red, fragment ions from the y series in blue, and other identified fragment ions in green.

Fi


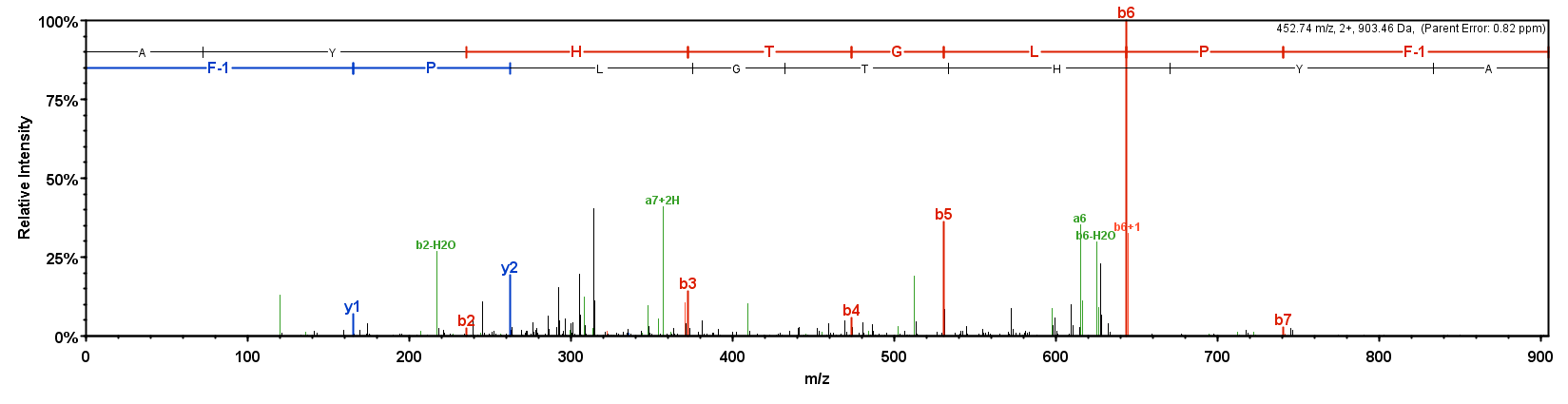


Fii


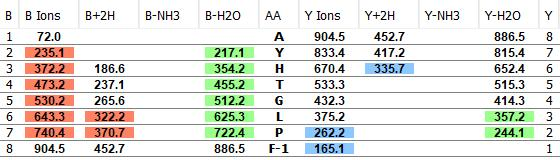


Supplementary Figure 4F (i) Mass spectrum derived from MS/MS analysis of the peptide ArS1.3 (AYHTGLPF-NH_2_) present in a methanol acetic acid extract of radial nerve cords from *Asterias rubens*. (ii) MS/MS data is shown with the b series of fragment ions in red, fragment ions from the y series in blue, and other identified fragment ions in green.

Gi


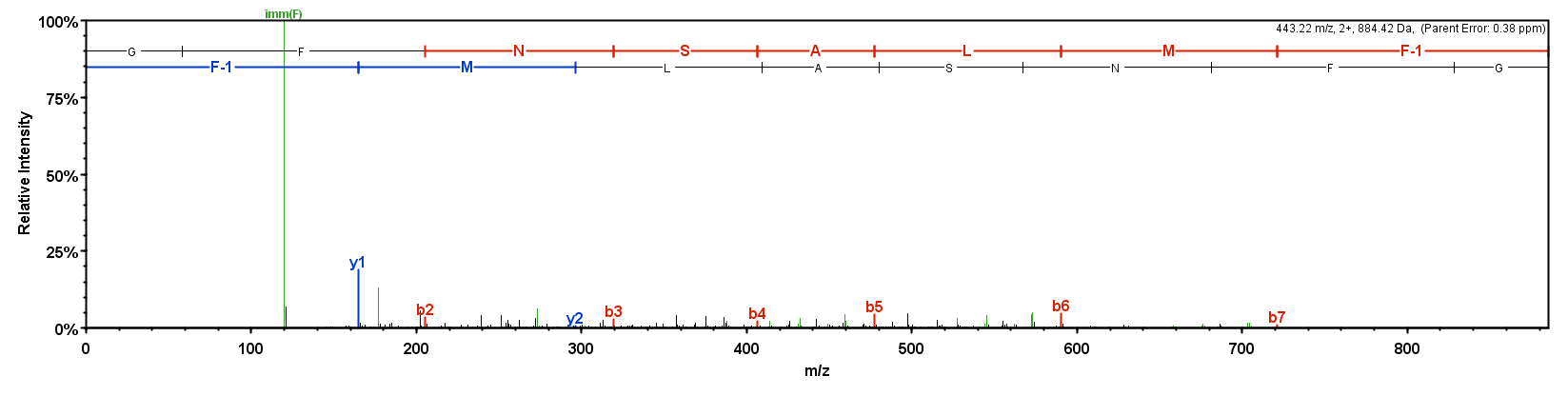


Gii


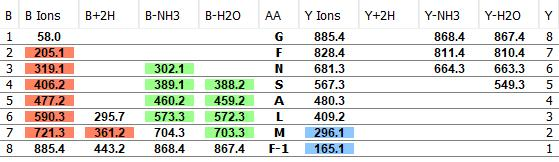


Supplementary Figure 4G (i) Mass spectrum derived from MS/MS analysis of the peptide ArS1.4 (GFNSALMF-NH_2_) present in a methanol acetic acid extract of radial nerve cords from *Asterias rubens*. (ii) MS/MS data is shown with the b series of fragment ions in red, fragment ions from the y series in blue, and other identified fragment ions in green.

Hi


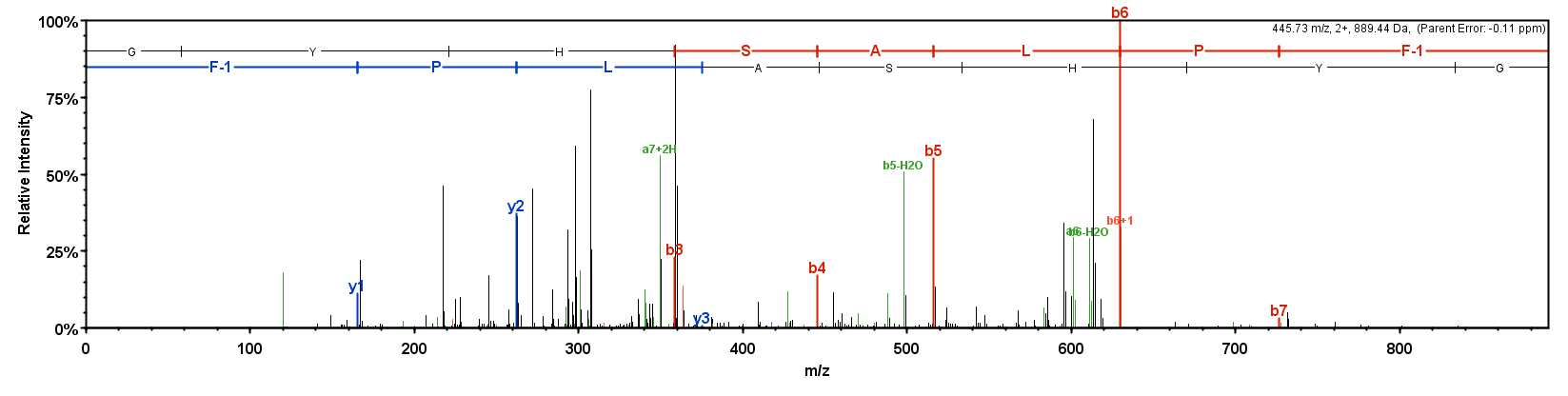


Hii


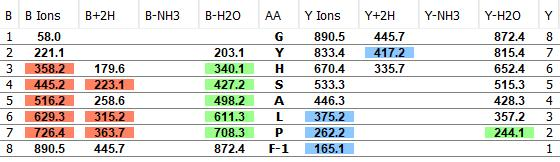


Supplementary Figure 4H (i) Mass spectrum derived from MS/MS analysis of the peptide ArS1.6 (GYHSALPF-NH_2_) present in a methanol acetic acid extract of radial nerve cords from *Asterias rubens*. (ii) MS/MS data is shown with the b series of fragment ions in red, fragment ions from the y series in blue, and other identified fragment ions in green.

Ii


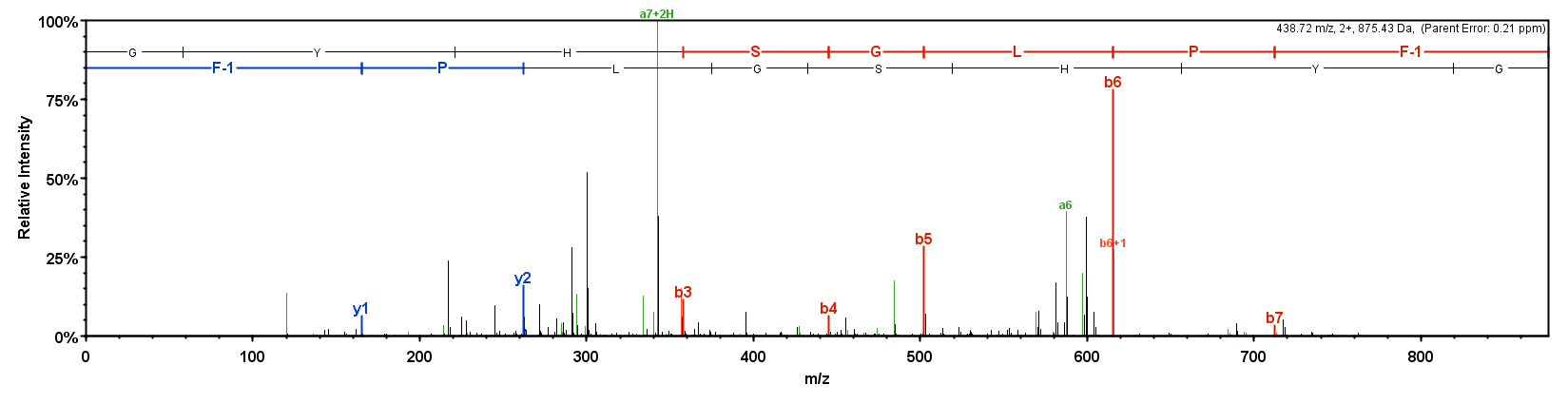


Iii


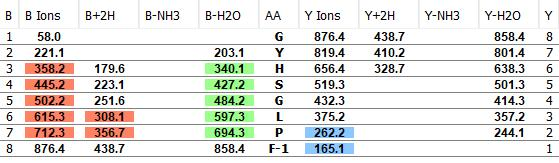


Supplementary Figure 4I (i) Mass spectrum derived from MS/MS analysis of the peptide ArS1.7 (GYHSGLPF-NH_2_) present in a methanol acetic acid extract of radial nerve cords from *Asterias rubens*. (ii) MS/MS data is shown with the b series of fragment ions in red, fragment ions from the y series in blue, and other identified fragment ions in green.

Ji


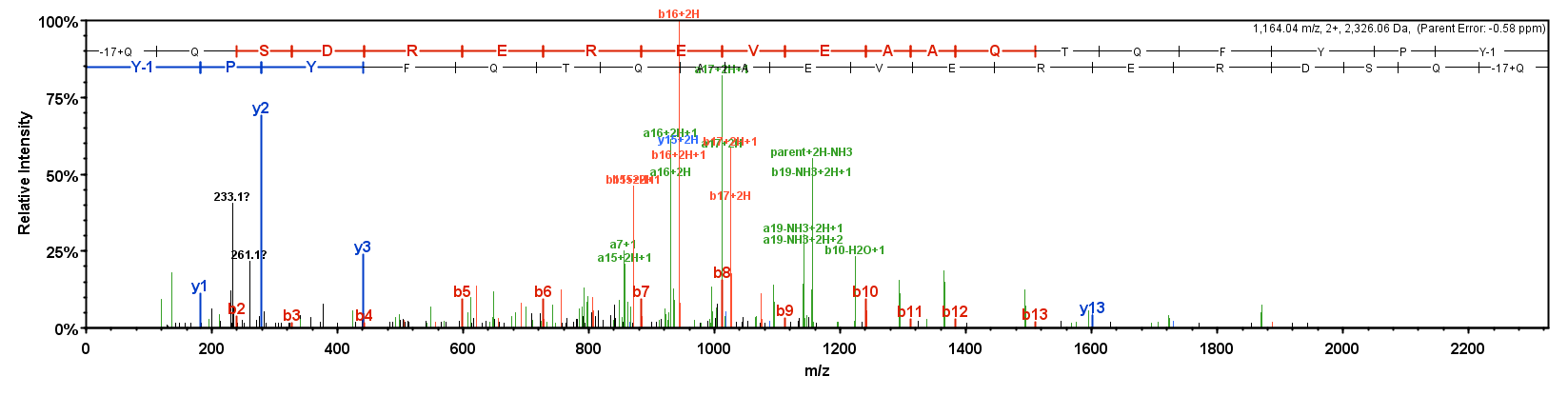


Jii


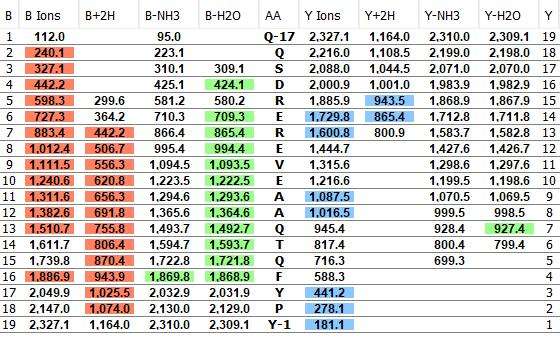


Supplementary Figure 4J (i) Mass spectrum derived from MS/MS analysis of the peptide ArS2.1 (pQQSDREREVEAAQTQFYPY-NH_2_) present in a methanol acetic acid extract of radial nerve cords from *Asterias rubens*. (ii) MS/MS data is shown with the b series of fragment ions in red, fragment ions from the y series in blue, and other identified fragment ions in green.

Ki


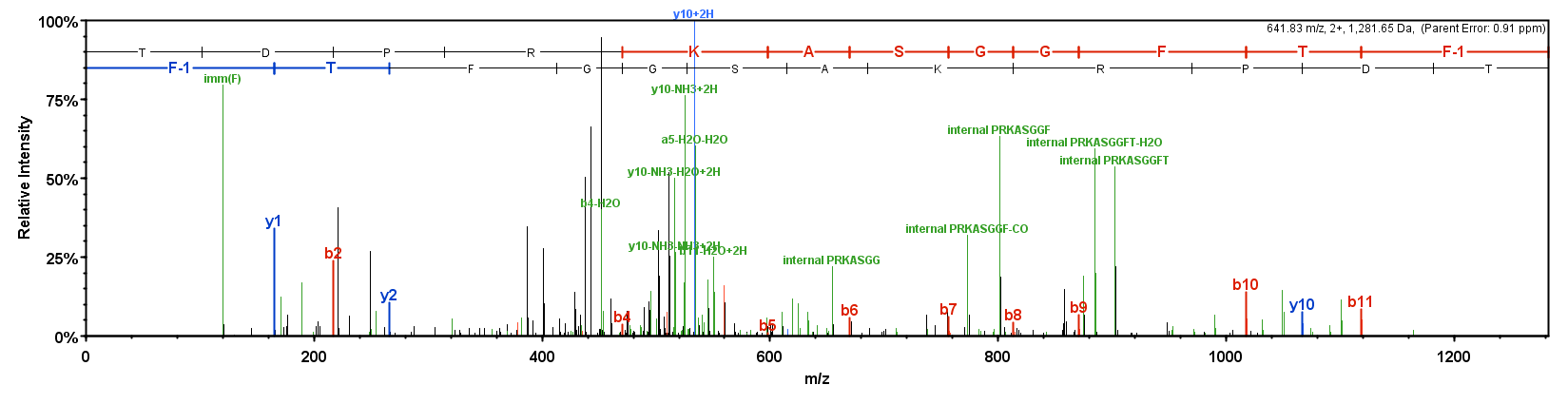


Kii


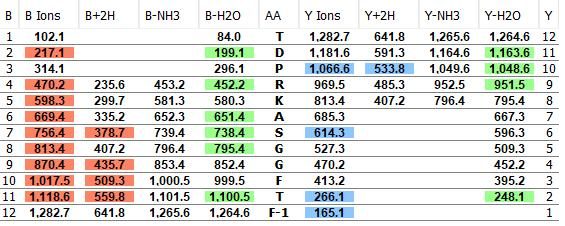


Supplementary Figure 4K (i) Mass spectrum derived from MS/MS analysis of the peptide ArS2.2 (TDPRKASGGFTF-NH_2_) present in a methanol acetic acid extract of radial nerve cords from *Asterias rubens*. (ii) MS/MS data is shown with the b series of fragment ions in red, fragment ions from the y series in blue, and other identified fragment ions in green.

Li


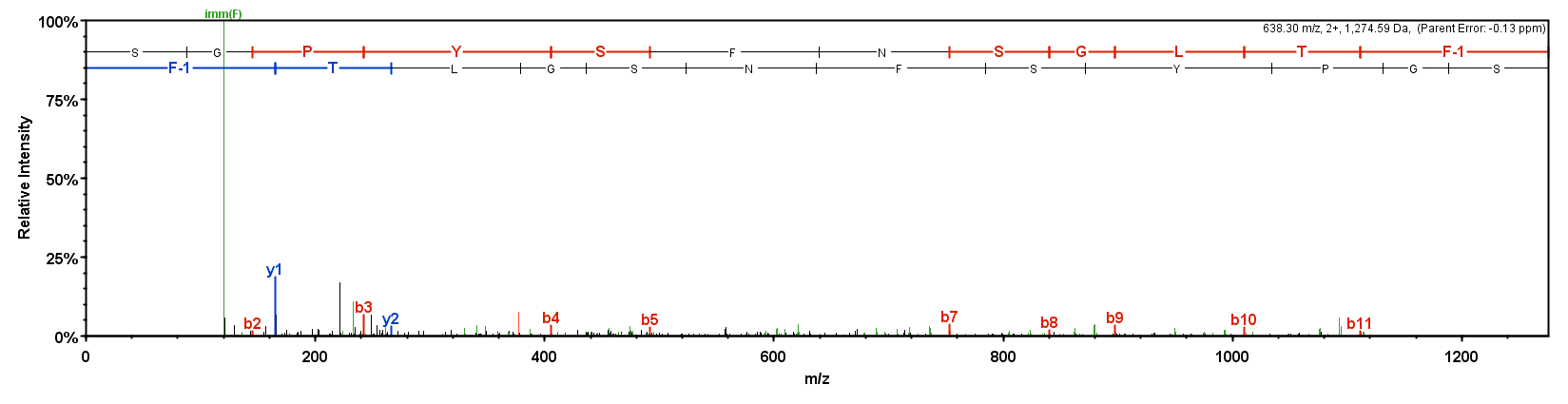


Lii


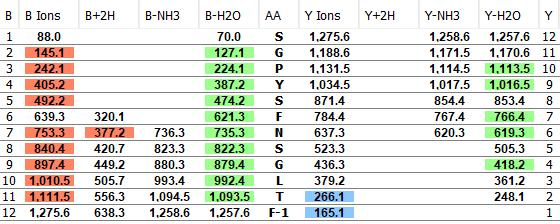


Supplementary Figure 4L (i) Mass spectrum derived from MS/MS analysis of the peptide ArS2.3 (SGPYSFNSGLTF-NH_2_) present in a methanol acetic acid extract of radial nerve cords from *Asterias rubens*. (ii) MS/MS data is shown with the b series of fragment ions in red, fragment ions from the y series in blue, and other identified fragment ions in green.

Mi


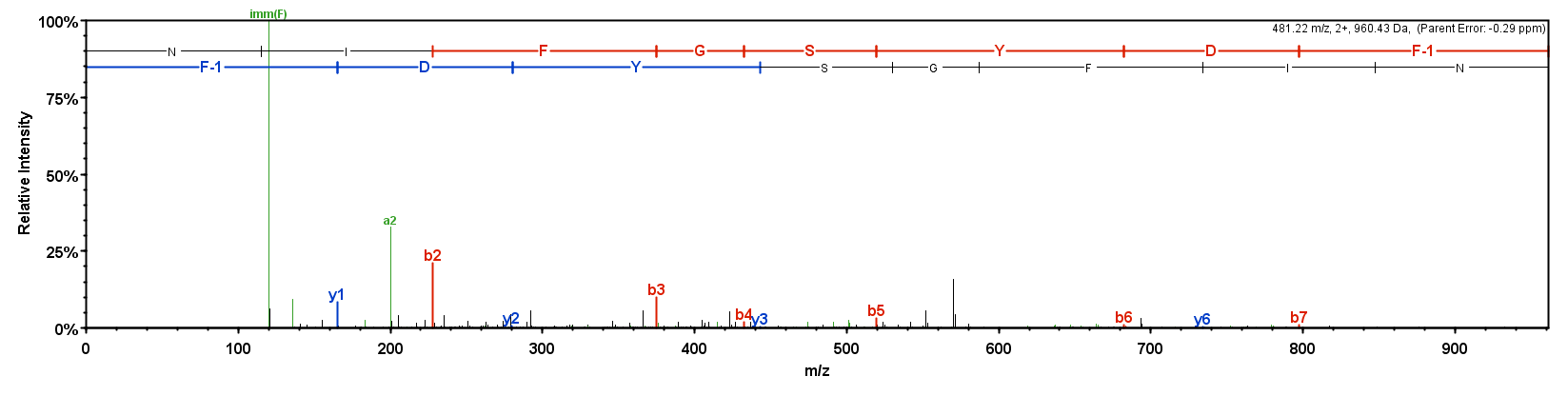


Mii


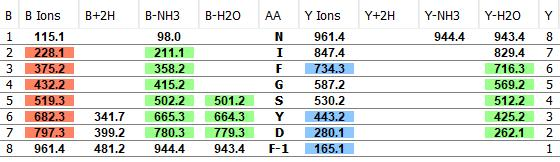


Supplementary Figure 4M (i) Mass spectrum derived from MS/MS analysis of the peptide ArS2.4 (NIFGSYDF-NH_2_) present in a methanol acetic acid extract of radial nerve cords from *Asterias rubens*. (ii) MS/MS data is shown with the b series of fragment ions in red, fragment ions from the y series in blue, and other identified fragment ions in green.

Ni


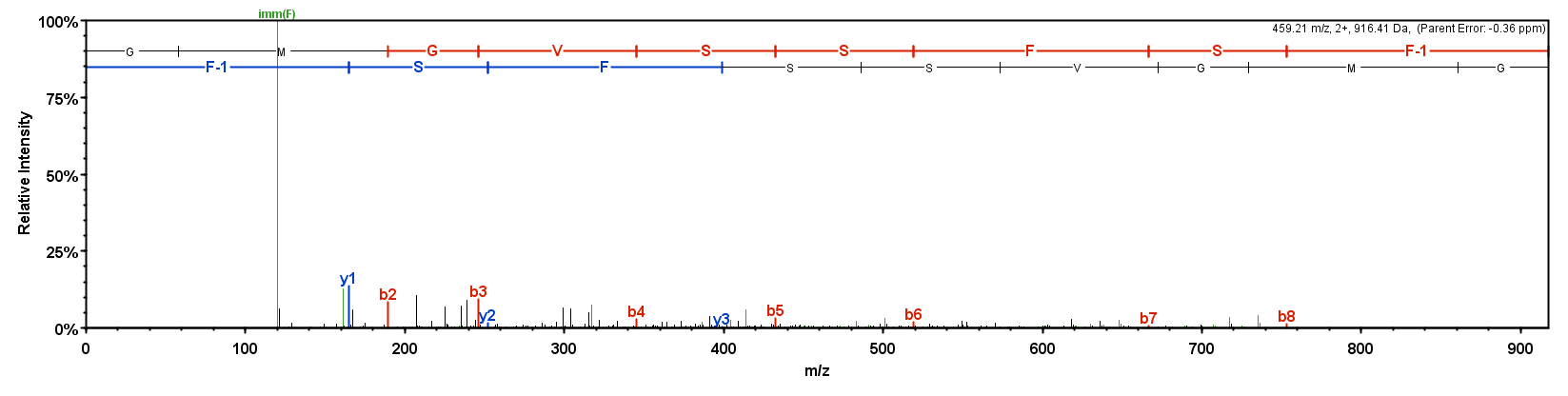


Nii


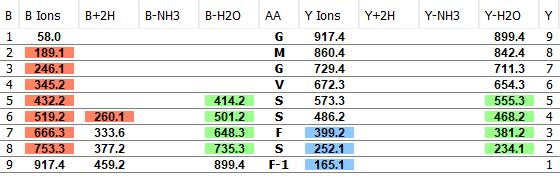


Supplementary Figure 4N (i) Mass spectrum derived from MS/MS analysis of the peptide ArS2.6 (GMGVSSFSF-NH_2_) present in a methanol acetic acid extract of radial nerve cords from *Asterias rubens*. (ii) MS/MS data is shown with the b series of fragment ions in red, fragment ions from the y series in blue, and other identified fragment ions in green.

Oi


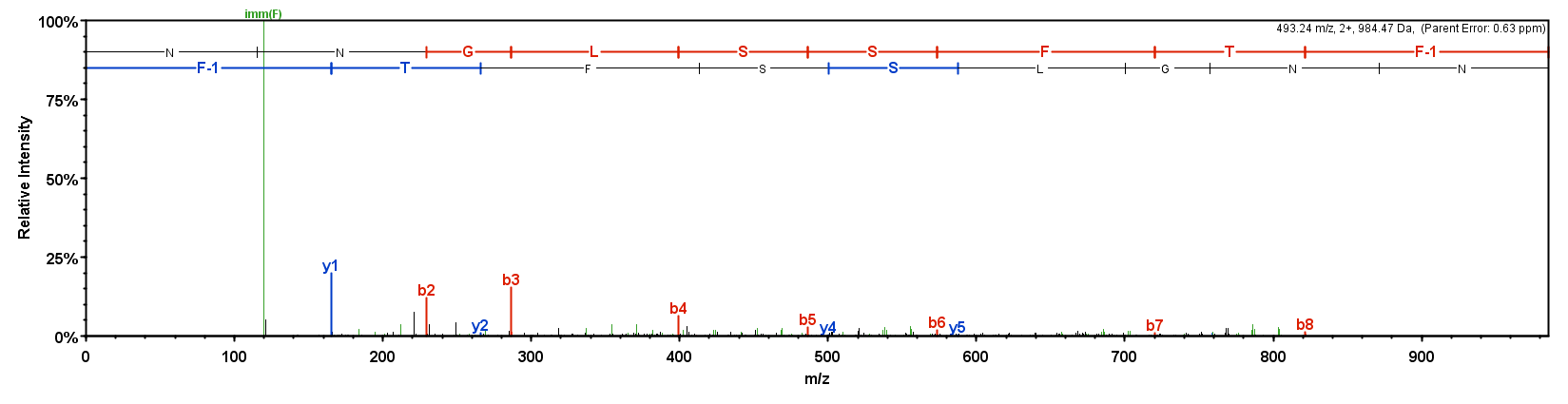


Oii


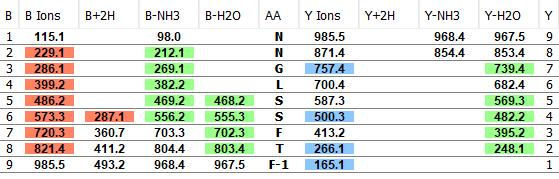


Supplementary Figure 4O (i) Mass spectrum derived from MS/MS analysis of the peptide ArS2.8 (NNGLSSFTF-NH_2_) present in a methanol acetic acid extract of radial nerve cords from *Asterias rubens*. (ii) MS/MS data is shown with the b series of fragment ions in red, fragment ions from the y series in blue, and other identified fragment ions in green.
